# Effect of Octamer-Binding Transcription Factor 4 Overexpression on the Neural Induction of Human Dental Pulp Stem Cells

**DOI:** 10.1007/s12015-024-10678-7

**Published:** 2024-02-05

**Authors:** Maria R. Gancheva, Karlea Kremer, James Breen, Agnes Arthur, Anne Hamilton-Bruce, Paul Thomas, Stan Gronthos, Simon Koblar

**Affiliations:** 1 Adelaide Medical School, Faculty of Health and Medical Sciences, The University of Adelaide, Adelaide, 5005 Australia; 2 School of Biological Sciences, Faculty of Science, Engineering and Technology, The University of Adelaide, Adelaide, 5005 Australia; 3 School of Biomedicine, Faculty of Health and Medical Sciences, The University of Adelaide, Adelaide, 5005 Australia; 4 Stroke Research Programme, Basil Hetzel Institute, The Queen Elizabeth Hospital, Central Adelaide Local Health Network, Woodville South, 5011 Australia; 5 South Australian Health and Medical Research Institute, Adelaide, 5000 Australia

**Keywords:** Dental pulp stem cells, Cellular reprogramming, Neural, Neuronal, Octamer-binding transcription factor 4, Cell-based therapy

## Abstract

Stem cell-based therapy is a potential alternative strategy for brain repair, with neural stem cells (NSC) presenting as the most promising candidates. Obtaining sufficient quantities of NSC for clinical applications is challenging, therefore alternative cell types, such as neural crest-derived dental pulp stem cells (DPSC), may be considered. Human DPSC possess neurogenic potential, exerting positive effects in the damaged brain through paracrine effects. However, a method for conversion of DPSC into NSC has yet to be developed. Here, overexpression of octamer-binding transcription factor 4 (OCT4) in combination with neural inductive conditions was used to reprogram human DPSC along the neural lineage. The reprogrammed DPSC demonstrated a neuronal-like phenotype, with increased expression levels of neural markers, limited capacity for sphere formation, and enhanced neuronal but not glial differentiation. Transcriptomic analysis further highlighted the expression of genes associated with neural and neuronal functions. In vivo analysis using a developmental avian model showed that implanted DPSC survived in the developing central nervous system and respond to endogenous signals, displaying neuronal phenotypes. Therefore, OCT4 enhances the neural potential of DPSC, which exhibited characteristics aligning with neuronal progenitors. This method can be used to standardise DPSC neural induction and provide an alternative source of neural cell types.

**Graphical Abstract:**

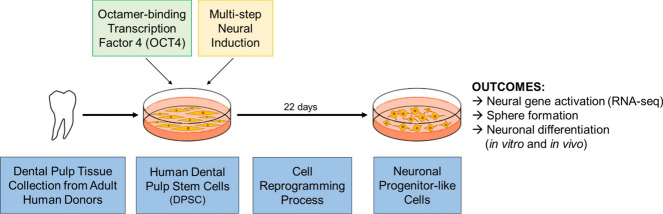

**Supplementary Information:**

The online version contains supplementary material available at 10.1007/s12015-024-10678-7.

## Introduction

Stem-cell based therapy presents an alternative therapeutic strategy for regeneration of the central nervous system (CNS) following severe injury or disease [1, 2]. Disruption to the CNS, following insult from trauma or neurological disease such as ischaemic stroke, can lead to irreversible cell loss resulting in functional deficits contributing to death or permanent disability [3]. This often leads to a significant health and economic burden to patients, families and carers, and health systems. With an aging population, the impact of neurological diseases is likely to increase, highlighting the importance in developing better treatments [2].

Regeneration of the CNS is a challenge, requiring multiple complex processes, which include modulation of the immune response, induction of neuroplasticity, and stimulation of neurogenesis [4]. There are no effective treatments that can restore damaged neural tissue and associated functions [2]. The currently available therapeutic interventions are limited to a narrow therapeutic window with stringent eligibility criteria, and are unable to sufficiently improve the disease outcome [2, 3, 5]. Beyond the acute stage, physical rehabilitation is relied upon to promote adaptation to residual disability [2]. Stem cells have the potential to act through multiple mechanisms to promote functional recovery beyond current therapies [1].

Neural stem cells (NSC), the resident stem cell population of the CNS, have the potential to differentiate into neurons, astrocytes and oligodendrocytes. However, the response of endogenous NSC is limited with age, and in hostile environments consisting of strong inflammatory responses initiated by injury or disease [6]. In vivo transplantation of NSC has been shown to stimulate functional improvements via paracrine effects, and in addition to differentiating into neurons and astrocytes, has induced endogenous NSC to proliferate [7–14]. Isolating sufficient numbers of NSC from tissues for clinical application however poses a challenge [15]. In addition to isolating NSC from foetal or adult tissues, they can be differentiated from embryonic stem cells (ESC), induced pluripotent stem cells (iPSC), or directly differentiated from other somatic cells, allowing the production of NSC in greater numbers [16–18]. These putative NSC are assessed for key NSC properties; self-renewal via neurosphere assay, and multi-lineage differentiation into neurons, astrocytes and oligodendrocytes. Additionally, a number of phenotypic markers, such as nestin, can be used to identify NSC, however there is no available marker to definitively identify NSC [15].

Dental pulp stem cells (DPSC) are a heterogeneous population of multipotent adult stem cells obtained from the dental pulp tissue of molars [19, 20]. These cells display both mesenchymal and neural traits. Similar to mesenchymal stem/stromal cells (MSC), they can differentiate along the adipogenic, chondrogenic and osteogenic lineages [21–23]. Additionally, research has demonstrated that DPSC exhibit neurogenic properties under specific environmental conditions [24–26]. This neural propensity is believed to be attributed to their mesoectodermal origin, with a sub-population of DPSC deriving from the cranial neural crest cells during embryonic development [27–30]. This origin is further reflected in the heterogeneous expression of neural lineage markers, including low-affinity nerve growth factor receptor (also known as p75) that binds neurotrophins involved in neuronal development and function, the intermediate filament nestin implicated in axonal growth, and the intermediate filament glial fibrillary acidic protein [22]. Additionally, DPSC may express some more mature neuronal lineage markers, such as the microtubule element β-III tubulin, and neuronal nuclear antigen that is expressed by nearly all neurons and no glial cells [22, 31]. The DPSC population presents with several advantages for clinical use which include high proliferative and clonogenic potential, easy accessibility through a non-invasive procedure, and can be applied in autologous cell therapy [19, 32].

Whilst DPSC have led to positive neurobehavioural outcomes in stroke models, this has been mediated through paracrine effects [33–35]. DPSC produce factors including chemokine stromal cell-derived factor-1, nerve growth factor, brain-derived neurotrophic factor, glial cell-derived neurotrophic factor, and vascular endothelial growth factor [36]. In order to enhance their neural potential, including their ability to replace neural cells, DPSC would need to be further directed along the neural lineage. As such, we aimed to convert human DPSC into NSC using cellular reprogramming techniques.

To date, there have been limited studies on the reprogramming of DPSC [37–41]. To the best of our knowledge, there are no publications describing the effect of the octamer-binding transcription factor 4 (OCT4) overexpression on DPSC from a neural perspective. The OCT4 transcription factor is associated with pluripotency and is important in the regulation of ESC proliferation and lineage commitment during embryogenesis [42]. This transcription factor is one of multiple factors used for iPSC generation [43, 44], but has also been extensively used in direct reprogramming studies [45–48], where it is thought to act as a plasticity inducer with environmental cues determining the cell lineage outcome [49, 50]. Here, the effect of the OCT4 transcription factor on the neural induction capacity of human DPSC was investigated under defined neural inductive conditions.

## Materials and Methods

### Isolation and Culture of Human DPSC

Human DPSC were isolated as described by Gronthos et al. [19]. Briefly, discarded impacted third molars were collected with informed consent from adult patients undergoing routine extractions, under approved guidelines set by the University of Adelaide (Australia) Human Research Ethics Committee (H-2015-253). Teeth were cleaned and opened to reveal the pulp chamber. The dental pulp tissue was separated from the crown and root, minced and digested in a solution of 3 mg/mL collagenase type I (Life Technologies) and 4 mg/mL dispase (Sigma-Aldrich) for 1–2 h at 37 °C. The solution was centrifuged and the supernatant removed. Cultures were established by seeding the cell pellet into tissue culture-treated flasks in standard DPSC culture medium (α-modification Eagle’s Minimal Essential Medium (Sigma-Aldrich), 10% (v/v) foetal bovine serum (FBS) (CellSera Australia), 100 units/mL penicillin (Gibco), 100 μg/mL streptomycin (Gibco), 2 mM L-glutamine (Gibco), 100 μM L-ascorbate 2-phosphate (Alpha Laboratories), 1 mM Sodium pyruvate (Gibco)), and incubating at 37 °C in a humidified 5% CO_2_ incubator. DPSC between passages 3–6 were used for experiments.

### Cell Proliferation Assay

Cell proliferation was assessed using the water-soluble tetrazolium salt WST-1 premix (Takara Bio). Cells were seeded at 1 × 10^4^ cells/well in triplicate in two 96-well plates in standard DPSC culture medium, for 0-h and 24-h time points. Medium-only controls were included. The 24-h time point plate was incubated, whereas WST-1 premix was added to each well of the 0-h time point plate at a 1:10 dilution for a total volume of 100 µL, and incubated for 4 h. Absorbance was measured at a test wavelength of 490 nm and reference wavelength of 650 nm, using the GloMax® Discover microplate reader (Promega, USA). This was repeated for the 24-h time point plate the following day. Absorbance was corrected by removing background absorbance (medium-only controls). Proliferation rate was defined as the fold-change in absorbance at 24 h compared to the baseline control (0 h).

### Lentiviral Constructs

*POU5F1*, the human OCT4 gene, cDNA and enhanced green fluorescent protein (GFP) marker were sub-cloned, using restriction enzyme digests, from the retroviral vectors pMXs-hOCT3/4 (a gift from Shinya Yamanaka, Addgene plasmid # 17217; http://n2t.net/addgene:17217; RRID:Addgene_17217) [43] and pRUF-iG2-Gateway (Stan Gronthos, The University of Adelaide, Australia) respectively, into a third generation lentiviral vector (a gift from Jialiang Wang, Addgene plasmid # 46970; http://n2t.net/addgene:46970; RRID:Addgene_4697) [51] (Fig. S1). The control empty vector (EV) lacked the transcription factor cDNA. Plasmids were verified by Sanger sequencing (Australian Genome Research facility, Australia). Lentiviral plasmids were cotransfected with psPAX2 (a gift from Didier Trono, Addgene plasmid # 12260; http://n2t.net/addgene:12260; RRID:Addgene_12260) and pCMV-VSV-G (a gift from Bob Weinberg, Addgene plasmid # 8454; http://n2t.net/addgene:8454; RRID:Addgene_8454) [52] packaging plasmids into HEK293T Lenti-X™ Cells (Takara Bio) using Lipofectamine™ 2000 Transfection Reagent (Invitrogen) to initiate production of replication-incompetent, Vesicular Stomatitis Virus-Glycoprotein-coated, recombinant lentiviral particles. Viral supernatants were harvested 48 h after transfection and ultracentrifuged to concentrate the virus.

### Fluorescence-Activated Cell Sorting

Transduced DPSC were harvested and resuspended in 1% (v/v) FBS in phosphate buffered saline (PBS), and underwent fluorescence-activated cell sorting on the BD FACSMelody™ cell sorter using BD FACSChorus™ software (Becton Dickinson, USA) at the Australian Cancer Research Foundation Flow and Laser Scanning Cytometry Facility (South Australian Health and Medical Research Institute, Australia). Events were gated for single events and then for GFP fluorescence based on the negative control. The brighter cells in the positive fraction were sorted for purity.

### Lentiviral Transduction and Neural Reprogramming Culture

Human DPSC were seeded at 1 × 10^5^ cells/well in 6-well plates the day prior to lentiviral transduction, to yield 70–80% confluency a day later. The cells were transduced in antibiotic-free 50:50 mix of standard DPSC culture medium and pre-inducing medium (KnockOut™ Dulbecco’s Modified Eagle Medium (DMEM)/ Ham’s F12 Nutrient Mixture (F12) (Gibco), 20% (v/v) KnockOut™ Serum Replacement (Gibco), 1 mM L-glutamine, 1 × non-essential amino acids (Gibco), 0.1 mM β-mercaptoethanol (BME) (Sigma-Aldrich), 10 ng/mL basic fibroblast growth factor (Prospec)), supplemented with 8 µg/mL polybrene (Sigma-Aldrich). Following 4 h of incubation, the cells were fed with antibiotic-supplemented 50:50 medium and cultured for 3 days. Cells were sorted for GFP, and 1 × 10^5^ cells/well were cultured in 0.2% (w/v) gelatin-coated (Sigma-Aldrich) (30 min at 37 °C) 6-well plates in DPSC medium for 24 h to recover, then 50:50 mix of DPSC medium and pre-inducing medium for 24 h, before being cultured in pre-inducing medium (step 1 of the neural induction (NI) protocol) for 8 days [53]. The medium was then changed to N2B27 medium (DMEM/F12 (Gibco), 1 mM L-glutamine, 2% (v/v) B-27™ supplement (Gibco), 1% (v/v) N-2 supplement (Gibco), 0.1 mM BME) (step 2 of the NI protocol) for 7 days, and then NSC medium (DMEM/F12, 1 mM L-glutamine, 2% (v/v) B-27™ supplement, 1% (v/v) N-2 supplement, 0.1 mM BME, 20 ng/mL basic fibroblast growth factor, 20 ng/mL epidermal growth factor (Prospec)) (step 3 of the NI protocol) for 7 days [53]. Medium was refreshed every 2–3 days.

### Neurosphere Formation Assay

For the generation of neurospheres from reprogrammed DPSC, 1 × 10^5^ cells/well were cultured in suspension in ultra-low attachment 6-well plates (Corning Inc.) in NSC medium for 1 week. For each sample, 3 wells were used, and for each well, micrographs of 3 fields of view (FOV) were taken. Spheres ≥ 100 μm in diameter or maximum length (where not entirely spherical) were counted. This size was determined to be suitable for distinguishing different sphere sizes at 1 week, and prevent cellular necrosis in oversized spheres.

### Neuronal and Glial Differentiation

For the generation of neurons, astrocytes and oligodendrocytes, reprogrammed DPSC were seeded at 1.5 × 10^4^ cells/cm^2^ onto 0.1 mg/mL poly-D-lysine (Sigma-Aldrich) (overnight at room temperature) and 2 µg/mL laminin (Gibco) (overnight at 37 °C) (neurons and oligodendrocytes) or gelatin (astrocytes) coated glass coverslips or tissue-culture treated 6-well plates, and differentiation media were added. For neurons, DMEM (5 mM glucose) (Gibco) supplemented with 2 mM L-glutamine, 2% (v/v) B-27™ supplement, 1% (v/v) N-2 supplement, 1 mM dibutyryl cyclic adenosine monophosphate (Sigma-Aldrich), and 30 ng/mL neurotrophin-3 (Prospec), was added for 4 weeks [25, 54, 55]. For astrocytes, DMEM (Gibco) supplemented with 1% (v/v) FBS, 2 mM L-glutamine, and 1% (v/v) N-2 supplement, was added for 2 weeks (ThermoFisher Scientific). For oligodendrocytes, DMEM/F12 supplemented with 2 mM L-glutamine and 2% (v/v) B-27™ supplement was used, with 10 ng/mL basic fibroblast growth factor, 10 ng/mL platelet-derived growth factor (ImmunoTools) and 10 nM forskolin (Sigma-Aldrich) added for the first 5 days, then 200 nM L-ascorbate 2-phosphate and 30 ng/mL triiodothyronine (Sigma-Aldrich) added for the next 5 days [56]. The media were refreshed every 2–3 days.

### Real-time Quantitative Polymerase Chain Reaction

Total RNA was isolated using TRI Reagent® (Sigma-Aldrich). First-strand cDNA synthesis was performed using SuperScript™ IV Reverse Transcriptase (Invitrogen). Real-time quantitative polymerase chain reaction (RT-qPCR) reactions were performed using PowerUp™ SYBR™ Green Master Mix, using the fast thermal cycling conditions (Applied Biosystems) on a CFX Connect Real-Time PCR Detection System (Bio-Rad Laboratories Inc.). Details for all primers used are provided in the supplementary information (Table S1). All reactions were conducted in triplicate. Relative gene expression analysis was performed using the ΔΔCt method [57], normalised to *β-ACTIN*.

### Western Blot

Cells were lysed in lysis buffer (50 mM Tris–HCl pH 7.5, 150 mM NaCl, 1 mM EDTA, 1 mM EGTA, 50 mM β-glycerolphosphate, 10 mM sodium fluoride, 2.5 mM sodium pyrophosphate, 1% (w/v) sodium deoxycholate, 1% (v/v) NP-40, 0.1% (w/v) sodium dodecyl sulphate, 14.3 mM BME, 2 mM sodium orthovanadate, 1 × Protease Inhibitor Cocktail (Sigma Aldrich)). Total protein concentrations were determined using the Bio-Rad Protein Assay Dye Reagent (Bio-Rad Laboratories Inc.) per the manufacturer’s instructions. Lysates were boiled in sample buffer (0.5 M Tris-HCl pH 6.8, 10% (w/v) sodium dodecyl sulphate, 20% (v/v) glycerol, 0.12% (w/v) Bromophenol Blue) at 100** °C** for 5 min. Following sodium dodecyl sulphate -polyacrylamide gel electrophoresis, proteins were transferred onto nitrocellulose membranes (Bio-Rad Laboratories Inc.). Membranes were blocked with 5% (w/v) skim milk, washed in 0.1% (v/v) TWEEN® 20 (Sigma-Aldrich) in PBS (PBS-T), and incubated overnight at 4 °C with primary antibodies diluted in 5% (w/v) bovine serum albumin in PBS-T. After washing, membranes were incubated with secondary antibodies in PBS-T for 2 h at room temperature. Details of antibodies used can be found in the supplementary information (Table S2). Blots were scanned using the LI-COR Odyssey® CLx Infrared Imaging System, using the LI-COR Image Studio™ acquisition software (LI-COR Biosciences, USA).

### Immunofluorescence Staining

Cells cultured on glass coverslips were fixed in 10% formalin (Sigma-Aldrich), permeabilised with 0.3% (v/v) Triton™ X-100 (Sigma-Aldrich) in PBS for 10 min at room temperature, washed with 0.1% (v/v) TWEEN® 20 in PBS, and blocked in 10% (v/v) normal horse serum in 0.1% (v/v) Triton™ X-100 in PBS for 1 h at room temperature. Primary antibodies (1:250 mouse anti-β-III tubulin (Merck Millipore, MAB1637), 2 µg/mL mouse anti-NF-M (Invitrogen, 130700), 11.6 µg/mL rabbit anti-GFAP (Dako, 0334), 1:250 rabbit anti-PLP (Abcam, ab28486), or mouse and rabbit isotype controls (Mesenchymal Stem Cell Laboratory research group, The University of Adelaide, Australia)) were diluted in blocking solution and cells were incubated at 4 °C overnight. Primary antibodies were detected by incubating with secondary antibodies (2 µg/mL donkey anti-mouse IgG Cyanin3 (Merck Millipore, AP192C) or 3 µg/mL donkey anti-rabbit IgG Cyanin3 (Jackson Immunoresearch, 711165152)) for 2.5 h at 4 °C. Coverslips were mounted on microscope slides with ProLong™ Gold Antifade Mountant with DAPI (4′,6-diamidino-2-phenylindole) (Invitrogen).

### RNA-Sequencing

Total RNA was extracted from cells using the PureLink™ RNA Mini Kit according to the manufacturer’s protocol (Invitrogen), with an on-column DNase I treatment step (New England Biolabs). The RNA concentration was quantified using the NanoDrop™ 2000 spectrophotometer (Thermo Scientific, USA), and the quality and integrity were assessed on an Agilent Bioanalyser RNA Picochip using the Agilent 2100 Bioanalyzer (Agilent Technologies Inc., USA). Library preparation and RNA-sequencing were performed at the SA Genomics Centre (South Australian Health and Medical Research Institute, Australia). Up to 400 ng of total RNA was used to generate barcoded cDNA libraries from poly(A) enriched mRNA, using the Universal Plus™ mRNA-Seq Library Preparation Kit according to the manufacturer’s recommendations (NuGEN Technologies Inc.). Sequencing was performed on the NextSeq 500 with a high output v2 (75 cycle) kit (Illumina, USA), to generate on average 30 million single-end reads of 75 base pairs length per sample.

### Bioinformatics Analysis

Bioinformatic analysis was performed by the South Australian Health and Medical Research Institute Bioinformatics Facility (South Australian Health and Medical Research Institute, Australia). Initial raw read processing was performed using an in-house pipeline developed at the South Australian Health and Medical Research Institute. Quality of raw 75 base pair single-end sequence reads was assessed using FastQC [58] and results aggregated using R/Bioconductor package *ngsReports* [59]. Reads were trimmed for sequence adaptors using *AdapterRemoval* [60] and aligned to the human genome GRCh37.p13 using the transcriptome algorithm STAR [61]. Mapped sequence reads were summarised to the GRCh37 gene intervals using *featureCounts* [62] using gene annotation obtained from Ensembl (https://grch37.ensembl.org) [63]. Gene counts were filtered for low expression counts by removing genes with less than 1 count per million in more than four samples. The filtered data was normalised using the trimmed mean of M-values algorithm [64]. Differential gene expression analysis was performed using R/Bioconductor packages *limma-voom* [65, 66] and *edgeR* [67]. Expression results were displayed in heatmaps using the *Pheatmap* package [68]. Significance was set at a false discovery rate (FDR) < 0.05 and differentially expressed genes (DEGs) were selected using a log_2_ fold change (logFC) >  + 2/-2. Functional enrichment analysis of DEGs was performed using Gene Ontology (GO), Kyoto Encyclopedia of Genes and Genomes (KEGG) database and Molecular Signatures Database (MSigDB) v7.1 [69–72].

### Chicken Embryo Xenotransplantation Assay

Ethics approval (SAM126) was obtained from the South Australian Health and Medical Research Institute (Australia) Animal Ethics Committee. This assay was performed as previously described [24]. Briefly, freshly fertilised White Leghorn chicken eggs (HiChick Breeding Company Pty Ltd, Australia) were incubated in a 37 °C humidified incubator for approximately 40 h to reach embryonic stages 10–12 [73]. Eggs were wiped over with 70% ethanol, an air pocket was created by drawing out some albumin, and a window was cut into the top of the eggshell. Embryos were visualised and staged by injecting non-toxic black Indian ink (10% (v/v) (Windsor and Newton) in freshly prepared Ringer’s solution (7.2 g NaCl, 0.17 g CaCl_2_, 0.37 g KCl, 0.115 g Na_2_HPO_4_, 5 mM HEPES in 1 L ddH_2_O, pH 7.4)) underneath the embryos. The vitelline membrane was removed from around the head of the embryos. The GFP^+^ cells (5 × 10^3^ cells/μL in culture medium with fast green FCF dye (0.1% (w/v)) (Sigma-Aldrich)) were injected using a glass capillary needle attached to a micromanipulator and pressure injector, into the embryos in the region adjacent to the developing hindbrain. A few drops of Ringer’s solution were placed on top of the embryos, the eggs were sealed with tape, and incubated for 48 h. Following the incubation period, embryos were cut out of the egg and placed in ice-cold PBS. Embryos were dissected; the head removed and cut as an open book (from the cranium towards the hindbrain along the ventral side). Tissues were fixed in 1 mL of 4% (w/v) paraformaldehyde (pH 7.4) (Sigma-Aldrich) for either 2 h at room temperature or overnight at 4 °C.

### Whole-mount Chicken Embryo Immunofluorescence Staining

Following fixation, tissue samples were stained as described previously [24]. The following primary antibodies were used: 4 µg/mL goat anti-GFP antibody (Rockland Immunochemicals, 600101215) and 4 µg/mL mouse anti-β-III tubulin clone TUJ1 antibody (BioLegend, 801201). The following secondary antibodies were used: 5 µg/mL donkey anti-goat IgG Alexa Fluor 488 (Jackson ImmunoResearch, 705485147) and 2 µg/mL donkey anti-mouse IgG Cyanin3 (Merck Millipore, AP192C). Samples were stained with DAPI (4′,6-diamidino-2-phenylindole) (Sigma-Aldrich) for 10 min and placed on microscope slides with ProLong™ Gold Antifade Mountant (Life Technologies).

### Imaging Analysis

Bright-field images of cell cultures were acquired with a Nikon Eclipse TS100 inverted light microscope using NIS Elements F3.0 software (Nikon, Japan). Fluorescence images were captured with the Nikon Ni-Eclipse fluorescence microscope using NIS Elements BR4.40 software (Nikon, Japan). ImageJ software (National Institutes of Health, USA) was used to measure the mean fluorescence intensity and sphere diameters. Embryo tissue samples were imaged using the Olympus FV3000 confocal microscope and FV31S-SW software (Olympus, Japan) at Adelaide Microscopy (The University of Adelaide, Australia). Z-stack series (5 µm step size) were taken at 200 × magnification for the relevant channels and analysed using Imaris software version 6.3.1 (Bitplane, Switzerland).

### Statistical Analysis

Statistical analysis was performed using the GraphPad Prism 8 software (GraphPad Software Inc., USA). Data was presented as mean ± SD with the appropriate statistical test and multiple comparisons test performed (two-tailed) (indicated in figure legends). An alpha value of *p* ≤ 0.05 was considered statistically significant.

## Results

### Isolation and Culture of Human DPSC

Culture-expanded human DPSC grew as a monolayer and displayed a typical fibroblast-like, spindle-shaped morphology (Fig. S2a). The cells exhibited a high proliferation rate, tripling in cell density over a 24-h period (2.96 ± 0.58), based on the WST-1 assay (Fig. S2b). Immunophenotypic analysis showed that the DPSC expressed common MSC surface markers, including CD73, CD90 and CD105 (Fig. S2c). Functional studies found that the DPSC underwent multi-lineage differentiation along the osteogenic, chondrogenic and adipogenic lineages (Fig. S2d, e, f).

### Directing Human DPSC along the Neural Lineage

**Fig. 1:**
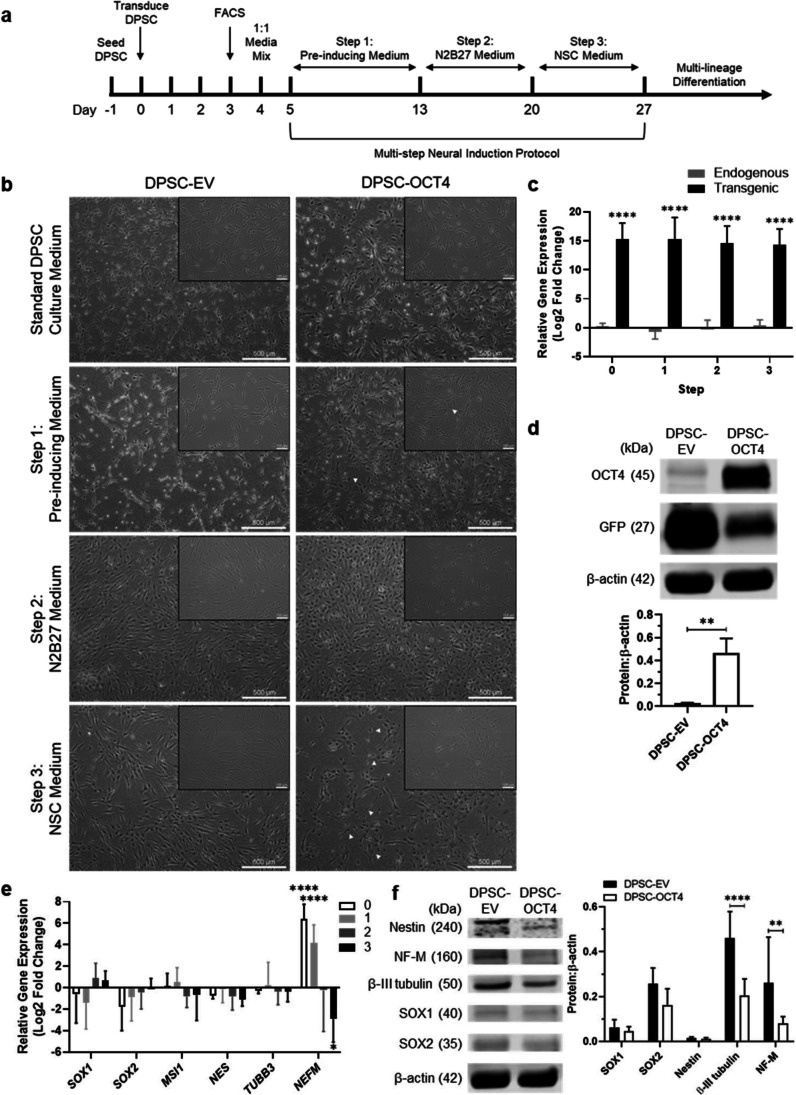
Directing human DPSC along the neural lineage. **a** Neural reprogramming method incorporating lentiviral transduction and a multi-step neural induction protocol. **b** Representative images of transduced cells (DPSC-EV and DPSC-OCT4) cultured in standard DPSC medium, followed by pre-inducing medium (step 1) for 8 days, N2B27 medium (step 2) for 7 days, and NSC medium (step 3) for 7 days. **c** Human OCT4 (*POU5F1*) gene expression levels from endogenous and transgenic sources in DPSC-OCT4 in standard DPSC culture medium (step 0) and after each step of the multi-step neural induction protocol (step 1, 2, 3) relative to DPSC-EV. **d** Human OCT4 protein levels, and GFP levels, in DPSC-EV and DPSC-OCT4 following completion of the multi-step neural induction protocol; protein expression levels were quantified and expressed relative to the loading control β-actin. **e** Effect of OCT4 overexpression on a panel of neural genes; DPSC-OCT4 gene expression levels before (0) and at completion of each step of the multi-step neural induction protocol (1, 2, 3) relative to corresponding DPSC-EV. **f** Protein expression levels in DPSC-EV and DPSC-OCT4 at the completion of the multi-step neural induction protocol; quantified and expressed relative to the loading control β-actin. Scale bar = 500 µm (100 µm, inset). Data represents mean ± SD (*n* = 6). The *P*-values were calculated using a two-way ANOVA with Sidak’s multiple comparisons test (c, f) and Tukey’s multiple comparisons test (e), and a Mann–Whitney test (d). *P*-values: ** ≤ 0.01, **** ≤ 0.0001

Human DPSC were transduced with a lentiviral vector encoding for the human OCT4 protein (*POU5F1*) and a GFP tag (Fig. S1). The OCT4-encoding vector-transduced DPSC (DPSC-OCT4) were sorted based on GFP expression, with a 63.1 ± 8.8 percent transduction efficiency in comparison to 92.3 ± 1.6 percent for the control empty vector (EV)-transduced DPSC (DPSC-EV). Following transduction, there was no significant change in proliferation rate over 24 h, as determined by the WST-1 assay (DPSC, 3.17 ± 0.55; DPSC-EV, 3.23 ± 0.95; DPSC-OCT4, 2.39 ± 0.49). The multi-step neural induction (NI) protocol was subsequently initiated (Fig. 1a).

The DPSC-EV and DPSC-OCT4 cultured in standard growth conditions displayed a similar spindle-shaped morphology to the non-transduced DPSC (Fig. 1b, Fig. S2a). After culturing in pre-inducing medium (step 1 of the NI protocol), both groups exhibited pronounced nuclei, similar to that observed in non-transduced DPSC in this medium, with DPSC-OCT4 displaying larger nuclei and flatter morphology (white arrows) (Fig. 1b, Fig. S3a). DPSC-OCT4 underwent further morphological changes, becoming smaller and rounder in N2B27 medium (step 2 of the NI protocol), and displayed neural progenitor-like protrusions in NSC medium (step 3 of the NI protocol) (white arrows) (Fig. 1b). DPSC-EV resembled non-transduced DPSC at each subsequent step, maintaining an elongated morphology (Fig. 1b, Fig. S3a). Importantly, transgenic *POU5F1* levels were approximately 15-fold greater in DPSC-OCT4 than in DPSC-EV, and remained constant throughout NI, as shown by RT-qPCR (Fig. 1c). Elevated OCT4 protein levels were also detected using Western blot, normalised against β-actin levels (Fig. 1d).

Gene expression changes of several early neural (*SOX1, SOX2, NES*), early neuronal (*NES*), intermediate neuronal (*TUBB3*) and late neuronal (*NEFM*) markers were measured via RT-qPCR throughout NI. Several comparisons between DPSC-EV and DPSC-OCT4 pre- and post-NI demonstrated increased expression levels of neural markers, similar to non-modified DPSC under neural inductive conditions (Fig. S3b, S4b). Significant gradual increases in *SOX1* expression levels were observed in both DPSC-EV and DPSC-OCT4, with DPSC-OCT4 also displaying significant increases in *SOX2* expression levels (Fig. S4b). Of note, the increases in *SOX1* and *SOX2* expression were not significant in non-transduced DPSC cultured using the same NI protocol (Fig. S3b). However, the presence of the lentiviral vector alone appeared to have varied effects on *SOX1* expression (Fig. S4c). Enforced expression of the OCT4 gene in DPSC demonstrated a significant decreased expression level of the neuronal marker *NEFM*, which increased in standard DPSC culture medium but decreased throughout NI in comparison to DPSC-EV (Fig. 1e). *NEFM* in DPSC-EV was comparable to the levels in the non-transduced DPSC in the same conditions (Fig. S3b). Protein expression analysis generally aligned with the gene expression data, where increased expression levels were observed during NI, neuronal markers decreased with OCT4 overexpression, whilst NSC markers showed no significant differences in expression levels between DPSC-EV and DPSC-OCT4 (Fig. 1f, Fig. S3c). Additionally, *NANOG* expression, used as a marker of induced pluripotency, was not observed in DPSC in standard growth conditions and in neural inductive conditions, as well as in DPSC-EV and DPSC-OCT4 post-NI (Fig. S5).

### Self-renewal and multi-lineage Differentiation Potential of Reprogrammed DPSC

**Fig. 2:**
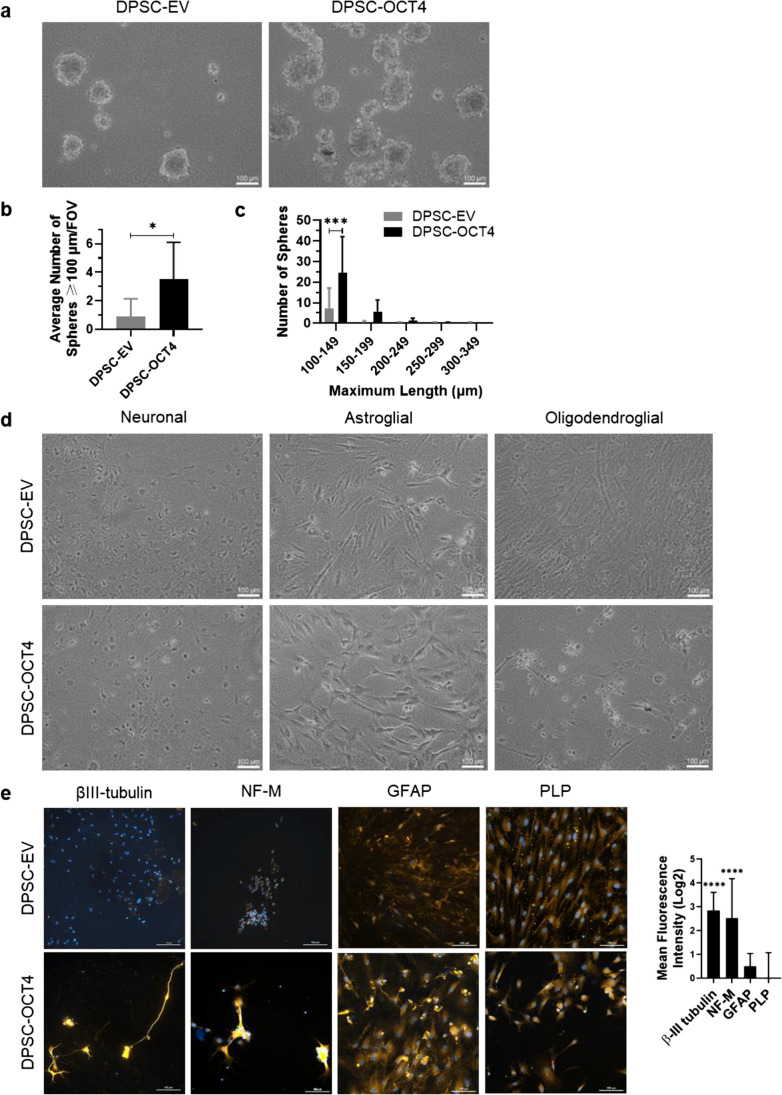
NSC properties of reprogrammed human DPSC. **a** Representative images of sphere formation of DPSC-EV and DPSC-OCT4 post-NI when cultured in NSC medium for 1 week in suspension in ultra-low attachment 6-well plates. **b** For each cell sample, the average number of spheres ≥ 100 µm in maximum diameter or length (where not entirely spherical) were counted in 3 fields of view (FOV) in each of 3 wells. **c** The number of spheres grouped into 50 µm ranges above 100 µm based on their maximum diameter/length. **d** Representative images of terminal differentiation cultures of DPSC-EV and DPSC-OCT4 post-NI, in neuronal (4 weeks), astroglial (2 weeks) and oligodendroglial (10 days) differentiation conditions. **e** Representative images of immunofluorescence assays for protein expression following terminal differentiation; neuronal markers β-III tubulin and neurofilament-medium polypeptide (NF-M), astroglial marker glial fibrillary acidic protein (GFAP), and oligodendroglial marker proteolipid protein (PLP). Mean fluorescence intensity in DPSC-OCT4 was normalised to DPSC-EV. Scale bar = 100 µm. Data represents mean ± SD (*n* = 6). The *P*-values were calculated using a Mann–Whitney test (b) and a two-way ANOVA with Sidak’s multiple comparisons test (c, e). *P*-values: * ≤ 0.05, *** ≤ 0.001, **** ≤ 0.0001

Upon completion of the multi-step NI protocol, the reprogrammed DPSC were assessed for self-renewal, a property of NSC. The cells were cultured in neurosphere-permitting conditions, consisting of NSC medium in low-attachment culture plates. Spheres ≥ 100 µm were counted (Fig. 2a). DPSC-OCT4 post-NI formed a significantly greater number of spheres than DPSC-EV post-NI (3.5 ± 2.6 versus 0.9 ± 1.3 spheres/FOV) (Fig. 2b). There was no significant difference in the average sphere size between DPSC-OCT4 post-NI (131.6 ± 30.3 µm) and DPSC-EV post-NI (128.4 ± 39.6 µm). When separated into 50 µm ranges above the 100 µm threshold, the majority of spheres were in the range of 100 to 150 µm, with significantly more spheres in DPSC-OCT4 post-NI (Fig. 2c). When dissociated and reseeded, secondary spheres greater than 100 µm did not form in either group.

Differentiation potential into neurons, astrocytes and oligodendrocytes was assessed by culturing the reprogrammed DPSC in specific differentiation media. DPSC-OCT4 post-NI demonstrated enhanced neuronal differentiation capacity, displaying rounder cell bodies and extending projections (Fig. 2d), and intense staining for the intermediate and late neuronal markers β-III tubulin and neurofilament-medium polypeptide respectively, whereas the staining in DPSC-EV post-NI was negligible (Fig. 2e). Whilst cells appeared larger after astroglial differentiation as expected of astrocytes (Fig. 2d), no significant difference was observed between DPSC-EV post-NI and DPSC-OCT4 post-NI based on immunofluorescence staining of the astroglial marker glial fibrillary acidic protein (Fig. 2e). Similarly, no difference was observed following oligodendroglial differentiation based on proteolipid protein expression (Fig. 2d, e).

### Transcriptomic Profile of OCT4 Overexpressing, Neural-Induced Human DPSC

**Fig. 3:**
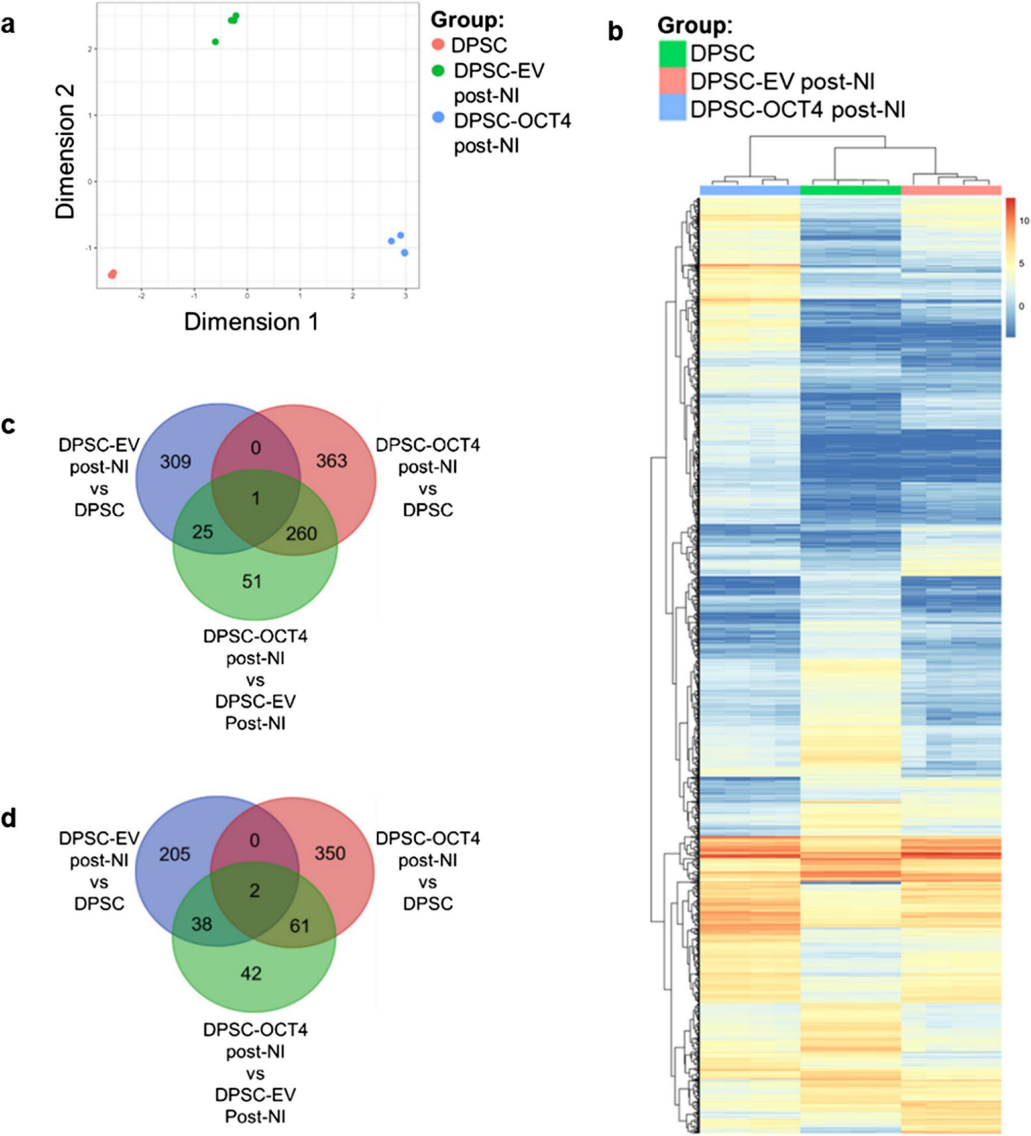
Characterisation of transcriptomic changes in reprogrammed human DPSC. **a** Sample clustering (multi-dimensional scaling plot) of DPSC in standard conditions (DPSC), empty vector-transduced DPSC post neural induction (DPSC-EV post-NI), and OCT4 vector-transduced DPSC post neural induction (DPSC-OCT4 post-NI). **b** Hierarchical clustering heat map of all differentially expressed genes in all samples. **c** Venn diagram illustrating differential gene upregulation between group comparisons. **d** Venn diagram illustrating differential gene downregulation between group comparisons

We next performed RNA-sequencing to characterise the transcriptional profile of DPSC-OCT4 post-NI in comparison to those of DPSC in standard conditions and DPSC-EV post-NI. We applied bioinformatics analysis on our transcriptomic data to identify differentially expressed genes (DEGs) and gene expression patterns involved in neural pathways and processes, based on clustering analysis and gene ontology (GO) functional enrichment analysis. The data showed that DPSC, DPSC-EV post-NI and DPSC-OCT4 post-NI clustered into distinct groups (Fig. 3a). Hierarchical clustering analysis revealed that DPSC-OCT4 post-NI and DPSC-EV post-NI populations separated from each other and from DPSC (Fig. 3b). Notably, DPSC and DPSC-EV post-NI clustered closer together than with DPSC-OCT4 post-NI (Fig. 3b). To confirm the validity of the transcriptomics data, RT-qPCR of selected genes was performed using the same samples used for RNA-sequencing, as well as additional biological samples (Fig. S6a). The RT-qPCR data correlated with the transcriptomics data (Fig. S6b, Table S3). Notably, similar trends were observed between DPSC-OCT4 post-NI and three types of NSC (foetal brain-derived, ESC-derived and iPSC-derived), in particular with expression levels of neuronal genes (Fig. S6c).

We next examined DEGs and their functional annotations. We found 624 upregulated genes and 413 downregulated genes in DPSC-OCT4 post-NI compared to DPSC, and lower number of genes, 337 upregulated and 143 downregulated in DPSC-OCT4 post-NI compared to DPSC-EV post-NI. There were 335 upregulated and 245 downregulated genes in DPSC-EV post-NI compared to DPSC. Analysis of shared and unique DEGs confirmed that the transcriptional changes induced by OCT4 in combination with NI are unique from those induced by NI alone (Fig. 3c, d). Upregulated genes in DPSC-OCT4 post-NI compared to both DPSC and DPSC-EV post-NI were significantly enriched for biological processes, molecular functions and cellular components related to the nervous system, with top GO terms including nervous system development, neurogenesis, generation of neurons, neuron part, synapse, membrane transporters and ion channel activity, whilst downregulated genes were related to the cell cycle (Fig. S7, Tables S4, S5, S6). Meanwhile, upregulated genes in DPSC-EV post-NI in comparison to DPSC were associated with GO terms such as adhesion, migration and motility, whilst downregulated genes were also associated with the cell cycle.

**Fig. 4:**
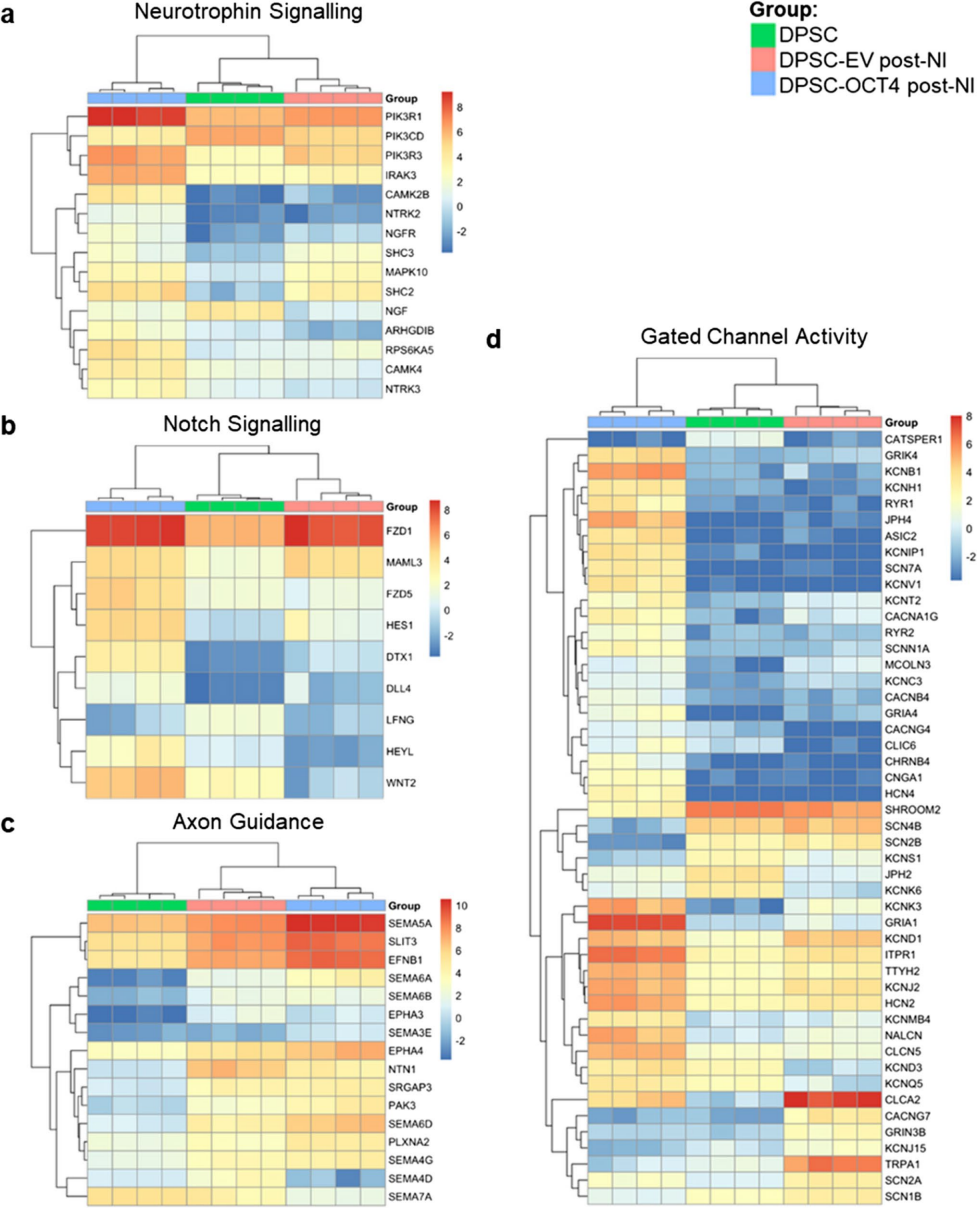
Filtered gene expression profiles for specific gene sets. Genes involved in **a** neurotrophin signalling, **b** notch signalling, **c** axon guidance, and **d** gated channel activity

Notably, enrichment for neurotrophin signalling, which is important for neuronal development, survival and function, was present in DPSC-OCT4 post-NI relative to both DPSC and DPSC-EV post-NI (Fig. 4a) [74]. Notch signalling, important for CNS development and maintenance of neural progenitors, was also enriched for in DPSC-OCT4 post-NI (Fig. 4b) [75]. Additionally, axon guidance, an important stage in neuronal network formation, was enriched in DPSC-EV post-NI and further enriched in DPSC-OCT4 post-NI (Fig. 4c). Furthermore, DPSC-OCT4 post-NI was enriched for genes associated with gated-channel activity, including calcium signalling, and voltage-gated and ligand-gated ion channels involved in signal transduction in neurons (Fig. 4d).

### OCT4 Overexpressing, Neural-Induced Human DPSC Transplanted into a Developmental Avian Model

A developmental avian model was previously adapted to investigate the neuronal differentiation capability of DPSC during a time of active neurogenesis [73]. This *in ovo* model was used to examine the response of the EV- and OCT4-encoding vector-transduced DPSC pre- and post-NI. The GFP^+^ DPSC were injected into chicken embryos in the peripheral tissue directly adjacent to the developing hindbrain during embryonic stages 10 to 12. The location and timing coincide with endogenous cranial neural crest cell activity.

**Fig. 5:**
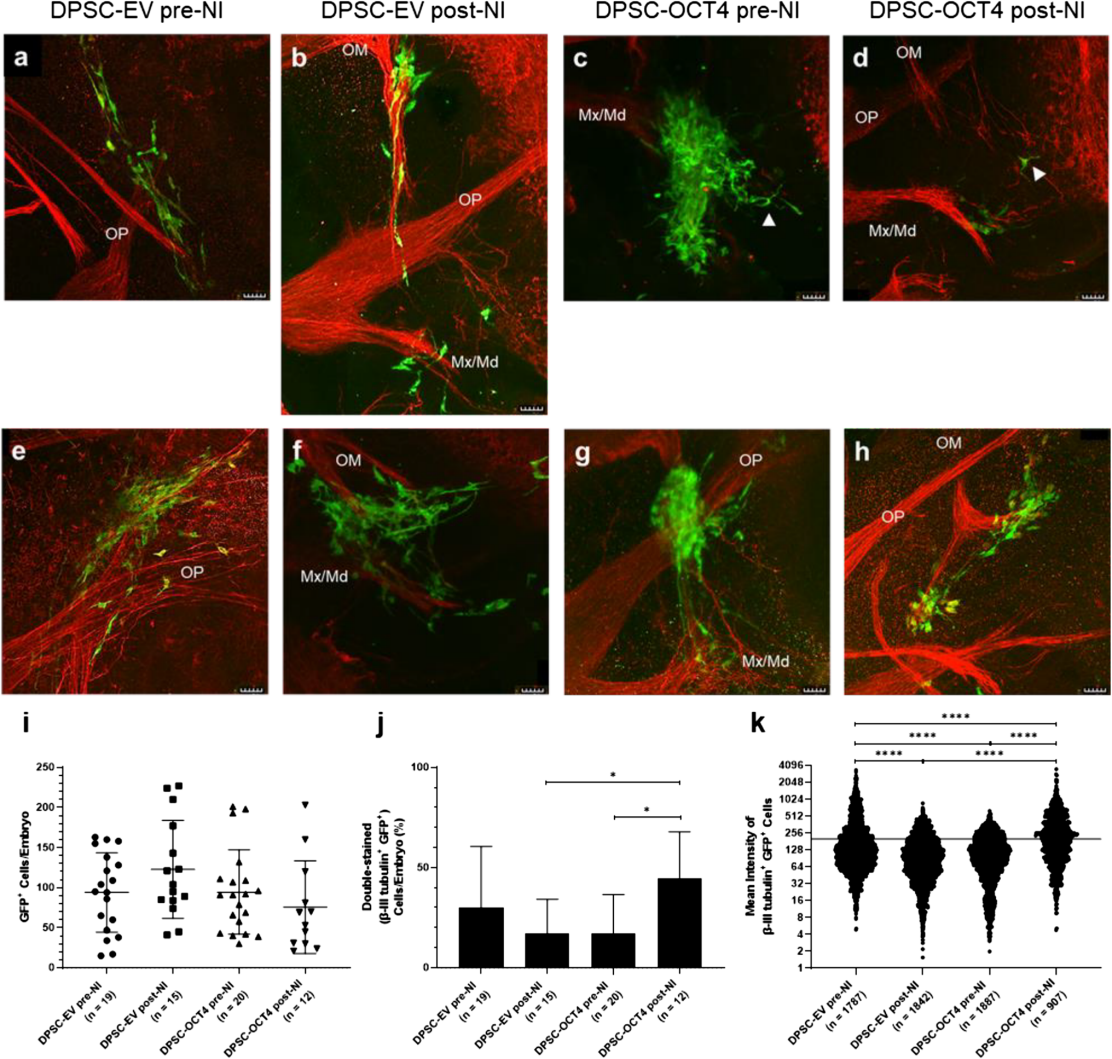
Xenotransplantation assay. Four groups of human DPSC (DPSC-EV pre-NI, DPSC-EV post-NI, DPSC-OCT4 pre-NI, DPSC-OCT4 post-NI) were injected into chicken embryos during a time of active neurogenesis. Tissues were immunostained for GFP (green) and βIII-tubulin (red). Cells within all groups localised with established axonal processes and displayed neuronal-like morphologies **a**–**d**, and induced neuroplasticity as highlighted by the deviation of endogenous axonal processes **e**–**h**. Representative images shown. **i** Number of GFP^+^ DPSC per embryo within each group.** j** Percentage of βIII-tubulin^+^ DPSC from total GFP^+^ DPSC per embryo within each group. **k** Mean intensity of βIII-tubulin staining for every GFP^+^ DPSC within each embryo in every group; line at 200 represents the threshold for βIII-tubulin^+^ staining. Scale bar = 50 µm. Data represents mean ± SD (*n* = embryos (i, j), cells (k)). The *P*-values were calculated using Brown-Forsythe and Welch ANOVA tests with Holm-Sidak’s multiple comparisons test (i), Kruskal-Wallis ANOVA test with Dunn's multiple comparison test (j, k). *P*-values: * ≤ 0.05, **** ≤ 0.0001. Abbreviations: OP = ophthalmic nerve, OM = oculomotor nerve, Mx/Md = maxillary/mandibular nerves

The injected DPSC were examined 48 h post-injection, using antibodies against GFP (green staining). The DPSC were detected in the vicinity of the trigeminal ganglion and the established axonal processes (Fig. 5). All four of the groups, DPSC-EV and DPSC-OCT4 pre- and post-NI, exhibited neuronal-like morphologies; bipolar cells consistent with morphology of trigeminal ganglion sensory neurons were present, as well as multipolar cells (white arrows) (Fig. 5a–d). The DPSC demonstrated the ability to influence the endogenous neuronal axon guidance. Depending on the location of the DPSC aggregates, different patterns of aberrant host axonal branching were observed. The DPSC caused established branches to deviate from their normal developmental path by attracting axonal processes, reshaping and redirecting, and causing branches to bifurcate (Fig. 5e–h).

There was no significant difference in the survival of the injected DPSC between the groups based on the number of GFP^+^ DPSC/embryo (DPSC-EV pre-NI, 94.1 ± 49.6; DPSC-EV post-NI, 122.8 ± 61.2; DPSC-OCT4 pre-NI, 94.4 ± 52.6; DPSC-OCT4 post-NI, 75.6 ± 57.7) (Fig. 5i). Tissue samples were additionally stained for the neuronal marker βIII-tubulin (red staining). There were a greater number of double-stained DPSC/embryo in DPSC-OCT4 post-NI (44.8 ± 22.9) than in the three control groups (DPSC-EV pre-NI, 30.1 ± 30.4; DPSC-EV post-NI, 17.0 ± 17.2; DPSC-OCT4 pre-NI, 17.3 ± 19.3) (Fig. 5j). A further breakdown of this data using the mean fluorescence intensity of the βIII-tubulin^+^ staining to examine the spread of all DPSC, indicated a significantly greater percentage of DPSC-OCT4 post-NI (51%) were double-stained compared to the control groups (DPSC-EV pre-NI, 32%; DPSC-EV post-NI, 18%; DPSC-OCT4 pre-NI, 19%) (Fig. 5k).

## Discussion

The findings of the current study demonstrate that the neural properties of human DPSC can be enhanced through the overexpression of the OCT4 transcription factor and following neural inductive conditions. Human DPSC were induced to overexpress OCT4 and were cultured sequentially in three types of serum-free media to initiate reprogramming and differentiation along the neural lineage. This combination of conditions induced a morphological, transcriptional and physiological response, making these reprogrammed DPSC distinguishable from the control DPSC.

The OCT4 transcription factor is one of the key regulators of pluripotency and its expression level in embryonic development plays an important role in the commitment of pluripotent stem cells to somatic lineages [42]. It is known to interact with other pluripotency-associated transcription factors, functioning by maintaining pluripotency and repressing genes for differentiation [42, 76]. Its expression in ESC and tight control is modulated at multiple levels by many factors and processes, including epigenetic regulation, a complex transcriptional network, and post-translational modifications [42]. Along with three other transcription factors (SOX2, KLF4, c-MYC), OCT4 is used to reprogram somatic cells back into pluripotent cells [43, 44]. On its own, OCT4 has been applied in direct reprogramming studies, and has been shown to increase the plasticity of cells, whereby cells display elevated expression levels of developmental genes associated with multiple cell lineages, whilst specific cell lineage genes are activated in response to subsequent environmental cues, such as neural-specific conditions [45, 46, 48, 49]. Research on better understanding its involvement in the reprogramming process is ongoing.

Here, it was observed that, based on RT-qPCR data, OCT4 induced changes in expression levels of certain genes in DPSC during neural induction. The expression levels of OCT4 were consistent throughout neural induction. The neural conditions themselves stimulated expression of the neural genes, evident in the non-transduced DPSC as well as DPSC-EV and DPSC-OCT4, with DPSC-EV showing similarity to the non-transduced DPSC. However, more significant increases in expression of the early neural markers, *SOX1* and *SOX2*, were observed in DPSC-OCT4 throughout neural induction. Additionally, the expression of the mature neuronal marker *NEFM*, a neuron-specific intermediate filament, decreased throughout neural induction in DPSC-OCT4, whereas it increased in DPSC-EV to similar levels as the non-transduced DPSC. Interestingly, *NEFM* levels were significantly increased in DPSC-OCT4 whilst cultured in standard DPSC conditions. This was not observed with any of the other selected neural genes. This observation may be indicative of the OCT4-induced plasticity, which we anticipate will increase expression of various genes across different lineages, and perhaps is an indicator that these DPSC-OCT4 may be more receptive to neuronal conditions. We examined a panel of neural genes, however increasing the panel to incorporate genes for other cell lineages may highlight if these DPSC-OCT4 are receptive to alternative lineages. Additionally, further analysis could include examination of other mature neuronal markers, such as neuronal nuclei, or other cytoskeletal components to get a better understanding of what processes may be activated. Together, the comparisons of the expression levels of this panel of genes suggest that in the presence of OCT4, DPSC were more receptive to earlier neural programs under these specific neural inductive conditions.

The neural inductive conditions were based on previous reports that established the gradual transition in molecular states of cells undergoing reprogramming towards the neural lineage [48, 49, 53]. The expression levels of *SOX1*, an important transcription factor for neural development, were previously found to gradually increase at each step of the protocol [53]. Similarly, it was observed here that *SOX1* expression increased during neural induction, with further increases observed in transduced DPSC, particularly DPSC-OCT4. Previous research has also demonstrated that the short-term exposure to the pre-inducing medium was necessary to establish the plastic state of fibroblasts and MSC [49, 53]. This may highlight the importance of a well-defined serum-free medium as well as supplementation with the reducing agent β-mercaptoethanol. This reducing agent is often used in stem cell assays to reduce toxic levels of oxygen radicals, as stem cells physiologically reside in areas of tissues with low oxygen levels. This reducing agent is also known to increase the proliferation rate and expression of neural markers during neural reprogramming though the exact mechanism is unknown [77]. The use of this pre-inducing medium in the multi-step neural induction protocol applied to the DPSC in the current study sets this protocol apart from those previously used to neuronally-differentiate DPSC.

The expression of OCT4 in the DPSC resulted in a slightly decreased proliferation rate that was not significantly different to the proliferation rate of the empty vector control cells and the DPSC under standard conditions. Throughout the multi-step neural induction, the rate of cell expansion decreased suggesting that the cells were undergoing differentiation. Despite this, following neural induction sphere formation was observed indicating the presence of potential stem or progenitor cells. With no subsequent secondary sphere formation capacity and limited self-renewal, it is possible that the stem or progenitor cells were few in number or had undergone differentiation. Additionally, neuronal differentiation but not glial differentiation was enhanced in DPSC-OCT4 post-NI. Collectively, these observations suggested the reprogrammed DPSC had been directed along the neural lineage and may be similar to neuronal progenitor cells rather than NSC.

This outcome was further supported by whole-genome transcriptional analysis revealing the activation of neural cell programs in DPSC-OCT4 post-NI. The cells displayed elevated expression levels of genes associated with the CNS and neurogenesis. This was significantly different to the effects of the neural inductive conditions only (DPSC-EV post-NI), which was less enriched for neural terms. This further supports the conditional effect of OCT4, whereby OCT4 enhanced the effects of the neural inductive conditions, which affected genes relevant to neuronal processes. This aligns with the previous assay results, suggesting the induced DPSC are not as immature as NSC but more likely restricted to the neuronal lineage and may be neuronal progenitor-like or immature neuronal-like cells. Moreover, the neural inductive conditions led to a downregulation in genes associated with cell proliferation, supporting our observations, in both DPSC-EV post-NI and DPSC-OCT4 post-NI. This is expected of cells becoming more restricted to a specific lineage and thus differentiating from a stem cell state.

Bioinformatic analysis found the DEGs to be enriched for genes involved in many signalling pathways, most of which have wide-spread roles in cell growth, cell differentiation, gene transcription and protein translation. Importantly, the neurotrophin signalling pathway, critical for the development of neurons [74], was enriched in DPSC-OCT4 post-NI. Similarly, the Notch signalling pathway, which is known to play a role in the CNS, in particular with the maintenance of neural progenitor cells [78], was found to be enriched. The *FZD1* gene, which was most enhanced, encodes for a G-protein coupled receptor that has roles in embryonic development including formation of neural synapses [79]. Another less enhanced gene was *HES1*, which is important in maintaining the NSC pool [75].

Genes for axon guidance and gated channel activity, important for functional neurons, were also upregulated. For example, *SEMA5A*, a gene important for axonal guidance during neural development [80], was highly enriched in DPSC-OCT4 post-NI. Genes involved in excitatory and inhibitory synapses were also detected. This included the *GRIA1* gene encoding for glutamate receptor 1 protein, which is a predominant excitatory neurotransmitter receptor and is also critical for synaptic plasticity [81]. Enrichment was also observed for the calcium signalling pathway, important for neurotransmission, with upregulation of genes for voltage-gated calcium ion channels that are typical of neurons at the early developmental stage. These processes were enriched in DPSC-EV post-NI and further enriched in DPSC-OCT4 post-NI, indicating the combined effect of both OCT4 and the neural conditions, and the possibility of cells existing in different neural states based on treatment conditions.

It was also noted that genes more relevant to neuronal cells, such as *NRCAM*, *MAP2* and *KCNB1*, were detected in the NSC controls. These genes are expected in more mature cells restricted to the neuronal lineage. This highlights the difficulty in classifying NSC and thus establishing a panel of genes that can distinguish neural cells, and others such as our reprogrammed DPSC, at different stages of development and maturity. Future DPSC neural reprogramming studies should dissect this conversion by expanding the transcriptomic analysis to include cells throughout every stage of neural induction, side-by-side with multiple types of NSC.

Further demonstration of the neuronal capacity of these OCT4-overexpressing, neural-induced DPSC was observed in an avian developmental model. By injecting cells into the area adjacent to the developing hindbrain by embryonic stage 12, the cells can be incorporated into the pathways followed by the endogenous cranial neural crest cells and their response examined amongst the well-structured trigeminal ganglion and its branches [24, 82]. The OCT4-overexpressing, neural-induced DPSC exhibited significantly greater expression levels of the intermediate neuronal marker β-III tubulin, used here as a measure of neuronal differentiation potential. Furthermore, the cells displayed neuronal-like morphologies, albeit in all groups. Additional neuronal markers should be examined in future studies, including microtubule-associated protein 2 to detect neurite formation, and further maturation markers such as neuronal nuclei. This model can also be used to examine cell responses following longer periods or injected into other areas of the developing brain. In the current study, cell responses were analysed 48 h post-injection, corresponding to approximately embryonic stage 22 [73]. This time point was previously shown to be suitable for observing potential changes to the morphology, migratory pathway and expression profile of DPSC [24]. Furthermore, with our previous work using this model, we have observed survival of human DPSC up to seven days post-transplantation [24]. This is an important observation as cell stability is a crucial factor that needs to be considered and is often a challenge faced in downstream clinical applications [1].

As expected, the DPSC induced neuroplasticity of the established endogenous axonal processes in the vicinity. Whilst these axonal pathways varied from the physiological patterning, they demonstrated that the DPSC had the capacity to influence axonal growth. From previous research, we know this induced axonal guidance is mediated by the secretion of paracrine factors, including the chemokine stromal cell-derived factor 1 [82]. Further research could examine any differences in this paracrine effect between the groups in this study using an explant model [82]. In combination with other mechanisms, the ability to induce neuroplasticity is beneficial in cell-based therapies. This allows the injected cell population to stimulate homing of endogenous stem and progenitor cells towards the injected cells and the area of injury or disease, and encourage axonal growth in order to re-establish neural connections that have been damaged.

In considering the neurogenic differentiation of DPSC, factors that may influence this process include isolation methods, purification and culture conditions. Here, DPSC were extracted from dental pulp tissue via enzymatic digestion. Whilst previous studies have observed no significant differences on stem cell properties between enzymatic digestion and the explant/outgrowth method, neurogenic differentiation has not been examined sufficiently [83, 84]. The explant method may allow for a specific sub-population of DPSC to migrate out of the tissue. For example, we observed more varied expression of the cell surface glycoprotein CD146, a cell adhesion molecule involved in cell mobility and adhesive interactions [85]. In MSC, CD146 expression is linked with high proliferation and multipotency [86]. This may be a sub-population of interest when the explant method has been applied. Alternatively, purification for a specific marker can be employed prior to neurogenic differentiation. Of particular interest may be the bona fide neural crest stem cell marker p75^+^ sub-population of DPSC, which express higher levels of NSC markers [87]. Neural culture conditions can also have a significant effect on outcomes. By using serum-free media, as this study employed, the formulation contains less variability and contamination with components of animal origin is eliminated.

For DPSC to be applicable for therapeutic use in neurological diseases, standardisation of neural induction is necessary. The potential to use OCT4 in a neural reprogramming method is evident. This transcription factor has demonstrated importance in reprogramming studies, and research into understanding its mechanism is essential. Enhanced neural properties were observed in OCT4*-*overexpressing, neural-induced human DPSC, with data suggesting they resemble neuronal-progenitor cells. This research demonstrates that OCT4 has a distinct role in the neural conversion of the DPSC. Future work should examine the molecular states during neural reprogramming as well as the functional capacity of these cells. An efficient and reliable reprogramming method could provide an alternative source of NSC for use in cell-based therapies for neurological diseases.

### Supplementary Information

Below is the link to the electronic supplementary material.

Supplementary file1 (DOCX 3710 KB)

Supplementary file2 (DOCX 94 KB)

## Data Availability

The transcriptomic (RNA-sequencing) data discussed in this publication has been deposited in NCBI’s Gene Expression Omnibus (Edgar et al*.*, 2002) and is accessible through GEO Series accession number GSE237184. All other data and code are stored at the host institution, and can be made available upon reasonable request.

## Abbreviations

BME: β-Mercaptoethanol
CNS: Central nervous system
DEG: Differentially Expressed Gene
DMEM: Dulbecco’s Modified Eagle Medium
DPSC: Dental pulp stem cells
ESC: Embryonic stem cells
EV: Empty Vector
F12: Ham’s F12 Nutrient Mixture
FBS: Foetal bovine serum
FDR: False Discovery Rate
FOV: Field of View
GFP: Green Fluorescent Protein
GO: Gene Ontology
iPSC: Induced pluripotent stem cells
MSC: mesenchymal stem/stromal cells
NI: Neural Induction
NSC: neural stem cells
OCT4: octamer-binding transcription factor 4
PBS: Phosphate Buffered Saline
RT-qPCR: Real-Time Quantitative Polymerase Chain Reaction
