# Supplementary material for: Effect of Octamer-Binding Transcription Factor 4 Overexpression on the Neural Induction of Human Dental Pulp Stem Cells
Source: Stem Cell Rev Rep. 2024 Feb 5;20(3):797–815. doi: 10.1007/s12015-024-10678-7 (PMC10984899; doi:10.1007/s12015-024-10678-7)
Supplement: Supplementary file 1 — Supplementary file1 (DOCX 3710 KB) [file 12015_2024_10678_MOESM1_ESM.docx]

**Supplementary Information**

Stem Cell Reviews and Reports

Effect of Octamer-binding transcription factor 4 overexpression on the neural induction of human dental pulp stem cells

Maria R. Gancheva*^a,b^, Karlea Kremer^a^, James Breen^a,c^, Agnes Arthur^c^, Anne Hamilton-Bruce^a,d^, Paul Thomas^c,e^, Stan Gronthos^c,e^, Simon Koblar^a^.

^a^Adelaide Medical School, Faculty of Health and Medical Sciences, The University of Adelaide, Adelaide, South Australia, Australia

^b^School of Biological Sciences, Faculty of Science, Engineering and Technology, The University of Adelaide, Adelaide, South Australia, Australia

^c^School of Biomedicine, Faculty of Health and Medical Sciences, The University of Adelaide, Adelaide, South Australia, Australia

^d^Central Adelaide Local Health Network, Adelaide, South Australia, Australia

^e^South Australian Health and Medical Research Institute, Adelaide, South Australia, Australia

^*^Correspondence: Maria R. Gancheva, maria.gancheva@adelaide.edu.au

**Methods**

**Surface Marker Analysis by Flow Cytometry**

For each surface marker to be tested, 1.5 x 10^5^ cells were incubated with antibody solution (1% (v/v) FBS in PBS) and incubated at 4°C for 1 hour. The table below provides the detailed information for the antibodies used. Unstained cells were included as a negative control. Following incubation, samples were washed and were resuspended in fixation buffer (10% formalin solution with 2% (v/v) glucose and 0.02% (v/v) sodium azide). Samples were analysed on the BD FACSCanto II Cell Analyser using the BD FACSDiva 8.0 software (Becton Dickinson, USA), at the Australian Cancer Research Foundation Flow and Laser Scanning Cytometry Facility (SAHMRI, Australia). Single cells were gated (threshold set at 10,000 events) and the negative control cells (no fluorescence) were used to determine the gating for positive staining.

| **Antigen** | **Source** | **Conjugate** | **Dilution** | **Manufacturer** | **Catalogue Number** |
| --- | --- | --- | --- | --- | --- |
| HLA-ABC | Mouse | PE | 1:5 | BD Biosciences | 555553 |
| HLA-DR | Mouse | PE | 1:5 | BD Biosciences | 555812 |
| CD45 | Mouse | PE | 1:10 | BD Biosciences | 555483 |
| CD44 | Mouse | PE | 1:10 | BD Biosciences | 550989 |
| CD73 | Mouse | PE | 1:10 | BD Biosciences | 550257 |
| CD90 | Mouse | PE | 1:400 | BD Biosciences | 555596 |
| CD105 | Mouse | PE | 1:10 | BD Biosciences | 560839 |
| CD146 | Mouse | PE | 1:10 | BD Biosciences | 550315 |

Abbreviations: PE=phycoerythrin.

**Multilineage Differentiation**

**Osteogenic/odontogenic Differentiation and Alizarin Red S Staining**

Human DPSC, at 80-90% confluency, were cultured in osteogenic induction medium (α-modification Eagle’s Minimal Essential Medium, 5% (v/v) FBS, 2 mM L-glutamine, 100 μM L-ascorbate 2-phosphate, 1 mM sodium pyruvate, 100 units/mL penicillin, 100 μg/mL streptomycin, 10 mM HEPES, 100 nM dexamethasone, 2.64 mM potassium dihydrogen phosphate) for 4 weeks, with media changed every 3-4 days. Control DPSC were maintained in standard DPSC culture medium.

To detect mineral deposition, Alizarin Red S staining was performed. Cells were fixed in 10% formalin for 1 hour at room temperature, washed with water, and stained with Alizarin Red S (2% (w/v) aqueous solution, pH 4.1-4.3) (Sigma-Aldrich) overnight at 4°C. After washing, samples were imaged. For quantification, the Alizarin Red S precipitates were solubilised with 10% (v/v) acetic acid and the absorbance measured at 405 nm.

**Chondrogenic Differentiation and DAPI/Safranin-O Staining**

Human DPSC, at 80-90% confluency, were cultured in chondrocyte induction medium (DMEM-high glucose, 2 mM L-glutamine, 100 μM L-ascorbate 2-phosphate, 50 units/mL penicillin, 50 μg/mL streptomycin, 1x insulin-transferrin-sodium selenite, 0.1 mM dexamethasone, 0.125% (w/v) bovine serum albumin, 10 ng/mL transforming growth factor-β3) and control medium (DMEM-high glucose, 5% (v/v) FBS, 2 mM L-glutamine, 100 μM L-ascorbate 2-phosphate, 50 units/mL penicillin, 50 μg/mL streptomycin) for 7 days. Chondrogenic differentiation was analysed using DAPI staining for DNA and Safranin-O staining for sulphated glycosaminoglycans. Following fixation with 4% (w/v) paraformaldehyde for 15 minutes at room temperature, cells were washed and stained with DAPI, and the fluorescence was read at 340 nM excitation/460 nM emission. Cells were washed with 1% (v/v) acetic acid, and 0.1% (w/v) Safranin-O was added. After washing, images were taken. The bound Safranin-O was released by adding 75% ethanol, and the fluorescence was read at 530 nM excitation/570 nM emission. The background reading (blanks) was subtracted, and the mean fluorescence ratio was calculated as normalised sulphated glycosaminoglycans values.

**Adipogenic Differentiation and Oil Red O Staining**

Human DPSC, at 80-90% confluency, were culture in adipogenic induction medium (α-modification Eagle’s Minimal Essential Medium, 10% (v/v) FBS, 2 mM L-glutamine, 100 μM L-ascorbate 2-phosphate, 1 mM sodium pyruvate, 100 units/mL penicillin, 100 μg/mL streptomycin, 60 μM indomethacin, 0.5 mM 3-isobutyl-1- methylxanthine, 0.5 μM hydrocortisone) for 6 weeks with media changes every 3-4 days.

Adipogenic differentiation was evaluated using Oil Red O staining for lipid droplets. Cells were fixed in 10% formalin for 15 minutes at room temperature, and stained with Oil Red O (6 mL of 0.5% (w/v) Oil Red O in isopropanol with 4 mL H_2_O) for at least 2 hours at room temperature. After washing, cells were counterstained with haematoxylin and imaged. For quantification, the Oil Red O dye was extracted using isopropanol, and absorbance was measured at 490 nm.

**Real-time Quantitative PCR**

**Table S1** Primers for RT-qPCR.

| **Gene** | | **Forward Primer** | **Reverse Primer** |
| --- | --- | --- | --- |
| *POU5F1* | Endogenous | gggtttttgggattaagttcttca | gcccccaccctttgtgtt |
|  | Transgene | ggctctcccatgcattcaaactg | aaacgcacaccggccttattcc |
| *SOX1* | | aaaagctgagttagtgcgcccg | ttcagctgactgtgcacgaagc |
| *SOX2* | | caaaaatggccatgcaggtt | agttgggatcgaacaaaagctatt |
| *MSI1* | | tgacaaaaccaccaaccggcac | acatcacctcctttggctgagc |
| *NES* | | agacttccctcagctttcaggac | caggactgggagcaaagatccaag |
| *TUBB3* | | ggcctcttctcacaagtacgtg | ttgtcaggcctgaagagatgtcc |
| *NEFM* | | tggaaaatgagcttcggggcac | gtacgcagcgatttctatatccagagc |
| *ACTB* | | agagctacgagctgcctgac | ttcgtggatgccacaggactc |
| *ALOX15* | | atcactgaaatcgggctgcaagg | acaaagtggcaaacctggtcc |
| *SLC8A2* | | tgctgttgtcttcgttgccctg | aaggaacacgttcaccgcgttg |
| *SLC5A5* | | atctgcaccttctacacggctg | agccacttagcatcaccacgac |
| *PTPRN* | | aagcagagccaaacacctgtgc | tggcgttgatgtaatcgctccg |
| *HES1* | | taaactccccaacccacctct | tctctcccagtattcaagttcct |
| *NRCAM* | | tccaaccatcacccaacagtctc | aatgagtcccattacgggtccag |
| *KCNB1* | | tttggacaaggctgtgctgagc | acacccgcctcaaagttgaacg |
| *SOX11* (FH1 and RH1*) | | aaaatgcatggcaaagtttc | cacaagccactgatattctc |
| *MAP2* | | aagcgctcttctctcccaagac | tgctctgcgaattggctctgac |

*Pre-designed primers (Sigma-Aldrich).

**Western Blot**

**Table S2** Antibodies for Western blots.

| **Name** | **Dilution** | **Company** | **Catalogue Number** |
| --- | --- | --- | --- |
| **Primary Antibodies** | | | |
| Goat anti-GFP | 1:1000 | Rockland Antibodies and Assays | 600101215 |
| Goat anti-OCT4 | 1:400 | R&D Systems | AF1759 |
| Goat anti-SOX1 | 1:200 | R&D Systems | AF3369 |
| Rabbit anti-SOX2 | 1:1000 | Cell Signaling Technology | 3579 |
| Rabbit anti-NANOG | 1:1000 | Abcam | ab109250 |
| Mouse anti-Nestin | 1:1000 | Abcam | ab22035 |
| Mouse anti-β-III tubulin | 1:1000 | Merck Millipore | MAB1637 |
| Mouse anti-NF-M | 1:1000 | Invitrogen | 130700 |
| Mouse anti-β-actin | 1:4000 | Sigma-Aldrich | A2228 |
| **Secondary Antibodies** | | | |
| Rabbit anti-sheep DyLight 680 | 1:20 000 | Invitrogen | SA5-10058 |
| Goat anti-mouse DyLight 680 | 1:20 000 | Invitrogen | 35518 |
| Goat anti-mouse DyLight 800 | 1:20 000 | Invitrogen | SA5-35521 |
| Goat anti-rabbit DyLight 680 | 1:20 000 | Invitrogen | 35568 |
| Goat anti-rabbit DyLight 800 | 1:20 000 | Invitrogen | SA5-35571 |

**NSC Cultures**

Cortical foetal brain-derived NSC (StemPro® Neural Stem Cells, Life Technologies, Product # A15654, Lot # 8901-100) were cultured as per manufacturer’s protocol. Cortical neurospheres derived from iPSC (007 line) and ESC (H9 line) were cultured according to; Denham, M., Dottori, M. (2011). *Neurodegeneration. Methods in Molecular Biology*, and Dottori, M., Pera, M.F. (2008). *Neural Stem Cells. Methods in Molecular Biology*.

**Imaging**

Bright-field colour micrographs were captured on the Zeiss Axiovert 200 microscope using ZEN software (Carl Zeiss, Germany).

**
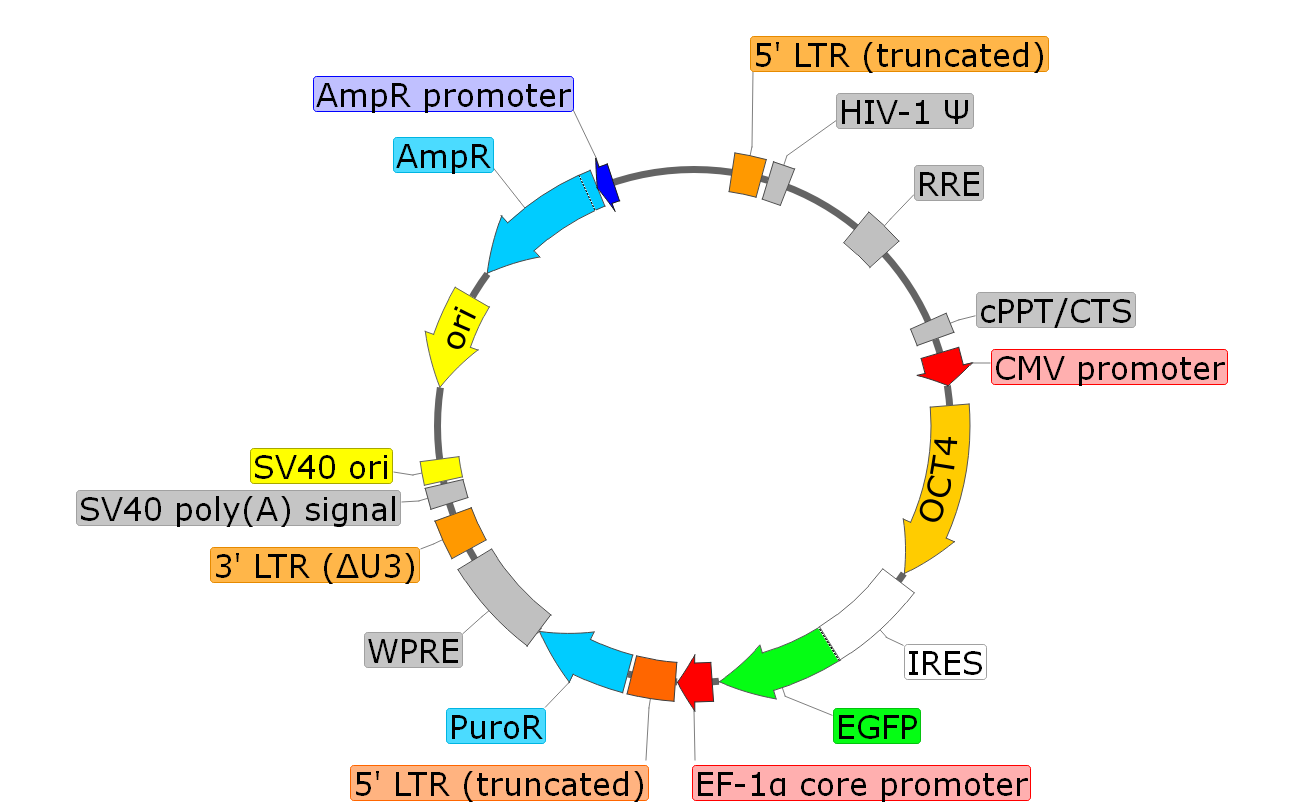
**

**Fig. S1** Lentiviral transfer vector. The 3^rd^ generation HIV-1-derived pCDH lentiviral vector encodes for the transcription factor (human OCT4 gene *POU5F1*) cloned into the multiple cloning site, under control of a hybrid promoter consisting of the truncated HIV-1 5’ long terminal repeat (LTR) fused to the constitutive human cytomegalovirus (CMV) immediate early promoter. The transcription factor coding sequence is followed by an internal ribosome entry site (IRES) and then an enhanced GFP (mammalian codon-optimised) coding sequence. The control empty vector lacks the sequence between the CMV promoter and the IRES. Other important viral elements include the HIV-1 packaging signal psi (Ψ), the HIV-1 rev response element (RRE) that allows for Rev-dependent mRNA export from the nucleus to the cytoplasm, the HIV-1 central polypurine tract and central termination sequence (cPPT/CTS) that is the recognition site for proviral DNA synthesis, the woodchuck hepatitis virus post-transcriptional regulatory element (WPRE) that enhances translation, and the HIV-1 3’ LTR that is self-inactivating due to the deletion of the unique 3’ region (ΔU3). Other components of the plasmid include the core promoter for human elongation factor EF-1α with the truncated human T-cell leukemia virus type 1 5’ LTR, the *pac* gene from Streptomyces alboniger encoding for puromycin N-acetyltransferase that confers puromycin resistance (PuroR), the SV40 polyadenylation (poly(A)) signal for transcription termination, the SV40 origin of replication (ori) for stable propagation of the plasmid in mammalian cells, high-copy-number ColE1/pMB1/pBR322/pUC origin of replication (ori) for maintenance of the plasmid in *E. coli*, the *bla* gene encoding for β-lactamase that confers resistance to ampicillin (AmpR) for plasmid selection in *E. coli*, and the corresponding promoter (AmpR).


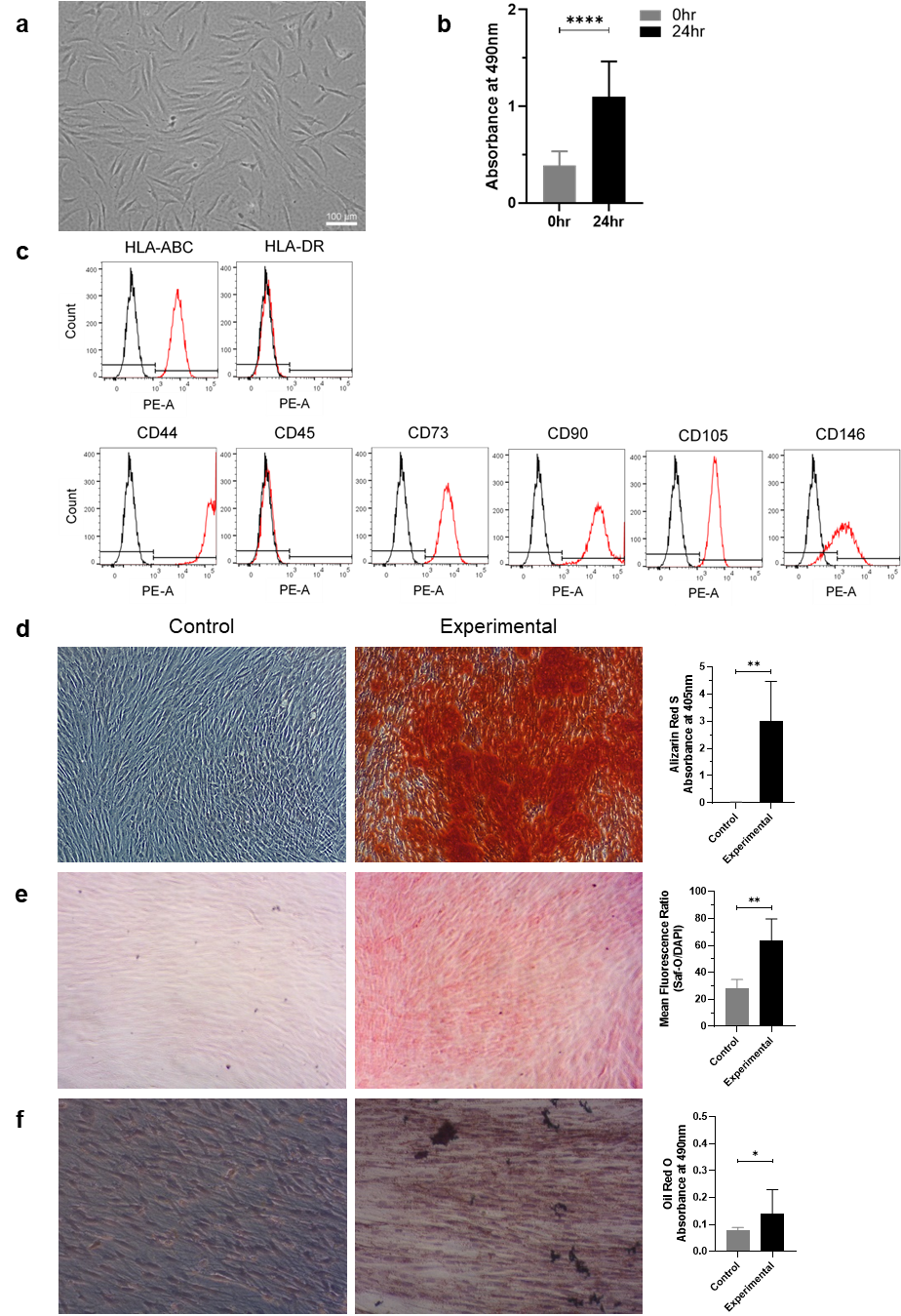


**Fig. S2** Characterisation of human DPSC. **a** DPSC cultured in standard conditions. **b** WST-1 cell proliferation assay over 24 hours. **c** Flow cytometry plots showing the expression of selected surface markers; negative control in black, surface markers in red. **d** Osteogenic/odontogenic differentiation; cells were cultured in control and differentiation media for 4 weeks, Alizarin Red S staining for calcium deposition was performed. **e** Chondrogenic differentiation; cells were cultured in control and differentiation media for 1 week, Safranin-O staining for sulphated glycosaminoglycan expression was performed. **f** Adipogenic differentiation; cells were cultured in control and adipogenic differentiation media for 6 weeks, Oil Red O staining for lipid droplets was performed. Representative images shown. Scale bar = 100 µm (a), 100x (d, e) and 200x (f) magnification. Data represents mean ± SD (n ≥ 5). The *P*-values were calculated using a Mann-Whitney test. *P*-values: *≤0.05, **≤0.01, ****≤0.0001.


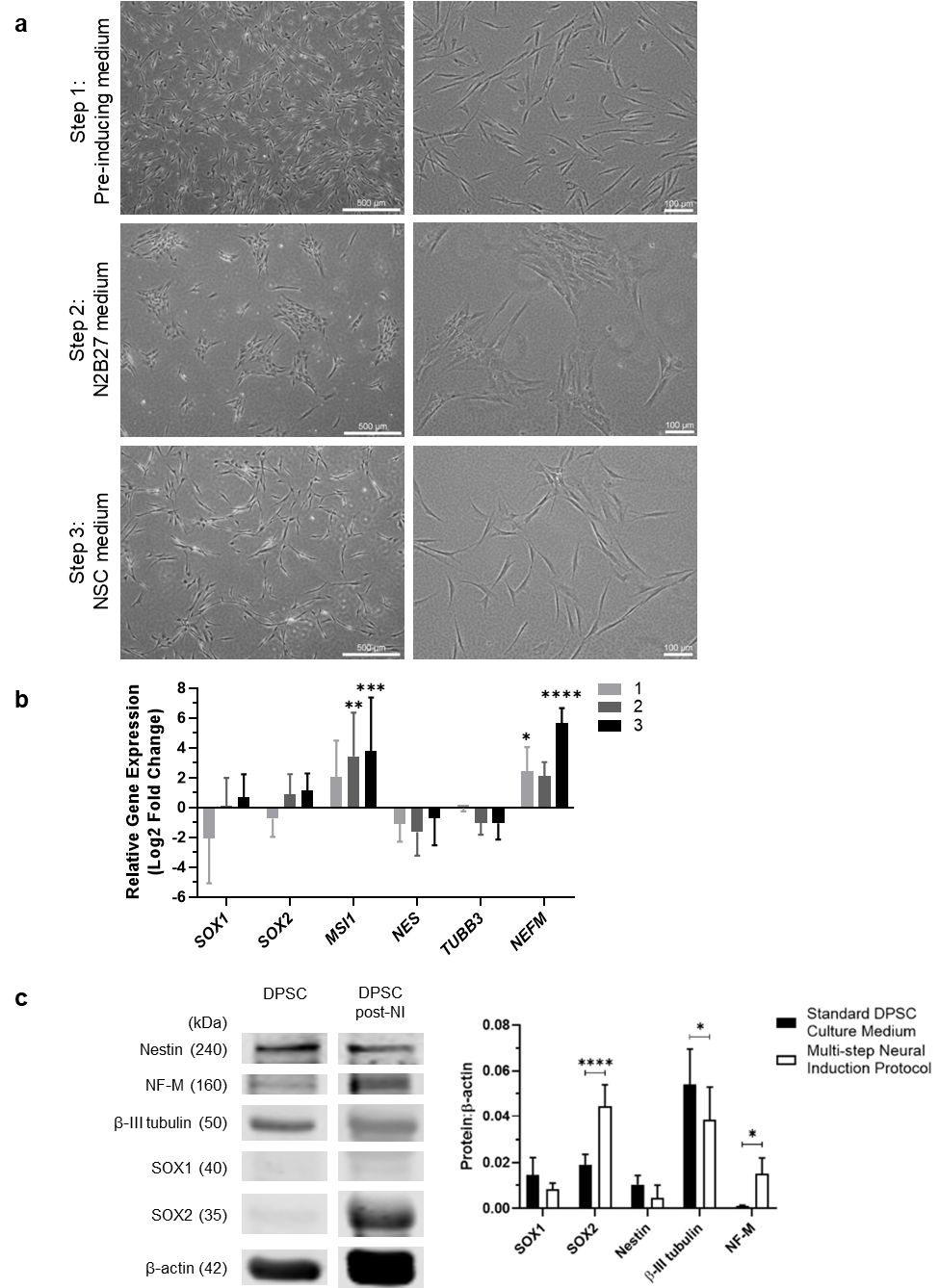


**Fig. S3** Multi-step neural induction protocol applied to human DPSC. **a** Representative image showing the morphology of DPSC after culture in pre-inducing medium (step 1) for 8 days, N2B27 medium (step 2) for 7 days, and NSC medium (step 3) for 7 days. **b** Gene expression levels measured by RT-qPCR at the completion of each step (1, 2, 3), normalised to *ACTB* and expressed relative to DPSC cultured in standard DPSC conditions. **c** Protein levels quantified by Western blot before initiation of the protocol and at completion of the protocol, graphed as a ratio to the loading control β-actin. Scale bar = 100 µm. Data represents mean ± SD (n = 6). The *P*-values were calculated using a two-way ANOVA with Tukey’s multiple comparisons test (b) or Sidak’s multiple comparisons test (c). *P*-values: *≤0.05, **≤0.01, ***≤0.001, ****≤0.0001.


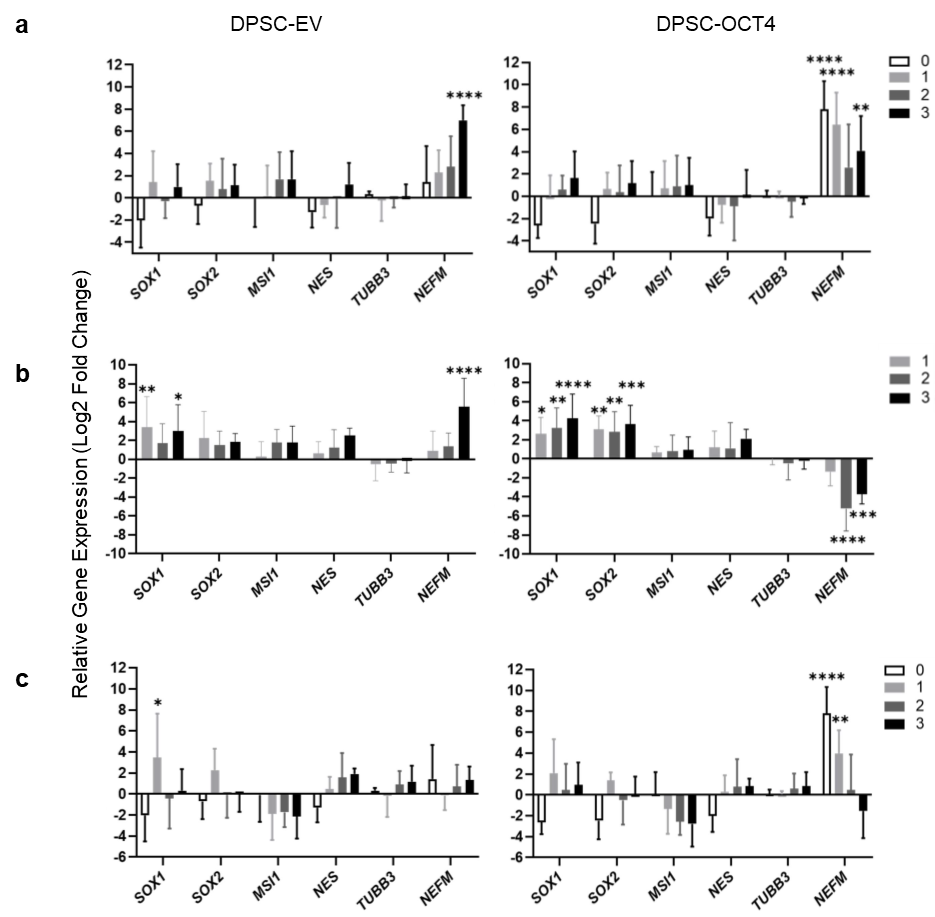


**Fig. S4** Gene expression levels in DPSC-EV and DPSC-OCT4 cultured using the multi-step NI protocol. **a** Changes before and after each step of NI relative to non-transduced DPSC in standard DPSC culture conditions. **b** Changes due to NI; following completion of each step of NI relative to the corresponding transduced sample cultured in standard DPSC conditions. **c** Changes due to presence of lentiviral vector; before and after each step of NI relative to non-transduced DPSC at the corresponding step of NI. Data represents mean ± SD (n = 6). The *P*-values were calculated using a two-way ANOVA with Tukey’s multiple comparisons test. *P*-values: *≤0.05, **≤0.01, ***≤0.001, ****≤0.0001.


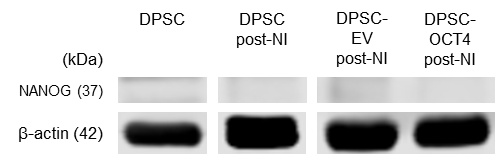


**Fig. S5** NANOG protein detection in human DPSC. DPSC cultured in standard DPSC conditions and using the multi-step NI protocol, and DPSC-EV and DPSC-OCT4 cultured using the multi-step NI protocol. β-actin = loading control (n = 6).


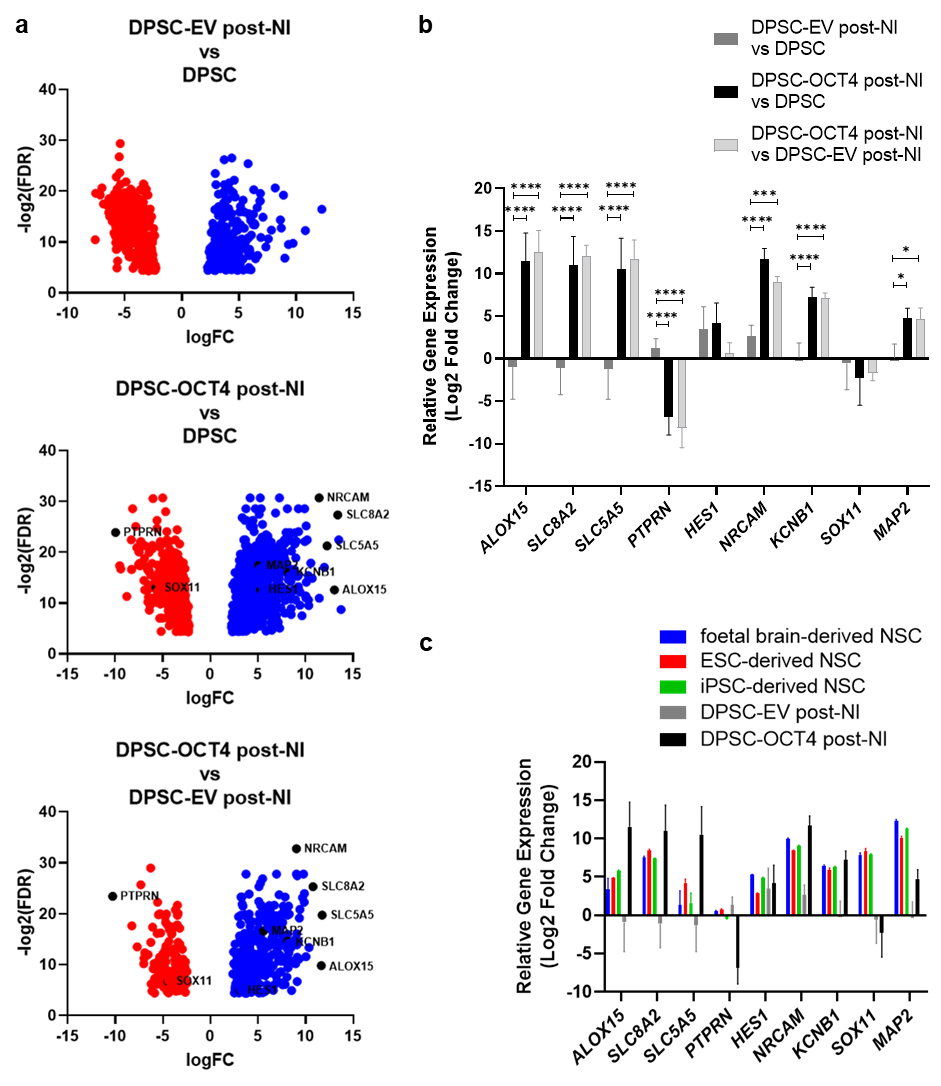


**Fig. S6** Validation of DEGs. **a** Volcano plots of significant DEGs. Significance was set at FDR<0.05. Positive (blue) values are upregulated and negative (red) values are downregulated. Values in black are genes selected for RT-qPCR validation. **b** Validation of selected genes in DPSC (standard DPSC culture conditions), and DPSC-EV and DPSC-OCT4 (post-NI). **c** Gene expression levels compared to NSC (cortical foetal brain-derived NSC (StemPro), ESC-derived NSC (H9 ESC) and iPSC-derived NSC (007)). Gene expression levels were relative to DPSC and normalised to *ACTB*. Data represents mean ± SD (n = 6). The *P*-values were calculated using a two-way ANOVA with Sidak’s multiple comparisons test. *P*-values: *≤0.05, ***≤0.001, ****≤0.0001.


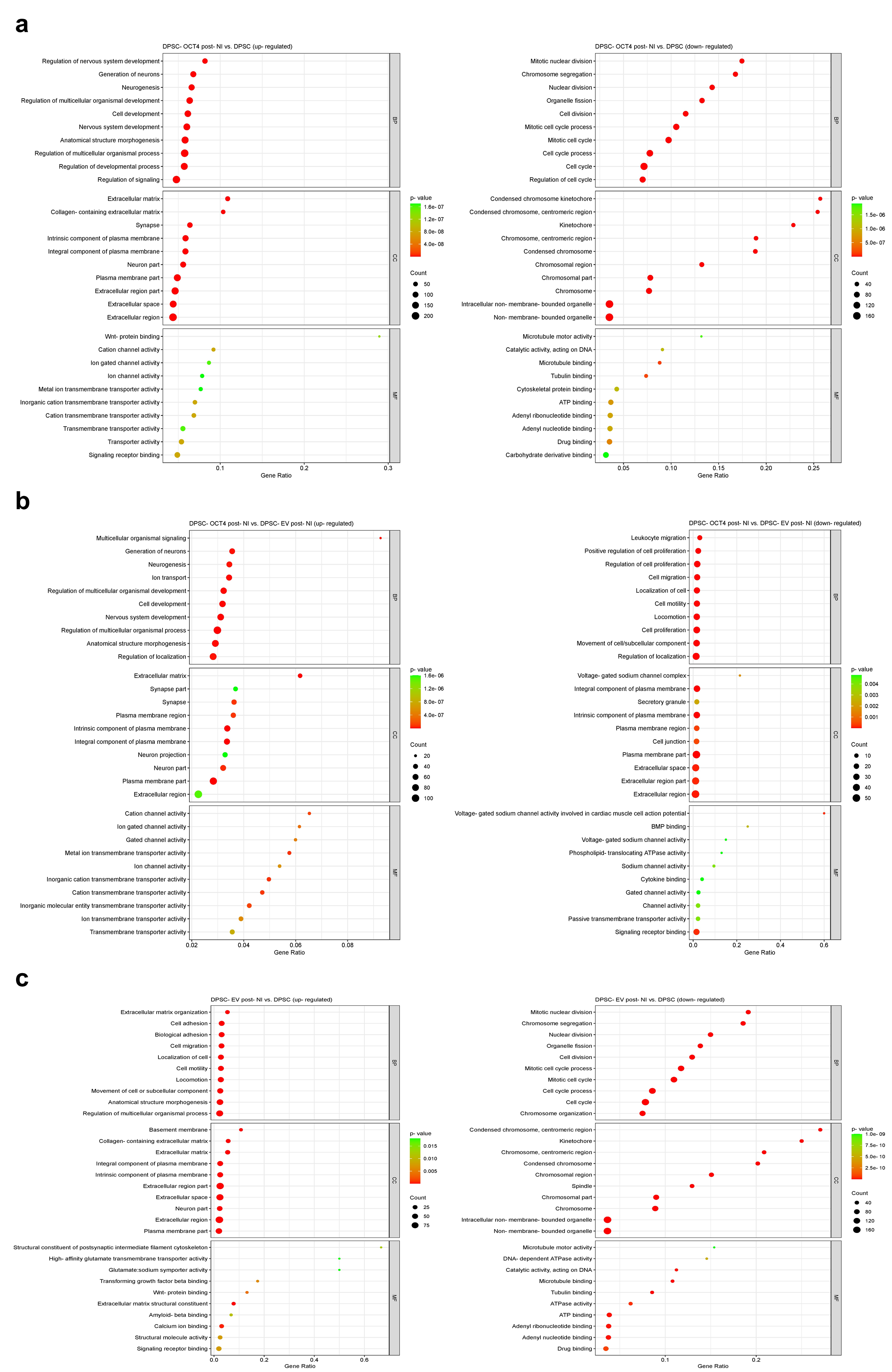


**Fig. S7** Enriched GO terms. GO annotations of DEGs in the three group comparisons; **a** DPSC-OCT4 post-NI vs DPSC, **b** DPSC-OCT4 post-NI vs DPSC-EV post-NI, **c** DPSC-EV post-NI vs DPSC. The top ten most significant upregulated and downregulated terms in the biological processes (BP), cellular components (CC) and molecular functions (MF) categories for each comparison are shown.

**Table S3** Genes selected for RT-qPCR validation of transcriptomics data.

| **Gene** | **Function** | **RNA-seq logFC** | | **Presence in Brain Tissue** |
| --- | --- | --- | --- | --- |
|  |  | **DPSC-OCT4**  **vs. DPSC** | **DPSC-OCT4**  **vs. DPSC-EV** |  |
| *ALOX15* | Lipoxygenase: regulates inflammation and immunity, role in membrane remodelling. | 13.1 | 11.7 | low |
| *SLC8A2* | Solute carrier: sodium-calcium exchange, calcium ion homeostasis in excitable cells, modulates synaptic plasticity. | 13.4 | 10.8 | moderate to high |
| *SLC5A5* | Solute carrier: sodium-iodide transporter in the thyroid gland. | 12.3 | 11.8 | high in pons and medulla |
| *PTPRN* | Protein tyrosine phosphatase: vesicle-mediated secretory processes, required for accumulation of some neurotransmitters. | -10.0 | -10.3 | moderate to high |
| *HES1* | Transcriptional repressor: represses genes that require a bHLH activator, maintains the NSC pool and inhibits neuronal differentiation. | 5.3 | 3.1 | low to moderate |
| *NRCAM* | Neuronal cell adhesion molecule: adhesion in CNS and PNS, directional signalling during axonal cone growth, neurite outgrowth. | 11.4 | 9.0 | moderate to high |
| *KCNB1* | Voltage-gated potassium channel: delayed rectifier in excitable cells, regulates repolarisation. | 8.2 | 8.1 | moderate |
| *SOX11* | Transcriptional activator: role in developing CNS and neurogenesis. | -5.7 | -4.5 | low to moderate |
| *MAP2* | Microtubule-associated protein; microtubule assembly and stabilisation in neurogenesis. | 5.1 | 5.6 | moderate to high |

**Table S4** GO Biological Process annotations of DEGs in the three group (DPSC, DPSC-EV post-NI, DPSC-OCT4 post-NI) comparisons. The top ten upregulated and downregulated terms in the biological processes category are listed with their FDR.

| **DPSC-OCT4 post-NI**  **vs**  **DPSC** | | **DPSC-OCT4 post-NI**  **vs**  **DPSC-EV post-NI** | | **DPSC-EV post-NI**  **vs**  **DPSC** | |
| --- | --- | --- | --- | --- | --- |
| **Upregulated** | | | | | |
| Regulation of multicellular organismal process | 6.30E-28 | Regulation of multicellular organismal process | 9.10E-12 | Regulation of multicellular organismal process | 4.60E-09 |
| Anatomical structure morphogenesis | 1.40E-22 | Nervous system development | 3.80E-09 | Cell adhesion | 4.80E-09 |
| Nervous system development | 5.40E-22 | Regulation of multicellular organismal development | 7.00E-09 | Biological adhesion | 4.80E-09 |
| Regulation of multicellular organismal development | 5.50E-22 | Cell development | 7.00E-09 | Anatomical structure morphogenesis | 6.00E-09 |
| Regulation of developmental process | 1.80E-21 | Ion transport | 9.20E-09 | Cell migration | 1.30E-08 |
| Cell development | 6.40E-21 | Anatomical structure morphogenesis | 9.30E-09 | Locomotion | 1.30E-08 |
| Generation of neurons | 7.70E-19 | Generation of neurons | 1.40E-08 | Cell motility | 8.20E-08 |
| Neurogenesis | 1.00E-18 | Neurogenesis | 1.80E-08 | Localisation of cell | 8.20E-08 |
| Regulation of signalling | 8.10E-18 | Regulation of localisation | 1.80E-08 | Movement of cell or subcellular component | 1.10E-07 |
| Regulation of nervous system development | 8.10E-18 | Multicellular organismal signalling | 1.80E-08 | Extracellular matrix organisation | 1.20E-07 |
| **Downregulated** | | | | | |
| Cell cycle | 2.80E-49 | Regulation of cell proliferation | 9.90E-07 | Cell cycle | 1.10E-74 |
| Mitotic cell cycle | 4.40E-47 | Cell proliferation | 1.70E-06 | Mitotic cell cycle | 3.30E-70 |
| Mitotic cell cycle process | 9.50E-47 | Positive regulation of cell proliferation | 1.70E-06 | Mitotic cell cycle process | 2.50E-67 |
| Cell cycle process | 1.60E-42 | Movement of cell or subcellular component | 6.10E-06 | Cell cycle process | 4.70E-64 |
| Nuclear division | 2.50E-38 | Cell migration | 6.10E-06 | Cell division | 5.10E-53 |
| Organelle fission | 5.70E-37 | Locomotion | 6.10E-06 | Chromosome segregation | 6.30E-49 |
| Cell division | 5.70E-37 | Regulation of localisation | 8.90E-06 | Nuclear division | 1.40E-47 |
| Chromosome segregation | 1.20E-36 | Cell motility | 8.90E-06 | Organelle fission | 4.60E-46 |
| Mitotic nuclear division | 8.10E-35 | Localisation of cell | 8.90E-06 | Mitotic nuclear division | 1.20E-45 |
| Regulation of cell cycle | 1.00E-29 | Leukocyte migration | 2.30E-05 | Chromosome organisation | 2.40E-42 |

**Table S5** GO Cellular Component annotations of DEGs in the three group (DPSC, DPSC-EV post-NI, DPSC-OCT4 post-NI) comparisons. The top ten upregulated and downregulated terms in the cellular components category are listed with their FDR.

| **DPSC-OCT4 post-NI**  **vs**  **DPSC** | | **DPSC-OCT4 post-NI**  **vs**  **DPSC-EV post-NI** | | **DPSC-EV post-NI**  **vs**  **DPSC** | |
| --- | --- | --- | --- | --- | --- |
| **Upregulated** | | | | | |
| Extracellular matrix | 3.10E-19 | Extracellular matrix | 8.50E-11 | Extracellular region part | 8.20E-15 |
| Intrinsic component of plasma membrane | 7.00E-16 | Plasma membrane part | 3.90E-10 | Extracellular region | 8.00E-13 |
| Plasma membrane part | 1.40E-15 | Intrinsic component of plasma membrane | 5.70E-10 | Extracellular space | 1.10E-12 |
| Extracellular region | 1.50E-15 | Integral component of plasma membrane | 1.70E-09 | Extracellular matrix | 3.70E-12 |
| Integral component of plasma membrane | 2.00E-15 | Neuron part | 5.60E-08 | Collagen-containing extracellular matrix | 5.50E-10 |
| Extracellular region part | 5.60E-15 | Synapse | 8.10E-08 | Intrinsic component of plasma membrane | 2.50E-07 |
| Collagen-containing extracellular matrix | 1.10E-13 | Plasma membrane region | 8.90E-08 | Integral component of plasma membrane | 4.90E-07 |
| Synapse | 3.90E-13 | Extracellular region | 1.50E-06 | Basement membrane | 1.20E-06 |
| Neuron part | 6.50E-13 | Neuron projection | 1.60E-06 | Plasma membrane part | 3.50E-06 |
| Extracellular space | 2.20E-11 | Synapse part | 1.60E-06 | Neuron part | 1.90E-05 |
| **Downregulated** | | | | | |
| Chromosome | 1.90E-31 | Plasma membrane part | 5.60E-09 | Chromosome | 1.10E-51 |
| Condensed chromosome | 3.70E-30 | Integral component of plasma membrane | 3.80E-07 | Chromosomal part | 4.50E-46 |
| Chromosomal part | 7.90E-29 | Intrinsic component of plasma membrane | 7.30E-07 | Chromosomal region | 6.00E-40 |
| Chromosomal region | 5.30E-28 | Extracellular region | 6.60E-05 | Condensed chromosome | 6.00E-38 |
| Chromosome, centromeric region | 1.10E-27 | Extracellular space | 9.80E-05 | Intracellular non-membrane-bounded organelle | 4.50E-37 |
| Condensed chromosome, centromeric region | 4.30E-26 | Extracellular region part | 1.50E-04 | Non-membrane-bounded organelle | 4.80E-37 |
| Kinetochore | 1.90E-25 | Plasma membrane region | 3.60E-04 | Chromosome, centromeric region | 2.80E-36 |
| Condensed chromosome kinetochore | 9.00E-24 | Cell junction | 4.40E-04 | Kinetochore | 2.40E-32 |
| Intracellular non-membrane-bounded organelle | 1.40E-21 | Voltage-gated sodium channel complex | 1.70E-03 | Condensed chromosome, centromeric region | 9.30E-32 |
| Non-membrane-bounded organelle | 1.50E-21 | Secretory granule | 2.30E-03 | Spindle | 5.20E-30 |

**Table S6** GO Molecular Function annotations of DEGs in the three group (DPSC, DPSC-EV post-NI, DPSC-OCT4 post-NI) comparisons. The top ten upregulated and downregulated terms in the molecular functions category are listed with their FDR.

| **DPSC-OCT4 post-NI**  **vs**  **DPSC** | | **DPSC-OCT4 post-NI**  **vs**  **DPSC-EV post-NI** | | **DPSC-EV post-NI**  **vs**  **DPSC** | |
| --- | --- | --- | --- | --- | --- |
| **Upregulated** | | | | | |
| Signaling receptor binding | 8.10E-08 | Inorganic cation transmembrane transporter activity | 7.40E-08 | Extracellular matrix structural constituent | 1.20E-06 |
| Transporter activity | 8.10E-08 | Metal ion transmembrane transporter activity | 7.40E-08 | Calcium ion binding | 4.20E-04 |
| Cation channel activity | 8.10E-08 | Cation transmembrane transporter activity | 9.00E-08 | Wnt-protein binding | 3.80E-03 |
| Cation transmembrane transporter activity | 8.10E-08 | Inorganic molecular entity transmembrane transporter activity | 1.30E-07 | Transforming growth factor beta binding | 5.80E-03 |
| Inorganic cation transmembrane transporter activity | 8.10E-08 | Cation channel activity | 1.30E-07 | Signaling receptor binding | 7.50E-03 |
| Wnt-protein binding | 1.30E-07 | Ion gated channel activity | 3.40E-07 | Structural molecule activity | 7.50E-03 |
| Ion gated channel activity | 1.60E-07 | Gated channel activity | 4.50E-07 | Amyloid-beta binding | 1.10E-02 |
| Transmembrane transporter activity | 1.60E-07 | Ion transmembrane transporter activity | 5.30E-07 | Structural constituent of postsynaptic intermediate filament cytoskeleton | 1.20E-02 |
| Ion channel activity | 1.70E-07 | Ion channel activity | 5.90E-07 | High-affinity glutamate transmembrane transporter activity | 1.80E-02 |
| Metal ion transmembrane transporter activity | 1.70E-07 | Transmembrane transporter activity | 8.20E-07 | Glutamate:sodium symporter activity | 1.80E-02 |
| **Downregulated** | | | | | |
| Microtubule binding | 1.30E-07 | Signaling receptor binding | 1.90E-04 | Microtubule binding | 6.00E-14 |
| Tubulin binding | 1.60E-07 | Voltage-gated sodium channel activity involved in cardiac muscle cell action potential | 1.90E-04 | Tubulin binding | 1.10E-12 |
| Drug binding | 5.80E-07 | BMP binding | 2.70E-03 | ATP binding | 8.20E-12 |
| ATP binding | 7.50E-07 | Sodium channel activity | 4.00E-03 | Adenyl ribonucleotide binding | 8.90E-12 |
| Adenyl ribonucleotide binding | 8.70E-07 | Channel activity | 4.00E-03 | Adenyl nucleotide binding | 9.50E-12 |
| Adenyl nucleotide binding | 9.20E-07 | Passive transmembrane transporter activity | 4.00E-03 | Catalytic activity, acting on DNA | 1.10E-11 |
| Cytoskeletal protein binding | 1.10E-06 | Phospholipid-translocating ATPase activity | 4.80E-03 | ATPase activity | 4.10E-11 |
| Catalytic activity, acting on DNA | 1.10E-06 | Voltage-gated sodium channel activity | 4.80E-03 | Drug binding | 8.10E-11 |
| Microtubule motor activity | 1.80E-06 | Cytokine binding | 4.80E-03 | DNA-dependent ATPase activity | 5.00E-10 |
| Carbohydrate derivative binding | 1.90E-06 | Gated channel activity | 4.80E-03 | Microtubule motor activity | 1.00E-09 |
