# Supplementary material for: Effect of Octamer-Binding Transcription Factor 4 Overexpression on the Neural Induction of Human Dental Pulp Stem Cells
Source: Stem Cell Rev Rep. 2024 Feb 5;20(3):797–815. doi: 10.1007/s12015-024-10678-7 (PMC10984899; doi:10.1007/s12015-024-10678-7)
Supplement: Supplementary file 2 — Supplementary file2 (DOCX 94 KB) [file 12015_2024_10678_MOESM2_ESM.docx]

| **Gene** | **logFC** | **FDR** | **Gene** | **logFC** | **FDR** | **Gene** | **logFC** | **FDR** |
| --- | --- | --- | --- | --- | --- | --- | --- | --- |
| DPSC-EV post-NI vs DPSC | | | DPSC-OCT4 post-NI vs DPSC | | | DPSC-OCT4 post-NI vs DPSC-EV post-NI | | |
| CST1 | 12.26 | 1.13E-05 | SPARCL1 | 13.74 | 2.35E-03 | SLC5A5 | 11.75 | 1.15E-06 |
| DRD2 | 10.82 | 2.08E-04 | POU5F1 | 13.51 | 1.75E-07 | ALOX15 | 11.66 | 1.11E-03 |
| CCND2 | 9.73 | 1.16E-03 | SLC8A2 | 13.39 | 5.85E-09 | SLC8A2 | 10.78 | 2.37E-08 |
| CST2 | 9.34 | 1.37E-03 | ALOX15 | 13.05 | 1.62E-04 | KDR | 10.36 | 9.43E-05 |
| APOD | 9.02 | 8.75E-03 | SLC5A5 | 12.27 | 4.00E-07 | LPPR4 | 10.03 | 3.26E-04 |
| ABCA9 | 8.90 | 1.63E-06 | CST1 | 11.97 | 7.30E-06 | PDE2A | 9.88 | 6.16E-08 |
| CLCA2 | 8.70 | 1.44E-04 | APOD | 11.45 | 5.05E-04 | TRPC6 | 9.85 | 1.96E-07 |
| SCG2 | 8.62 | 4.78E-05 | NRCAM | 11.43 | 5.76E-10 | CD180 | 9.78 | 4.28E-09 |
| C3 | 8.17 | 6.04E-07 | JPH4 | 10.71 | 3.37E-05 | POU5F1 | 9.67 | 1.07E-07 |
| SLC7A10 | 7.83 | 1.93E-04 | HPGD | 10.57 | 4.21E-06 | RP11-844P9.2 | 9.40 | 5.25E-07 |
| RANBP3L | 7.56 | 6.41E-04 | MDFI | 10.34 | 1.38E-03 | KCNIP1 | 9.34 | 1.72E-06 |
| MMP1 | 7.36 | 1.90E-03 | CRABP1 | 10.31 | 6.43E-04 | LGI2 | 9.31 | 4.60E-04 |
| CADPS | 7.09 | 3.34E-05 | PTGER3 | 10.30 | 2.35E-05 | HPGD | 9.25 | 4.02E-05 |
| EPHA3 | 6.92 | 5.30E-04 | MEGF10 | 10.18 | 1.75E-07 | TNN | 9.13 | 3.56E-04 |
| IL33 | 6.87 | 1.31E-06 | KDR | 10.10 | 5.69E-05 | MAL | 9.12 | 5.51E-09 |
| BPI | 6.84 | 3.65E-06 | PDE2A | 10.07 | 2.63E-08 | MDFI | 9.11 | 9.77E-03 |
| LRP1B | 6.62 | 6.03E-03 | CCND2 | 10.04 | 4.83E-04 | ADCYAP1R1 | 9.09 | 6.46E-05 |
| AC064875.2 | 6.60 | 9.05E-06 | CST2 | 10.03 | 3.70E-04 | KCNV1 | 9.07 | 1.46E-05 |
| CX3CL1 | 6.45 | 9.57E-03 | ABCA9 | 9.92 | 2.23E-07 | C11orf87 | 9.04 | 4.28E-09 |
| RARRES2 | 6.37 | 8.08E-05 | CD180 | 9.91 | 2.53E-09 | NRCAM | 9.04 | 1.35E-10 |
| CNTNAP3 | 6.37 | 9.54E-05 | GPRIN3 | 9.63 | 5.48E-03 | NELL2 | 8.95 | 8.72E-04 |
| TAC3 | 6.32 | 8.95E-05 | NELL2 | 9.45 | 2.21E-04 | JPH4 | 8.90 | 5.72E-04 |
| INMT | 6.30 | 7.76E-07 | CD38 | 9.41 | 1.31E-03 | TSPAN18 | 8.85 | 3.39E-05 |
| ABCA6 | 6.27 | 2.24E-05 | ADAMTS9-AS1 | 9.27 | 5.73E-08 | GPRIN3 | 8.52 | 3.27E-02 |
| ADH1C | 6.24 | 9.11E-05 | ATP1B2 | 9.26 | 1.04E-04 | SHISA3 | 8.47 | 9.79E-05 |
| NTN1 | 6.20 | 6.80E-05 | TNN | 9.26 | 1.33E-04 | TLR8-AS1 | 8.27 | 1.00E-06 |
| CXCL6 | 6.09 | 3.80E-04 | MAL | 9.25 | 2.53E-09 | ASIC2 | 8.23 | 1.99E-04 |
| ROR2 | 6.05 | 4.36E-02 | ADCYAP1R1 | 9.22 | 2.43E-05 | PTGER3 | 8.20 | 5.23E-05 |
| FYB | 6.03 | 5.07E-05 | ASIC2 | 9.15 | 2.58E-05 | HCN4 | 8.20 | 3.43E-07 |
| FIGF | 5.97 | 5.25E-03 | SCN7A | 9.04 | 2.12E-07 | NCAN | 8.18 | 4.13E-04 |
| RARRES1 | 5.96 | 2.81E-05 | CILP | 8.98 | 2.05E-06 | ELMOD1 | 8.15 | 1.44E-03 |
| PTCHD4 | 5.95 | 3.81E-03 | KCNK3 | 8.97 | 6.41E-04 | KCNB1 | 8.13 | 4.02E-05 |
| PCDH10 | 5.92 | 2.57E-03 | COL11A1 | 8.90 | 3.02E-07 | SCN7A | 8.11 | 1.15E-06 |
| ANGPTL4 | 5.92 | 3.61E-02 | BPI | 8.88 | 6.91E-08 | AGT | 8.10 | 1.56E-05 |
| CACNG7 | 5.91 | 6.20E-03 | KCNV1 | 8.81 | 9.87E-06 | SPARCL1 | 8.09 | 8.86E-04 |
| RXFP1 | 5.87 | 5.31E-04 | TLR8-AS1 | 8.80 | 2.74E-07 | FAM150B | 8.05 | 1.07E-04 |
| SEMA6A | 5.85 | 3.13E-02 | CAMK2B | 8.78 | 1.53E-04 | ATP1A2 | 8.04 | 5.24E-03 |
| FMO2 | 5.84 | 5.83E-03 | PLLP | 8.74 | 1.91E-04 | DOK5 | 8.02 | 7.67E-05 |
| SYTL5 | 5.82 | 2.00E-05 | SLC7A14 | 8.53 | 8.00E-07 | TLL2 | 8.01 | 3.70E-04 |
| COL10A1 | 5.80 | 2.22E-08 | TF | 8.50 | 4.71E-03 | AZGP1 | 7.88 | 4.33E-04 |
| SNCA | 5.80 | 5.74E-04 | WDR86 | 8.49 | 2.03E-03 | PTPRZ1 | 7.84 | 1.43E-02 |
| ANKRD22 | 5.78 | 4.00E-02 | LGI2 | 8.46 | 5.70E-04 | G0S2 | 7.84 | 9.80E-08 |
| CBLN2 | 5.61 | 8.50E-04 | SLC22A3 | 8.45 | 5.05E-05 | KB-1184D12.1 | 7.76 | 5.32E-07 |
| RASL12 | 5.61 | 2.60E-03 | IDO1 | 8.38 | 2.38E-05 | LPPR5 | 7.76 | 4.17E-05 |
| NEFL | 5.57 | 8.54E-04 | PTPRZ1 | 8.37 | 3.68E-03 | PPP2R2B | 7.75 | 1.92E-05 |
| PLEKHS1 | 5.49 | 1.24E-03 | G0S2 | 8.33 | 6.44E-08 | NT5C1A | 7.66 | 3.43E-07 |
| TRPA1 | 5.47 | 1.48E-04 | HCN4 | 8.33 | 1.75E-07 | MAPK4 | 7.65 | 2.32E-05 |
| CYP26B1 | 5.46 | 1.65E-05 | KCNB1 | 8.21 | 1.47E-05 | RYR1 | 7.58 | 1.50E-02 |
| HLA-DRB5 | 5.44 | 9.69E-04 | FAM150B | 8.18 | 3.95E-05 | KCNH1 | 7.58 | 3.12E-06 |
| CCDC102B | 5.38 | 1.03E-03 | FAM65B | 8.18 | 7.78E-06 | CRABP1 | 7.54 | 1.13E-02 |
| PAPPA | 5.38 | 1.47E-03 | NCAN | 8.13 | 1.90E-04 | SPSB4 | 7.46 | 5.17E-06 |
| SYT7 | 5.37 | 4.46E-02 | ADAMTSL3 | 8.09 | 1.07E-07 | LRRC16B | 7.45 | 6.15E-04 |
| RP11-474O21.5 | 5.35 | 3.20E-06 | GRIA1 | 8.09 | 2.53E-09 | SV2B | 7.45 | 1.00E-06 |
| ENTPD1 | 5.32 | 1.04E-02 | RANBP3L | 8.02 | 1.73E-04 | PLLP | 7.42 | 2.56E-03 |
| FLRT3 | 5.31 | 6.67E-04 | AZGP1 | 8.01 | 1.57E-04 | SSTR2 | 7.38 | 5.35E-05 |
| ASPA | 5.19 | 6.74E-04 | TMEFF2 | 7.96 | 6.36E-08 | ADAMTS9-AS1 | 7.37 | 1.27E-06 |
| SLC1A2 | 5.14 | 9.32E-05 | KB-1184D12.1 | 7.89 | 2.39E-07 | TCEAL5 | 7.28 | 2.55E-04 |
| MERTK | 5.13 | 7.59E-06 | CPNE4 | 7.89 | 3.43E-06 | SELP | 7.26 | 2.82E-03 |
| TLR2 | 5.11 | 1.85E-02 | KCNIP1 | 7.89 | 6.44E-06 | MEGF10 | 7.23 | 7.19E-08 |
| FGD4 | 5.08 | 3.14E-02 | LPPR5 | 7.89 | 1.53E-05 | TINAGL1 | 7.22 | 7.47E-06 |
| SOBP | 5.06 | 1.64E-05 | SEMA6A | 7.87 | 6.86E-04 | PRB2 | 7.20 | 4.75E-04 |
| FRAS1 | 5.06 | 1.02E-03 | PKNOX2 | 7.84 | 2.87E-07 | DRD1 | 7.20 | 5.52E-06 |
| PRKG2 | 5.04 | 2.98E-02 | ITGA9 | 7.84 | 3.88E-03 | SLC22A3 | 7.15 | 5.93E-04 |
| COL14A1 | 5.02 | 4.15E-02 | MRO | 7.80 | 2.30E-06 | CTD-2128A3.2 | 7.12 | 1.46E-06 |
| EMID1 | 5.02 | 2.68E-03 | NT5C1A | 7.79 | 1.75E-07 | FGF23 | 7.11 | 9.50E-05 |
| CES3 | 4.98 | 5.07E-03 | SELP | 7.79 | 6.01E-04 | IBSP | 7.04 | 2.43E-06 |
| SHC2 | 4.97 | 5.30E-03 | MAPK4 | 7.78 | 8.91E-06 | ERG | 7.01 | 4.40E-06 |
| GBP1P1 | 4.94 | 7.46E-03 | SCUBE3 | 7.76 | 1.17E-06 | PI15 | 7.01 | 9.68E-03 |
| ABCA8 | 4.91 | 1.78E-04 | HRASLS5 | 7.76 | 2.77E-07 | EFHD1 | 7.00 | 1.08E-05 |
| CTC-378H22.2 | 4.87 | 4.68E-02 | NEFL | 7.75 | 4.71E-06 | NPPC | 6.99 | 4.36E-05 |
| HLA-DRB1 | 4.87 | 1.88E-04 | NPPC | 7.70 | 6.02E-06 | TTBK1 | 6.98 | 7.63E-06 |
| PDE4C | 4.85 | 1.21E-02 | ANO3 | 7.64 | 3.18E-04 | HPCAL4 | 6.98 | 7.63E-05 |
| PREX1 | 4.82 | 5.56E-05 | LPPR4 | 7.64 | 1.78E-04 | GRIA1 | 6.94 | 4.28E-09 |
| COX6B2 | 4.81 | 2.51E-02 | RARRES1 | 7.63 | 7.56E-07 | CNGA1 | 6.93 | 1.34E-05 |
| KIAA1755 | 4.80 | 1.15E-04 | TLL2 | 7.59 | 1.16E-04 | CPA4 | 6.93 | 5.19E-03 |
| ABCA10 | 4.76 | 1.95E-02 | ABCA6 | 7.48 | 1.31E-06 | LRRN1 | 6.91 | 1.39E-05 |
| C1orf145 | 4.75 | 5.68E-03 | ATP1A2 | 7.42 | 1.82E-03 | CALN1 | 6.86 | 1.22E-06 |
| ST8SIA1 | 4.73 | 3.78E-03 | TCEAL5 | 7.41 | 9.18E-05 | MYO7A | 6.86 | 5.66E-05 |
| CXCL10 | 4.67 | 6.35E-03 | TRPC6 | 7.40 | 2.87E-07 | RP11-73G16.1 | 6.86 | 2.10E-04 |
| LRRC17 | 4.66 | 2.10E-06 | FIGF | 7.38 | 1.73E-04 | GATA3 | 6.84 | 7.49E-05 |
| CCK | 4.66 | 2.31E-04 | C8orf4 | 7.38 | 1.15E-04 | CHST8 | 6.72 | 3.00E-07 |
| TMCC3 | 4.65 | 3.69E-02 | CNTN6 | 7.36 | 1.78E-04 | SPP1 | 6.71 | 1.52E-02 |
| CD36 | 4.65 | 1.44E-02 | RP11-844P9.2 | 7.36 | 3.00E-07 | DHRS2 | 6.69 | 5.65E-05 |
| GPX3 | 4.61 | 6.81E-04 | PREX2 | 7.28 | 2.98E-02 | TMEM132C | 6.61 | 2.16E-06 |
| IFI44L | 4.60 | 1.45E-03 | GRIA4 | 7.26 | 9.18E-05 | SERPIND1 | 6.60 | 3.25E-02 |
| TMEM100 | 4.59 | 2.11E-07 | C11orf87 | 7.25 | 5.76E-10 | SLC16A9 | 6.60 | 1.33E-05 |
| DKK2 | 4.58 | 7.92E-04 | CTD-2128A3.2 | 7.25 | 7.00E-07 | ATP1B2 | 6.60 | 1.15E-03 |
| CA9 | 4.53 | 9.40E-03 | FGF23 | 7.24 | 3.46E-05 | WNT2 | 6.51 | 2.47E-04 |
| TXLNB | 4.51 | 1.38E-02 | SPSB4 | 7.19 | 4.21E-06 | CILP | 6.45 | 2.65E-05 |
| FBXO2 | 4.49 | 4.43E-03 | SERPIND1 | 7.14 | 1.35E-02 | DLK1 | 6.41 | 8.01E-06 |
| SPRY1 | 4.48 | 1.16E-06 | CCDC102B | 7.12 | 1.46E-05 | CHRNB4 | 6.36 | 2.98E-03 |
| PAMR1 | 4.47 | 7.00E-05 | PLCH1 | 7.12 | 6.53E-07 | CAMK2B | 6.32 | 1.64E-03 |
| ANKK1 | 4.46 | 1.67E-03 | HPCAL4 | 7.11 | 2.67E-05 | CTC-304I17.5 | 6.30 | 1.00E-06 |
| CLEC2B | 4.39 | 4.18E-04 | SSTR2 | 7.10 | 2.77E-05 | RP11-867G2.6 | 6.26 | 5.65E-07 |
| THBD | 4.38 | 1.36E-02 | DTX1 | 7.10 | 1.58E-06 | SLC10A4 | 6.24 | 3.44E-06 |
| SLC14A1 | 4.38 | 1.02E-08 | LRRN1 | 7.04 | 5.49E-06 | COL11A1 | 6.18 | 3.10E-07 |
| MYH15 | 4.37 | 6.62E-03 | FGL2 | 7.01 | 2.06E-05 | MEGF11 | 6.17 | 7.18E-05 |
| CLGN | 4.36 | 2.07E-05 | SV2B | 7.00 | 1.15E-06 | CDH4 | 6.15 | 2.17E-06 |
| SLC7A14 | 4.36 | 8.28E-03 | RP11-73G16.1 | 6.99 | 7.32E-05 | FNDC5 | 6.14 | 1.85E-06 |
| DGAT2 | 4.35 | 4.32E-02 | LRRC10B | 6.98 | 9.77E-03 | RP11-439C15.4 | 6.12 | 4.56E-04 |
| LPAR3 | 4.33 | 4.14E-04 | SLC15A2 | 6.98 | 1.69E-05 | TPD52 | 6.12 | 6.55E-04 |
| SORL1 | 4.33 | 1.70E-02 | RP11-439C15.4 | 6.96 | 3.95E-05 | SCIN | 6.09 | 7.49E-05 |
| SCUBE3 | 4.32 | 6.04E-03 | PI15 | 6.95 | 4.91E-03 | SLC6A12 | 6.06 | 5.60E-06 |
| RAPGEF4 | 4.32 | 5.70E-03 | SCARA5 | 6.95 | 5.63E-03 | SLC15A2 | 6.06 | 8.30E-05 |
| LINC00519 | 4.30 | 5.50E-03 | PRB2 | 6.93 | 3.27E-04 | FCN3 | 6.01 | 5.32E-07 |
| MMP8 | 4.29 | 2.68E-03 | FAM84B | 6.92 | 7.50E-04 | CLIC6 | 6.00 | 1.80E-02 |
| ITGA10 | 4.24 | 3.08E-05 | HIF3A | 6.90 | 1.81E-04 | HIF3A | 5.98 | 2.27E-03 |
| ADAMTSL3 | 4.24 | 2.84E-03 | RP11-855A2.5 | 6.90 | 2.05E-06 | ISM2 | 5.94 | 1.52E-02 |
| POSTN | 4.24 | 8.55E-07 | CNGA1 | 6.88 | 6.95E-06 | TMEM163 | 5.88 | 3.08E-06 |
| HERC5 | 4.22 | 2.97E-02 | AC064875.2 | 6.87 | 2.54E-06 | CTD-2195M15.1 | 5.85 | 1.14E-06 |
| TENM4 | 4.20 | 6.83E-04 | ADAMTS9 | 6.86 | 4.49E-09 | CACNG4 | 5.84 | 8.93E-05 |
| LAMA3 | 4.20 | 8.55E-07 | CNTNAP3 | 6.85 | 1.82E-05 | CEACAM1 | 5.79 | 8.62E-05 |
| PTGDS | 4.18 | 1.15E-02 | SYTL5 | 6.83 | 1.00E-06 | ITIH3 | 5.77 | 4.40E-03 |
| DHRS3 | 4.15 | 3.02E-07 | CPE | 6.81 | 5.24E-07 | CTD-2306M10.1 | 5.76 | 6.72E-07 |
| HLA-DRA | 4.11 | 3.55E-04 | GATA3 | 6.80 | 2.59E-05 | B3GALT2 | 5.76 | 1.96E-03 |
| COL28A1 | 4.09 | 2.98E-02 | COL21A1 | 6.75 | 1.27E-03 | HEYL | 5.71 | 3.80E-02 |
| TIMP3 | 4.09 | 1.02E-05 | ANKRD22 | 6.75 | 3.68E-03 | TSPAN2 | 5.69 | 5.52E-04 |
| PRCD | 4.06 | 3.20E-02 | ZMAT4 | 6.72 | 1.83E-06 | AADACL2 | 5.66 | 1.00E-06 |
| ISLR | 4.05 | 2.47E-02 | BST2 | 6.59 | 9.35E-04 | AC074289.1 | 5.63 | 2.55E-04 |
| CYGB | 4.04 | 1.02E-05 | EGR2 | 6.57 | 5.61E-04 | ZMAT4 | 5.62 | 4.11E-05 |
| TXNIP | 4.01 | 2.90E-06 | TSPAN11 | 6.57 | 3.46E-06 | MAP2 | 5.61 | 1.01E-05 |
| ARHGAP20 | 4.01 | 5.30E-04 | SFRP2 | 6.56 | 1.57E-04 | BRINP1 | 5.60 | 1.46E-04 |
| AC144831.1 | 3.99 | 2.77E-03 | COL25A1 | 6.54 | 1.59E-04 | C9orf147 | 5.60 | 4.52E-04 |
| CFI | 3.98 | 2.26E-05 | MYO7A | 6.51 | 1.93E-05 | LMO2 | 5.52 | 7.29E-04 |
| HIST1H4H | 3.98 | 8.83E-03 | ELMOD1 | 6.50 | 4.56E-04 | STOX1 | 5.49 | 5.54E-04 |
| PLCH1 | 3.97 | 7.76E-03 | ADAMTSL2 | 6.49 | 4.07E-06 | LANCL3 | 5.49 | 4.45E-02 |
| SAMD3 | 3.94 | 3.20E-02 | ADH1C | 6.49 | 3.01E-05 | AMIGO2 | 5.48 | 8.17E-09 |
| HIST1H2BD | 3.91 | 1.27E-04 | LRP1B | 6.48 | 4.30E-03 | PKNOX2 | 5.44 | 1.67E-06 |
| Z98256.1 | 3.90 | 7.17E-03 | CX3CL1 | 6.48 | 5.19E-03 | WNT11 | 5.44 | 3.90E-03 |
| WTAPP1 | 3.88 | 4.33E-02 | SLC2A5 | 6.47 | 3.99E-02 | ANO3 | 5.40 | 3.62E-03 |
| MIR210HG | 3.86 | 1.67E-04 | RYR1 | 6.44 | 1.99E-02 | LLGL2 | 5.37 | 4.26E-04 |
| CXCL1 | 3.84 | 4.45E-02 | CTC-304I17.5 | 6.43 | 3.91E-07 | FHDC1 | 5.36 | 3.07E-07 |
| PCDHA4 | 3.80 | 1.12E-02 | RP11-867G2.6 | 6.39 | 2.39E-07 | GRIK4 | 5.31 | 7.97E-05 |
| ADAMTS19 | 3.79 | 1.53E-03 | ROR2 | 6.37 | 1.42E-02 | TFAP2E | 5.28 | 3.07E-07 |
| SOX5 | 3.77 | 1.61E-02 | HTR2B | 6.35 | 1.16E-04 | LINGO1 | 5.27 | 2.07E-05 |
| RNF175 | 3.77 | 9.39E-03 | TMEM132C | 6.34 | 2.02E-06 | TSPAN15 | 5.27 | 8.39E-04 |
| SEMA6D | 3.74 | 9.79E-04 | DOK5 | 6.34 | 1.28E-04 | PALD1 | 5.26 | 1.85E-04 |
| FAM65C | 3.72 | 3.93E-07 | RAPGEF4 | 6.32 | 9.00E-06 | HAS3 | 5.26 | 1.44E-08 |
| JUP | 3.71 | 1.29E-08 | RP11-80H8.4 | 6.29 | 8.60E-07 | MMP3 | 5.25 | 6.09E-04 |
| SEPP1 | 3.70 | 1.53E-03 | CADPS | 6.27 | 1.07E-04 | TXK | 5.22 | 1.05E-05 |
| GPC6 | 3.70 | 3.40E-05 | AGT | 6.25 | 6.66E-06 | CRISPLD1 | 5.16 | 2.64E-06 |
| GGT5 | 3.69 | 2.20E-02 | DRD2 | 6.23 | 3.57E-02 | C8orf4 | 5.15 | 2.01E-03 |
| SOD3 | 3.69 | 1.33E-04 | OLFM2 | 6.23 | 1.20E-04 | HRASLS5 | 5.13 | 1.00E-06 |
| SHC3 | 3.69 | 3.01E-02 | CALN1 | 6.20 | 2.52E-06 | LAMP5 | 5.12 | 4.56E-02 |
| QPRT | 3.68 | 1.09E-06 | PDE4C | 6.20 | 2.51E-04 | SPAG17 | 5.11 | 1.30E-02 |
| SPON2 | 3.67 | 6.88E-03 | CHRNB4 | 6.18 | 1.89E-03 | ADAMTS9 | 5.10 | 2.37E-08 |
| LAMA5 | 3.67 | 1.46E-04 | APOE | 6.18 | 1.53E-05 | ADORA1 | 5.09 | 1.30E-02 |
| CLSTN2 | 3.66 | 4.56E-06 | OCA2 | 6.18 | 2.11E-05 | TPD52L1 | 5.08 | 1.81E-07 |
| SVIL | 3.65 | 1.46E-06 | ISM2 | 6.16 | 4.91E-03 | GCH1 | 5.06 | 6.91E-06 |
| KCTD12 | 3.64 | 1.30E-05 | GRIK4 | 6.15 | 1.73E-05 | TMEFF2 | 5.05 | 1.11E-05 |
| PTGES | 3.62 | 3.52E-05 | FCN3 | 6.14 | 2.24E-07 | MYOCD | 5.03 | 1.37E-02 |
| CALB2 | 3.62 | 1.39E-03 | DLK1 | 6.14 | 6.84E-06 | RP11-855A2.5 | 5.03 | 3.17E-04 |
| SFRP4 | 3.61 | 7.46E-03 | POU3F1 | 6.13 | 3.77E-05 | PDE3A | 5.02 | 8.35E-06 |
| IGDCC4 | 3.59 | 5.35E-04 | AC074289.1 | 6.13 | 3.56E-05 | PHOSPHO1 | 5.01 | 3.33E-02 |
| OLFML2A | 3.56 | 2.92E-05 | FMO2 | 6.12 | 1.82E-03 | ITGA9 | 5.01 | 1.37E-02 |
| GRIN3B | 3.55 | 1.28E-02 | KIAA1211 | 6.11 | 1.81E-05 | PCSK9 | 5.01 | 2.69E-06 |
| LPHN1 | 3.55 | 2.28E-04 | SHC2 | 6.10 | 1.91E-04 | CD38 | 4.93 | 3.71E-02 |
| SRGAP3 | 3.54 | 1.75E-02 | PTGDS | 6.06 | 3.24E-05 | FAM65B | 4.91 | 4.11E-05 |
| GDF15 | 3.53 | 8.37E-04 | FGD4 | 6.05 | 2.38E-03 | CH25H | 4.91 | 1.49E-03 |
| TMEM158 | 3.52 | 1.14E-04 | ERG | 6.04 | 1.48E-05 | RP11-143K11.1 | 4.90 | 4.64E-04 |
| INA | 3.51 | 1.99E-02 | CFHR1 | 6.04 | 1.79E-05 | OCA2 | 4.89 | 1.15E-03 |
| HIST1H2AC | 3.48 | 4.50E-04 | FHDC1 | 6.04 | 1.08E-07 | AFF2 | 4.88 | 1.76E-03 |
| NPTX1 | 3.48 | 1.55E-03 | EXPH5 | 6.03 | 1.80E-04 | CPNE4 | 4.87 | 9.00E-04 |
| PPL | 3.48 | 7.87E-03 | ART3 | 6.03 | 3.34E-04 | NRGN | 4.85 | 4.60E-03 |
| FAM43A | 3.47 | 8.31E-05 | WNT11 | 6.02 | 1.03E-03 | GATA2 | 4.84 | 2.39E-02 |
| ZBTB46 | 3.46 | 7.15E-04 | TMEM163 | 6.01 | 1.23E-06 | PYY2 | 4.82 | 5.33E-03 |
| CILP2 | 3.45 | 3.20E-03 | CTD-2195M15.1 | 5.98 | 4.69E-07 | ADAMTSL2 | 4.78 | 5.99E-04 |
| WNT16 | 3.45 | 1.27E-02 | UPB1 | 5.98 | 4.17E-05 | CFHR1 | 4.75 | 1.13E-03 |
| ITGB3 | 3.45 | 1.12E-03 | HEY1 | 5.97 | 8.89E-05 | ADAMTS5 | 4.68 | 6.35E-03 |
| B4GALNT4 | 3.43 | 6.61E-04 | DLL4 | 5.95 | 2.06E-03 | KBTBD11 | 4.68 | 1.64E-05 |
| JAM2 | 3.43 | 1.35E-03 | ABCA13 | 5.95 | 3.36E-06 | NTRK2 | 4.67 | 8.61E-03 |
| PLEKHA6 | 3.41 | 1.21E-05 | TINAGL1 | 5.91 | 2.50E-06 | COL4A1 | 4.60 | 3.44E-03 |
| AKAP6 | 3.39 | 2.53E-04 | KCNH1 | 5.90 | 4.21E-06 | ART3 | 4.59 | 2.06E-02 |
| C20orf195 | 3.38 | 1.61E-02 | CTD-2306M10.1 | 5.89 | 2.74E-07 | CSMD2 | 4.59 | 7.29E-03 |
| RHOJ | 3.36 | 3.78E-02 | PPFIA2 | 5.87 | 2.11E-05 | PCDH1 | 4.57 | 2.22E-04 |
| BFSP1 | 3.35 | 3.36E-04 | PCSK5 | 5.86 | 8.77E-08 | FAM19A5 | 4.54 | 1.58E-02 |
| ACKR4 | 3.33 | 1.66E-02 | EDNRA | 5.85 | 4.51E-02 | SUSD4 | 4.52 | 6.59E-04 |
| SLC1A3 | 3.33 | 1.51E-04 | OGN | 5.85 | 2.20E-05 | DAPK1 | 4.52 | 1.56E-05 |
| HIST1H3E | 3.30 | 1.70E-02 | FAM196B | 5.83 | 1.21E-05 | MRO | 4.52 | 1.04E-03 |
| CNIH3 | 3.27 | 9.41E-03 | FNDC5 | 5.83 | 8.62E-07 | HMCN1 | 4.50 | 2.97E-05 |
| SH3BGRL2 | 3.26 | 3.68E-03 | EFHD1 | 5.81 | 7.30E-06 | HSD17B2 | 4.48 | 1.35E-02 |
| WDR63 | 3.25 | 5.72E-04 | SLC6A12 | 5.79 | 5.42E-06 | KCND3 | 4.48 | 7.25E-03 |
| PCDHGB1 | 3.24 | 6.57E-03 | AADACL2 | 5.79 | 3.91E-07 | LINC00402 | 4.45 | 3.36E-02 |
| ST6GAL1 | 3.24 | 2.36E-03 | CACNA1G | 5.76 | 3.62E-03 | CPE | 4.39 | 2.81E-06 |
| RNF157 | 3.21 | 1.42E-06 | SPAG17 | 5.76 | 2.03E-03 | VANGL2 | 4.38 | 1.11E-03 |
| SNED1 | 3.21 | 2.66E-05 | PPP1R3G | 5.75 | 3.24E-05 | POU3F1 | 4.35 | 2.27E-03 |
| CFH | 3.21 | 7.98E-06 | MEGF11 | 5.72 | 8.89E-05 | PPFIA2 | 4.35 | 3.57E-04 |
| FBLN1 | 3.18 | 3.56E-02 | KIAA1755 | 5.71 | 5.49E-06 | SFRP2 | 4.33 | 1.77E-02 |
| LRRN4CL | 3.17 | 1.95E-03 | EPHA3 | 5.71 | 3.64E-03 | EXOC3L2 | 4.31 | 2.50E-02 |
| BMP2 | 3.17 | 4.31E-04 | SLC26A7 | 5.67 | 2.36E-05 | PLCL1 | 4.31 | 2.41E-03 |
| ITGA2 | 3.14 | 2.91E-03 | PACSIN1 | 5.63 | 5.64E-04 | TFPI2 | 4.29 | 1.56E-05 |
| DUSP6 | 3.13 | 3.11E-03 | C9orf147 | 5.61 | 1.57E-04 | SALL4 | 4.29 | 6.98E-03 |
| ETV1 | 3.08 | 5.24E-03 | FLRT3 | 5.61 | 1.71E-04 | CCDC88C | 4.28 | 4.25E-02 |
| C1QTNF1 | 3.08 | 1.06E-02 | PLCL1 | 5.60 | 2.28E-04 | COL4A4 | 4.28 | 7.29E-03 |
| TMEM35 | 3.08 | 4.52E-04 | SEMA6D | 5.58 | 9.16E-07 | AC108142.1 | 4.27 | 4.68E-04 |
| SCN2A | 3.07 | 1.92E-03 | MYO3B | 5.57 | 1.47E-03 | ARHGDIB | 4.26 | 1.71E-02 |
| PCDHGA4 | 3.06 | 2.35E-04 | TMEM200C | 5.56 | 6.99E-03 | AC068499.10 | 4.23 | 1.20E-03 |
| THRB | 3.06 | 1.61E-02 | CEACAM1 | 5.52 | 7.40E-05 | RP11-446H18.5 | 4.19 | 1.80E-02 |
| RP11-696N14.1 | 3.06 | 2.65E-02 | SLC10A4 | 5.52 | 1.84E-06 | TMTC2 | 4.18 | 6.44E-05 |
| CYFIP2 | 3.04 | 5.36E-04 | EXOC3L2 | 5.51 | 5.23E-04 | SLC7A14 | 4.17 | 5.49E-06 |
| COL18A1 | 3.03 | 4.22E-03 | HIST1H4H | 5.50 | 3.33E-05 | PSG1 | 4.16 | 1.49E-02 |
| MAPK10 | 2.99 | 5.15E-03 | NTRK2 | 5.49 | 6.80E-04 | C5AR1 | 4.14 | 1.17E-04 |
| SFRP1 | 2.99 | 3.93E-07 | AFF2 | 5.49 | 4.17E-04 | BMP3 | 4.12 | 2.94E-02 |
| NYAP1 | 2.96 | 2.35E-02 | SPRY1 | 5.48 | 2.14E-08 | ACBD7 | 4.12 | 2.05E-02 |
| WLS | 2.96 | 1.37E-03 | RP11-446H18.5 | 5.48 | 8.49E-04 | RTN4RL1 | 4.11 | 3.26E-02 |
| MFAP4 | 2.94 | 1.88E-02 | WIF1 | 5.47 | 1.29E-02 | NUP210 | 4.09 | 1.26E-03 |
| ANK2 | 2.94 | 1.53E-02 | SOX8 | 5.47 | 2.72E-04 | PCSK5 | 4.09 | 1.00E-06 |
| CTSK | 2.92 | 8.50E-08 | ANKS1B | 5.45 | 1.00E-06 | KIAA1958 | 4.09 | 2.54E-06 |
| FABP3 | 2.92 | 1.47E-02 | TMEM178A | 5.44 | 8.62E-06 | EDA | 4.08 | 9.80E-03 |
| LAMA2 | 2.91 | 6.87E-04 | RSPO3 | 5.44 | 4.16E-03 | NR4A3 | 4.06 | 6.51E-08 |
| PCDHGA3 | 2.91 | 4.79E-02 | LANCL3 | 5.43 | 2.01E-02 | IGSF3 | 4.05 | 3.37E-03 |
| KYNU | 2.89 | 1.17E-05 | SUSD2 | 5.43 | 6.88E-04 | FAM84B | 4.02 | 1.08E-02 |
| KCND1 | 2.88 | 7.44E-03 | ANKK1 | 5.42 | 3.74E-05 | HAP1 | 4.02 | 2.20E-02 |
| NOVA1 | 2.88 | 1.67E-02 | TFAP2E | 5.42 | 1.24E-07 | CCDC81 | 3.99 | 8.23E-04 |
| SESN3 | 2.86 | 7.46E-03 | ASPN | 5.40 | 3.46E-05 | APCDD1 | 3.98 | 1.39E-05 |
| MAML3 | 2.84 | 7.87E-03 | RP11-474O21.5 | 5.36 | 1.37E-06 | HEY1 | 3.98 | 2.51E-03 |
| C1orf228 | 2.83 | 4.54E-02 | MERTK | 5.35 | 1.96E-06 | ZNF423 | 3.97 | 2.30E-04 |
| DDIT4 | 2.83 | 2.68E-02 | CA11 | 5.34 | 1.15E-03 | CD200 | 3.93 | 2.08E-02 |
| LTBP1 | 2.83 | 2.69E-03 | DHRS3 | 5.33 | 2.53E-09 | CAMK1G | 3.93 | 8.77E-04 |
| FMNL2 | 2.82 | 9.26E-03 | GBP1P1 | 5.32 | 1.40E-03 | NIPAL1 | 3.89 | 5.46E-04 |
| LTBP4 | 2.81 | 1.31E-02 | GRASP | 5.31 | 7.91E-05 | DTX1 | 3.88 | 8.94E-05 |
| OBSCN | 2.79 | 3.61E-02 | MAP1LC3C | 5.31 | 1.03E-03 | TENM2 | 3.85 | 1.57E-02 |
| RARRES3 | 2.79 | 1.33E-04 | GPRC5B | 5.31 | 1.40E-05 | GLDC | 3.85 | 2.98E-03 |
| PCDHGB2 | 2.79 | 8.03E-03 | CLCA2 | 5.31 | 2.12E-02 | WDR86 | 3.85 | 2.29E-02 |
| MAN1C1 | 2.75 | 1.53E-03 | ABCA8 | 5.30 | 3.49E-05 | ADAMTSL3 | 3.85 | 9.31E-05 |
| TMEM200A | 2.72 | 2.00E-02 | DPT | 5.28 | 1.47E-05 | NALCN | 3.85 | 6.05E-03 |
| ANKRD29 | 2.71 | 1.38E-02 | C1orf173 | 5.28 | 1.39E-02 | GNGT1 | 3.84 | 1.80E-02 |
| SERPINF1 | 2.71 | 7.60E-05 | HES1 | 5.28 | 1.54E-04 | CDH2 | 3.84 | 2.98E-03 |
| SRPX | 2.67 | 1.75E-03 | CRISPLD1 | 5.27 | 1.02E-06 | KCNK3 | 3.83 | 8.26E-03 |
| NCKAP5 | 2.66 | 1.27E-02 | PDK4 | 5.27 | 1.01E-03 | ABCA13 | 3.82 | 6.96E-05 |
| LPHN2 | 2.66 | 1.50E-02 | CH25H | 5.26 | 3.97E-04 | ENPP1 | 3.82 | 2.19E-05 |
| SOX9 | 2.64 | 1.09E-02 | RARRES3 | 5.25 | 5.76E-10 | DYNC1I1 | 3.81 | 2.71E-04 |
| BDKRB2 | 2.57 | 2.65E-04 | LRRC17 | 5.21 | 1.87E-07 | KLF15 | 3.80 | 6.59E-04 |
| TCF7L1 | 2.56 | 5.68E-03 | RSPO1 | 5.21 | 3.07E-03 | EGF | 3.79 | 8.66E-03 |
| TP53INP1 | 2.51 | 7.92E-03 | NALCN | 5.21 | 4.04E-04 | PDPN | 3.79 | 9.36E-05 |
| RGAG4 | 2.48 | 1.84E-02 | CHST2 | 5.20 | 2.74E-07 | UPB1 | 3.78 | 3.95E-02 |
| CD74 | 2.46 | 2.34E-03 | LINGO1 | 5.20 | 8.95E-06 | RP11-80H8.4 | 3.78 | 1.96E-03 |
| HSPA4L | 2.45 | 3.56E-02 | COL14A1 | 5.19 | 1.70E-02 | ITGA1 | 3.78 | 1.42E-06 |
| PLAG1 | 2.42 | 3.25E-02 | SLC7A10 | 5.17 | 2.28E-02 | FLRT1 | 3.75 | 4.01E-03 |
| AKAP12 | 2.42 | 1.93E-02 | AMIGO2 | 5.14 | 5.42E-09 | SGK1 | 3.75 | 4.28E-09 |
| PRICKLE2 | 2.39 | 3.78E-03 | PHACTR1 | 5.14 | 1.48E-05 | SLC38A4 | 3.74 | 4.91E-02 |
| LTBP3 | 2.33 | 3.23E-02 | THRB | 5.14 | 7.89E-07 | RRAGD | 3.73 | 7.61E-03 |
| MCC | 2.32 | 2.93E-02 | IGSF10 | 5.13 | 2.78E-03 | PLSCR1 | 3.71 | 2.23E-05 |
| EFNB1 | 2.29 | 4.64E-02 | FYB | 5.12 | 3.34E-04 | ACOT12 | 3.70 | 6.92E-03 |
| EDA2R | 2.24 | 3.13E-02 | ZNF467 | 5.12 | 1.09E-02 | MPV17L | 3.70 | 3.91E-04 |
| MCM7 | -2.28 | 3.45E-02 | TXLNB | 5.10 | 1.43E-03 | FIBCD1 | 3.68 | 4.26E-02 |
| CENPO | -2.32 | 2.44E-02 | LCNL1 | 5.08 | 1.27E-02 | CLCN5 | 3.67 | 1.06E-06 |
| TEAD4 | -2.35 | 4.66E-02 | ASPA | 5.08 | 5.06E-04 | BAMBI | 3.66 | 6.73E-03 |
| MCM8 | -2.36 | 4.32E-02 | MAP2 | 5.07 | 5.87E-06 | PACSIN1 | 3.66 | 3.51E-02 |
| RBL1 | -2.36 | 3.61E-02 | PLA2R1 | 5.07 | 3.68E-07 | FBLL1 | 3.66 | 2.16E-04 |
| EZR | -2.37 | 3.36E-02 | NGFR | 5.07 | 3.75E-03 | SORBS2 | 3.66 | 6.64E-04 |
| SLC9A3R1 | -2.37 | 3.81E-02 | LRRC16B | 5.07 | 1.10E-02 | KCNQ5 | 3.64 | 6.20E-03 |
| SMC4 | -2.38 | 4.06E-04 | CD36 | 5.04 | 2.73E-03 | TSPAN11 | 3.64 | 2.18E-03 |
| GMNN | -2.41 | 4.46E-02 | SALL4 | 5.04 | 9.55E-04 | IDO1 | 3.59 | 4.45E-02 |
| FANCI | -2.44 | 4.54E-02 | STOX1 | 5.04 | 7.82E-04 | PODXL | 3.58 | 8.81E-03 |
| CEP128 | -2.45 | 3.42E-02 | CYP26B1 | 5.03 | 2.59E-05 | LINC00472 | 3.58 | 1.40E-05 |
| H2AFZ | -2.45 | 3.78E-04 | GDF15 | 5.03 | 1.05E-06 | CNTN6 | 3.56 | 2.70E-02 |
| ATAD2 | -2.48 | 1.56E-04 | RP11-143K11.1 | 5.03 | 1.41E-04 | COL4A2 | 3.56 | 3.07E-03 |
| FBXO5 | -2.51 | 1.23E-02 | BMP3 | 5.01 | 4.27E-03 | SPAG1 | 3.56 | 1.87E-04 |
| CHAF1A | -2.53 | 5.62E-03 | CNTN1 | 5.01 | 3.79E-05 | FZD5 | 3.52 | 5.96E-04 |
| H2AFX | -2.54 | 2.98E-02 | RNF144A-AS1 | 5.01 | 1.24E-04 | LYPD6 | 3.52 | 8.39E-03 |
| MCM6 | -2.55 | 5.54E-04 | UNC79 | 4.99 | 2.20E-04 | FGFR3 | 3.51 | 2.05E-02 |
| VRK1 | -2.57 | 6.82E-03 | SPOCK2 | 4.98 | 4.91E-03 | SCNN1A | 3.51 | 1.30E-02 |
| HELLS | -2.58 | 7.64E-03 | AKR1B10 | 4.98 | 1.53E-03 | TRIM17 | 3.51 | 7.50E-03 |
| PSRC1 | -2.58 | 1.06E-03 | GCH1 | 4.97 | 2.91E-06 | ALPK3 | 3.50 | 3.11E-03 |
| MCM4 | -2.59 | 7.12E-04 | ABCA10 | 4.94 | 6.88E-03 | PTHLH | 3.49 | 4.23E-03 |
| CD274 | -2.62 | 1.48E-02 | ST8SIA6 | 4.90 | 8.70E-04 | DAB1 | 3.49 | 4.37E-04 |
| WWC1 | -2.63 | 3.20E-03 | SLC29A2 | 4.90 | 5.82E-05 | CHST2 | 3.47 | 7.18E-05 |
| GGH | -2.64 | 2.40E-03 | TXK | 4.89 | 5.86E-06 | DOK6 | 3.44 | 1.43E-02 |
| CHTF18 | -2.64 | 3.58E-03 | INA | 4.88 | 5.60E-05 | SCUBE3 | 3.44 | 6.57E-05 |
| TPM1 | -2.65 | 3.08E-05 | GPM6B | 4.88 | 5.55E-04 | TMEM132B | 3.43 | 2.43E-03 |
| PRIM1 | -2.66 | 2.02E-02 | GLDC | 4.88 | 1.39E-04 | GRASP | 3.42 | 4.60E-03 |
| C14orf80 | -2.69 | 6.88E-03 | RP1-45C12.1 | 4.88 | 9.55E-04 | MGAT3 | 3.41 | 1.77E-02 |
| SFXN2 | -2.71 | 1.51E-02 | SORL1 | 4.87 | 1.90E-03 | SULF1 | 3.41 | 3.43E-07 |
| UBE2S | -2.74 | 2.95E-03 | A2M | 4.86 | 1.34E-02 | FOXO1 | 3.40 | 2.74E-03 |
| LZTS1 | -2.77 | 1.10E-05 | HMCN1 | 4.82 | 6.44E-06 | RP11-567J20.1 | 3.39 | 3.28E-02 |
| DYSF | -2.77 | 3.87E-02 | DOK6 | 4.81 | 2.94E-04 | AC010649.1 | 3.38 | 1.86E-02 |
| DHFR | -2.78 | 5.68E-03 | ITIH3 | 4.81 | 9.12E-03 | GNGT2 | 3.35 | 2.22E-02 |
| DDX12P | -2.78 | 4.27E-02 | CDH23 | 4.80 | 4.60E-03 | SAMD5 | 3.34 | 2.55E-07 |
| DSCC1 | -2.79 | 1.16E-02 | LPHN1 | 4.79 | 6.67E-07 | LZTS1 | 3.33 | 9.80E-08 |
| KCTD14 | -2.81 | 8.20E-03 | OMD | 4.79 | 2.12E-04 | MYO16 | 3.29 | 2.13E-03 |
| KIF20B | -2.82 | 2.69E-03 | KBTBD11 | 4.78 | 5.46E-06 | OGN | 3.28 | 3.44E-03 |
| TRIM7 | -2.82 | 2.17E-03 | IGDCC4 | 4.77 | 2.52E-06 | RP11-125B21.2 | 3.27 | 3.31E-02 |
| ARHGAP11B | -2.82 | 1.86E-03 | GPNMB | 4.76 | 3.99E-07 | ASPN | 3.26 | 4.40E-03 |
| KNSTRN | -2.82 | 5.56E-05 | PIFO | 4.76 | 2.09E-03 | SOCS1 | 3.24 | 1.82E-04 |
| DKK1 | -2.83 | 9.22E-04 | PAK3 | 4.75 | 4.81E-04 | STK32B | 3.22 | 1.15E-06 |
| DLEU2 | -2.84 | 3.20E-02 | SESN3 | 4.74 | 4.43E-07 | CSRNP3 | 3.22 | 7.97E-04 |
| DEPTOR | -2.84 | 1.38E-02 | AKAP12 | 4.74 | 2.53E-09 | ANGPTL1 | 3.21 | 1.95E-03 |
| KRT19 | -2.84 | 7.49E-03 | GNGT1 | 4.72 | 1.29E-03 | CADM1 | 3.21 | 9.80E-08 |
| UBE2T | -2.85 | 3.34E-03 | GPX3 | 4.71 | 2.85E-04 | MEF2C | 3.18 | 4.09E-06 |
| FGF5 | -2.85 | 3.78E-02 | TMTC2 | 4.70 | 9.87E-06 | C5orf30 | 3.16 | 2.59E-02 |
| TUBA1B | -2.87 | 8.31E-03 | IFI44L | 4.70 | 6.01E-04 | PDGFA | 3.15 | 5.47E-04 |
| TAGLN | -2.87 | 9.26E-03 | RARB | 4.69 | 1.02E-06 | PLCH1 | 3.15 | 2.18E-03 |
| RP11-386G11.10 | -2.88 | 1.77E-02 | HAP1 | 4.69 | 2.93E-03 | MEST | 3.14 | 2.98E-03 |
| TRAIP | -2.89 | 1.32E-03 | EMID1 | 4.68 | 3.92E-03 | FGL2 | 3.14 | 8.38E-03 |
| GINS4 | -2.89 | 5.77E-03 | ELTD1 | 4.68 | 9.91E-03 | TSC22D3 | 3.12 | 2.94E-05 |
| CHAF1B | -2.89 | 8.33E-05 | LMO2 | 4.68 | 1.96E-03 | FAM134B | 3.12 | 8.15E-03 |
| RECQL4 | -2.90 | 4.15E-04 | ZSWIM5 | 4.67 | 3.80E-04 | DIRAS3 | 3.10 | 5.26E-04 |
| MXD3 | -2.91 | 7.92E-04 | TSPAN18 | 4.67 | 6.25E-06 | CORIN | 3.09 | 6.39E-03 |
| HAS3 | -2.91 | 1.35E-03 | TFPI2 | 4.66 | 2.13E-06 | HES1 | 3.08 | 3.22E-02 |
| BAIAP2L2 | -2.92 | 4.98E-02 | ACHE | 4.65 | 3.86E-03 | PTPLB | 3.07 | 1.98E-05 |
| CDCA7L | -2.92 | 1.83E-03 | ETV1 | 4.65 | 2.02E-06 | MAMLD1 | 3.07 | 3.37E-02 |
| DOCK2 | -2.94 | 4.73E-02 | ZNF423 | 4.65 | 2.59E-05 | OLFM2 | 3.05 | 3.40E-02 |
| MARCH4 | -2.94 | 3.12E-02 | ANGPTL1 | 4.64 | 1.57E-05 | PPM1L | 3.05 | 2.15E-04 |
| PSG4 | -2.94 | 1.07E-03 | MGAT3 | 4.62 | 5.10E-04 | NTRK3 | 2.99 | 3.33E-02 |
| GCNT4 | -2.95 | 2.40E-02 | TLR2 | 4.61 | 3.43E-02 | COL5A3 | 2.99 | 4.51E-02 |
| KIF22 | -2.96 | 1.53E-03 | SYNDIG1 | 4.60 | 2.64E-04 | KLF5 | 2.99 | 1.52E-02 |
| LBH | -2.96 | 1.15E-04 | CSRNP3 | 4.60 | 5.13E-06 | STARD8 | 2.97 | 1.77E-03 |
| LINC00968 | -2.96 | 1.19E-02 | MCOLN3 | 4.60 | 2.12E-02 | ABI3 | 2.96 | 1.48E-02 |
| KIAA1199 | -2.97 | 2.17E-03 | TMEM100 | 4.60 | 5.73E-08 | PBX3 | 2.96 | 1.42E-06 |
| HMGN2 | -2.97 | 1.58E-06 | PCSK9 | 4.59 | 1.78E-06 | PHACTR1 | 2.94 | 1.17E-02 |
| E2F7 | -2.99 | 4.15E-03 | SOBP | 4.58 | 3.93E-05 | KIT | 2.91 | 1.87E-04 |
| KPNA2 | -2.99 | 1.17E-03 | OAS1 | 4.57 | 1.22E-02 | VLDLR | 2.90 | 5.35E-04 |
| TMPO | -3.01 | 8.71E-05 | CNTNAP3B | 4.57 | 2.47E-04 | AKAP7 | 2.88 | 2.05E-02 |
| DGKI | -3.02 | 3.12E-02 | BEX2 | 4.54 | 8.93E-03 | TENM3 | 2.87 | 5.42E-04 |
| ODC1 | -3.04 | 1.50E-02 | DIRAS3 | 4.54 | 2.91E-06 | OTOGL | 2.87 | 1.68E-02 |
| KIAA1524 | -3.06 | 3.08E-05 | RP11-137H2.6 | 4.54 | 4.41E-04 | DCLK2 | 2.86 | 4.16E-03 |
| MB21D2 | -3.08 | 6.04E-03 | PLEKHS1 | 4.53 | 1.03E-02 | CAMK4 | 2.85 | 8.33E-03 |
| SGOL2 | -3.08 | 3.92E-05 | MEF2C | 4.52 | 2.79E-08 | FKBP5 | 2.84 | 1.37E-02 |
| CENPN | -3.10 | 6.88E-04 | ZNF608 | 4.52 | 1.57E-04 | NEDD9 | 2.83 | 4.19E-04 |
| ATAD5 | -3.10 | 1.44E-03 | HERC5 | 4.52 | 6.84E-03 | PTGFRN | 2.83 | 2.92E-02 |
| FANCB | -3.11 | 7.59E-03 | PLSCR1 | 4.50 | 6.94E-07 | PLA2R1 | 2.82 | 6.92E-03 |
| LMCD1 | -3.11 | 2.57E-03 | IBSP | 4.50 | 3.79E-05 | DKK1 | 2.80 | 1.53E-03 |
| XKR5 | -3.13 | 1.29E-02 | PCDHA4 | 4.49 | 3.91E-04 | MAP7 | 2.79 | 3.40E-04 |
| NCAPG2 | -3.13 | 7.23E-07 | HSD17B2 | 4.47 | 5.65E-03 | IRAK3 | 2.75 | 9.46E-04 |
| ZNF726 | -3.17 | 3.02E-02 | QPRT | 4.47 | 1.25E-08 | DLX1 | 2.66 | 2.23E-02 |
| PKN3 | -3.19 | 1.10E-05 | GAP43 | 4.46 | 8.82E-04 | SYNM | 2.65 | 1.35E-02 |
| CYTL1 | -3.22 | 4.22E-02 | SHISA3 | 4.44 | 3.87E-05 | ANKS1B | 2.63 | 1.90E-02 |
| TIMELESS | -3.24 | 8.03E-05 | ACKR4 | 4.44 | 5.84E-05 | ITPR1 | 2.63 | 1.18E-03 |
| EME1 | -3.25 | 5.46E-03 | TMEM132B | 4.43 | 3.46E-05 | PDE7B | 2.59 | 5.88E-05 |
| CDT1 | -3.25 | 5.67E-05 | PREX1 | 4.41 | 1.25E-04 | PCED1B | 2.59 | 2.43E-02 |
| CCNF | -3.25 | 8.33E-05 | PPM1L | 4.40 | 1.02E-06 | RGL1 | 2.57 | 1.95E-04 |
| RNASEH2A | -3.26 | 1.10E-05 | SH3BGRL2 | 4.38 | 7.34E-06 | SIMC1 | 2.56 | 3.21E-03 |
| GINS1 | -3.26 | 3.91E-03 | KCNT2 | 4.37 | 1.80E-02 | SLC46A3 | 2.53 | 4.54E-02 |
| KIF18A | -3.27 | 2.57E-05 | COL4A5 | 4.36 | 4.50E-04 | RARRES3 | 2.45 | 8.39E-04 |
| SLC8A1 | -3.28 | 9.90E-07 | LINC00472 | 4.36 | 8.71E-07 | AZIN1 | 2.43 | 1.70E-04 |
| RP11-424C20.2 | -3.28 | 3.81E-03 | TTBK1 | 4.33 | 8.10E-04 | MTMR10 | 2.36 | 3.44E-03 |
| MCAM | -3.29 | 1.32E-04 | CYP2J2 | 4.32 | 3.11E-04 | AIM1 | -2.45 | 1.13E-02 |
| UHRF1 | -3.30 | 5.44E-04 | DAPK1 | 4.30 | 7.87E-06 | PTX3 | -2.45 | 1.15E-02 |
| PRR11 | -3.30 | 1.30E-04 | OMG | 4.30 | 1.01E-02 | CD74 | -2.47 | 2.49E-03 |
| PTTG1 | -3.32 | 7.37E-04 | SOX5 | 4.29 | 1.15E-03 | S100A6 | -2.63 | 2.01E-03 |
| ATP8B1 | -3.32 | 3.93E-07 | CHST8 | 4.29 | 3.33E-05 | SIRPA | -2.63 | 6.68E-04 |
| XRCC2 | -3.32 | 8.37E-04 | RP11-203I2.1 | 4.29 | 2.74E-04 | SELPLG | -2.64 | 4.11E-02 |
| CENPI | -3.33 | 7.96E-04 | NR4A3 | 4.28 | 1.45E-08 | FSD1 | -2.69 | 1.57E-02 |
| BRCA1 | -3.33 | 3.83E-05 | RNF175 | 4.24 | 7.49E-04 | AKR1B1 | -2.69 | 1.34E-03 |
| CENPH | -3.34 | 6.02E-04 | CFH | 4.21 | 2.79E-08 | ATP8B1 | -2.70 | 7.37E-03 |
| CDKN2C | -3.35 | 3.93E-05 | SLC1A3 | 4.20 | 9.11E-07 | ITGB3 | -2.78 | 3.38E-02 |
| ZFPM2 | -3.35 | 3.62E-05 | JUP | 4.19 | 5.76E-10 | XYLT1 | -2.80 | 9.09E-03 |
| FANCD2 | -3.36 | 9.64E-05 | ALPK3 | 4.18 | 1.73E-04 | CD9 | -2.82 | 1.93E-03 |
| ESPL1 | -3.37 | 3.23E-03 | SCNN1A | 4.18 | 8.40E-04 | CIITA | -2.89 | 4.90E-03 |
| POC1A | -3.37 | 3.53E-07 | ESR1 | 4.17 | 5.25E-04 | SCN1B | -2.89 | 4.59E-04 |
| MELK | -3.37 | 1.19E-05 | GALNTL6 | 4.15 | 3.10E-04 | MRGPRF | -2.90 | 1.82E-03 |
| FEN1 | -3.38 | 4.27E-06 | SLC16A9 | 4.14 | 3.88E-04 | MSX2 | -2.90 | 8.86E-04 |
| CTNND2 | -3.38 | 3.32E-03 | C15orf48 | 4.14 | 5.91E-03 | LEF1 | -2.94 | 1.43E-02 |
| LIMS2 | -3.38 | 9.91E-04 | NTN1 | 4.12 | 1.04E-02 | NUAK2 | -2.95 | 1.80E-02 |
| ORC6 | -3.38 | 6.61E-04 | SSPO | 4.11 | 5.26E-03 | EBF3 | -2.95 | 1.20E-04 |
| RACGAP1 | -3.38 | 3.08E-05 | SFRP4 | 4.09 | 6.22E-04 | TMEM35 | -3.00 | 8.23E-04 |
| CDCA7 | -3.39 | 1.03E-04 | GPC4 | 4.08 | 7.46E-05 | ACE | -3.05 | 1.06E-03 |
| KIRREL3 | -3.40 | 2.13E-04 | SPRY4 | 4.08 | 7.94E-04 | SHROOM2 | -3.09 | 1.13E-02 |
| KIF24 | -3.41 | 2.11E-05 | CFI | 4.08 | 7.78E-06 | AKAP6 | -3.10 | 1.19E-03 |
| RP11-303E16.2 | -3.43 | 2.98E-03 | COL10A1 | 4.07 | 1.29E-06 | RRAD | -3.12 | 1.87E-02 |
| NDN | -3.43 | 5.53E-04 | ATP8A1 | 4.07 | 3.07E-03 | ABCC4 | -3.12 | 5.00E-03 |
| STIL | -3.43 | 1.93E-05 | FAM43A | 4.07 | 2.44E-06 | SUSD3 | -3.13 | 3.92E-03 |
| ASRGL1 | -3.44 | 2.60E-03 | CYGB | 4.06 | 4.67E-06 | GPER1 | -3.14 | 1.60E-03 |
| FST | -3.45 | 4.68E-02 | FRAS1 | 4.04 | 1.40E-02 | TRABD2A | -3.14 | 8.66E-03 |
| LYPD6 | -3.45 | 9.48E-03 | LAMA2 | 4.03 | 4.77E-07 | STEAP1 | -3.18 | 1.62E-03 |
| MOK | -3.47 | 1.02E-05 | DRD1 | 4.03 | 1.23E-03 | WISP1 | -3.21 | 1.70E-02 |
| GSG2 | -3.47 | 5.76E-04 | TRIM17 | 4.02 | 6.64E-04 | APCDD1L | -3.21 | 6.06E-05 |
| TMPO-AS1 | -3.49 | 3.81E-03 | CXCL10 | 4.02 | 2.82E-02 | SPOCD1 | -3.23 | 6.93E-06 |
| TCF19 | -3.49 | 4.56E-06 | C8orf34 | 4.00 | 1.17E-02 | AC006547.14 | -3.25 | 1.20E-03 |
| FAM132B | -3.50 | 4.20E-03 | RP11-292B8.1 | 3.98 | 2.05E-03 | CXCL6 | -3.33 | 3.33E-02 |
| ERBB3 | -3.51 | 1.47E-02 | ADAM22 | 3.98 | 7.09E-03 | S100A4 | -3.34 | 4.76E-03 |
| NEURL1B | -3.51 | 9.31E-04 | RP11-567J20.1 | 3.98 | 2.29E-03 | HTR7 | -3.38 | 1.04E-02 |
| GAS2L3 | -3.55 | 1.24E-05 | HIST1H3E | 3.96 | 3.07E-04 | PTPRU | -3.39 | 4.75E-05 |
| CDH2 | -3.55 | 9.21E-03 | ZBTB46 | 3.95 | 2.70E-05 | CLCA2 | -3.40 | 4.06E-03 |
| FGFR2 | -3.55 | 2.43E-04 | C3 | 3.95 | 3.07E-03 | REEP2 | -3.40 | 9.09E-07 |
| WDR76 | -3.55 | 3.20E-06 | C1orf145 | 3.95 | 3.79E-02 | FGF7 | -3.41 | 3.96E-03 |
| RP11-527H14.3 | -3.57 | 2.39E-03 | FAM213A | 3.92 | 5.43E-07 | BDKRB1 | -3.44 | 9.77E-03 |
| RFC3 | -3.59 | 6.32E-05 | ANK2 | 3.92 | 2.64E-05 | RNF157 | -3.47 | 3.00E-07 |
| BRIP1 | -3.60 | 1.09E-04 | MMP8 | 3.92 | 6.03E-03 | HMGA2 | -3.47 | 1.18E-06 |
| MCM2 | -3.60 | 4.79E-06 | DHRS2 | 3.90 | 4.39E-03 | C11orf45 | -3.48 | 1.15E-02 |
| HMGB2 | -3.60 | 2.98E-06 | LRMP | 3.89 | 6.17E-03 | TIMP1 | -3.48 | 4.41E-06 |
| METTL7B | -3.63 | 1.09E-03 | EGF | 3.88 | 2.76E-03 | RARRES2 | -3.52 | 1.65E-03 |
| ARHGAP11A | -3.63 | 7.71E-07 | FLRT2 | 3.88 | 3.45E-05 | PCDHGC5 | -3.55 | 1.80E-02 |
| POLE2 | -3.64 | 1.34E-04 | IGDCC3 | 3.87 | 7.72E-03 | GRIN3B | -3.57 | 1.37E-02 |
| CDC25A | -3.64 | 2.03E-04 | C19orf35 | 3.86 | 3.81E-02 | FILIP1L | -3.58 | 3.12E-04 |
| JPH2 | -3.66 | 1.30E-05 | COL4A4 | 3.86 | 7.28E-03 | HLA-DRA | -3.60 | 1.50E-03 |
| GINS3 | -3.69 | 6.67E-04 | SLC46A3 | 3.85 | 7.87E-06 | SLC1A2 | -3.60 | 2.54E-03 |
| SBSN | -3.69 | 1.47E-03 | HIST2H2BE | 3.85 | 1.61E-03 | PLEKHA6 | -3.61 | 4.97E-06 |
| NGF | -3.71 | 5.21E-04 | NFE2L3 | 3.84 | 1.35E-06 | APCDD1L-AS1 | -3.62 | 3.60E-02 |
| RIBC2 | -3.74 | 1.69E-02 | PPARGC1A | 3.84 | 8.14E-03 | GREM2 | -3.68 | 5.65E-07 |
| AC108142.1 | -3.76 | 3.34E-03 | GHR | 3.84 | 5.86E-06 | FAM180A | -3.68 | 4.60E-03 |
| FANCA | -3.76 | 2.03E-04 | PRCD | 3.84 | 3.81E-02 | RGS17 | -3.69 | 8.22E-04 |
| KCNK6 | -3.78 | 1.37E-04 | PTHLH | 3.84 | 5.46E-04 | PTGFR | -3.69 | 8.39E-04 |
| CENPE | -3.82 | 1.07E-05 | SRGAP3 | 3.83 | 2.64E-03 | INMT | -3.70 | 5.60E-06 |
| RAD54L | -3.85 | 3.45E-05 | RP11-125B21.2 | 3.83 | 2.01E-03 | RP13-463N16.6 | -3.74 | 2.73E-02 |
| SPC24 | -3.90 | 4.12E-05 | RP4-756G23.5 | 3.82 | 3.77E-03 | ARHGAP22 | -3.75 | 5.06E-04 |
| KIF4A | -3.91 | 3.70E-05 | MIR137HG | 3.82 | 3.84E-02 | STX1B | -3.75 | 1.70E-04 |
| CDCA3 | -3.92 | 1.93E-05 | SEMA6B | 3.82 | 3.15E-02 | ATP8B4 | -3.77 | 2.56E-02 |
| CENPW | -3.92 | 1.45E-05 | TSC22D3 | 3.82 | 4.69E-07 | SIGLEC15 | -3.78 | 6.50E-03 |
| MCM5 | -3.92 | 2.21E-06 | EDA | 3.81 | 7.15E-03 | CBLN2 | -3.78 | 7.16E-03 |
| FIBCD1 | -3.94 | 1.32E-02 | RPS6KA5 | 3.80 | 5.69E-03 | ITGA2 | -3.82 | 1.44E-04 |
| CENPF | -3.97 | 9.00E-05 | KCNMB4 | 3.80 | 4.51E-04 | TEK | -3.85 | 2.84E-02 |
| TRAC | -3.97 | 4.63E-03 | EML6 | 3.80 | 3.88E-02 | SERINC2 | -3.87 | 1.17E-04 |
| DMC1 | -3.98 | 1.32E-03 | ITPR1 | 3.79 | 3.44E-07 | RP11-47I22.3 | -3.87 | 8.66E-03 |
| MATN2 | -4.03 | 9.38E-06 | EFNB1 | 3.79 | 1.25E-08 | ST8SIA1 | -3.88 | 1.23E-02 |
| AURKA | -4.04 | 5.38E-06 | LPHN2 | 3.78 | 2.02E-06 | RP11-221N13.3 | -3.89 | 2.15E-04 |
| NEIL3 | -4.06 | 3.72E-04 | SOCS1 | 3.78 | 6.40E-06 | KCNJ15 | -3.89 | 9.34E-03 |
| KIF21B | -4.06 | 6.69E-03 | NRK | 3.77 | 1.22E-03 | APBB1IP | -3.90 | 1.85E-02 |
| MEST | -4.06 | 1.45E-05 | PYY2 | 3.77 | 3.70E-02 | ABCB1 | -3.97 | 2.27E-03 |
| CACNG4 | -4.07 | 2.06E-02 | SLC44A5 | 3.76 | 4.47E-02 | CASC10 | -3.99 | 2.80E-02 |
| LRRC2 | -4.08 | 2.38E-02 | DDIT4 | 3.75 | 4.78E-05 | HMGA1 | -4.05 | 1.34E-05 |
| FBXO43 | -4.08 | 4.21E-03 | CADM1 | 3.75 | 2.53E-09 | TMEM158 | -4.09 | 1.56E-05 |
| E2F1 | -4.09 | 1.78E-05 | FAM184A | 3.75 | 9.67E-03 | MOV10L1 | -4.09 | 1.59E-02 |
| CDCA2 | -4.09 | 1.56E-04 | PPP2R2B | 3.74 | 2.49E-03 | ENTPD1 | -4.10 | 3.16E-02 |
| TPX2 | -4.11 | 3.09E-05 | BCO2 | 3.73 | 7.52E-04 | LINC00519 | -4.15 | 8.26E-03 |
| MAMLD1 | -4.13 | 1.15E-04 | C4B | 3.72 | 1.60E-03 | FPR1 | -4.16 | 2.41E-02 |
| CENPK | -4.14 | 6.93E-05 | POSTN | 3.72 | 2.65E-06 | FENDRR | -4.17 | 5.60E-06 |
| NAP1L3 | -4.14 | 8.40E-04 | RYR2 | 3.72 | 4.39E-02 | AC114494.1 | -4.17 | 3.24E-02 |
| EZH2 | -4.15 | 6.87E-06 | ITGA1 | 3.71 | 1.00E-06 | WNT16 | -4.20 | 2.49E-03 |
| ZCCHC5 | -4.16 | 5.62E-03 | MMP16 | 3.70 | 6.16E-05 | C3 | -4.22 | 8.89E-06 |
| ATP10A | -4.16 | 9.22E-06 | TNFSF4 | 3.70 | 7.62E-04 | CEND1 | -4.32 | 7.97E-06 |
| LYPD6B | -4.17 | 5.56E-05 | FMNL2 | 3.69 | 1.53E-05 | TAC3 | -4.32 | 5.41E-03 |
| TOP2A | -4.19 | 9.51E-05 | FAM65C | 3.68 | 1.75E-07 | AHNAK2 | -4.36 | 4.17E-05 |
| CLEC3B | -4.21 | 7.12E-04 | SEMA5A | 3.68 | 3.71E-07 | INSC | -4.37 | 2.84E-02 |
| DLX3 | -4.21 | 7.13E-03 | CILP2 | 3.66 | 5.57E-04 | DGAT2 | -4.39 | 4.54E-02 |
| UCP2 | -4.22 | 4.58E-04 | MEIS2 | 3.66 | 1.69E-05 | CSDC2 | -4.44 | 8.23E-03 |
| ABI3BP | -4.22 | 4.31E-04 | FLRT1 | 3.65 | 1.97E-03 | SOX11 | -4.48 | 9.34E-03 |
| MAD2L1 | -4.22 | 7.59E-06 | HIST1H2BC | 3.65 | 7.88E-04 | TPPP3 | -4.53 | 4.38E-06 |
| SPAG5 | -4.24 | 3.65E-06 | SLC38A4 | 3.65 | 2.32E-02 | COX6B2 | -4.55 | 3.69E-02 |
| KIF11 | -4.25 | 1.62E-06 | ENPP1 | 3.64 | 1.65E-05 | DRD2 | -4.59 | 1.41E-04 |
| PPP1R14A | -4.27 | 3.52E-02 | RGL1 | 3.64 | 1.25E-08 | RGCC | -4.63 | 1.00E-06 |
| NUSAP1 | -4.27 | 1.36E-04 | RP11-161M6.2 | 3.64 | 4.91E-03 | RAB27B | -4.66 | 7.66E-05 |
| TICRR | -4.27 | 6.62E-05 | TTC18 | 3.64 | 4.16E-02 | DNAH5 | -4.69 | 6.50E-04 |
| KIF15 | -4.31 | 1.36E-04 | TEX15 | 3.63 | 6.95E-03 | KRT19 | -4.70 | 6.00E-03 |
| TACC3 | -4.32 | 6.29E-06 | AMY2B | 3.63 | 2.17E-02 | CPNE7 | -4.70 | 3.12E-06 |
| KIF2C | -4.32 | 9.90E-07 | VLDLR | 3.62 | 3.95E-06 | CLEC2B | -4.73 | 2.95E-04 |
| AUNIP | -4.33 | 5.02E-04 | REPS2 | 3.61 | 3.53E-02 | RP11-818F20.5 | -4.73 | 1.64E-03 |
| OLFM1 | -4.37 | 1.01E-04 | KCTD4 | 3.61 | 3.54E-02 | IL33 | -4.75 | 1.23E-04 |
| TRIP13 | -4.41 | 2.25E-05 | PTGIS | 3.60 | 2.14E-02 | OLFML2A | -4.78 | 1.46E-06 |
| KRT7 | -4.44 | 6.37E-05 | DUSP6 | 3.57 | 1.39E-04 | RP11-492E3.2 | -4.79 | 2.71E-04 |
| CDH4 | -4.45 | 2.77E-04 | ASTN2 | 3.56 | 1.81E-05 | HLA-DRB5 | -4.89 | 2.71E-03 |
| EXO1 | -4.46 | 1.10E-05 | GBP4 | 3.56 | 2.22E-06 | RP11-824M15.3 | -4.94 | 2.05E-02 |
| KIF23 | -4.47 | 7.21E-07 | RP11-712B9.2 | 3.56 | 4.85E-03 | LINC00856 | -4.95 | 7.61E-04 |
| KIF14 | -4.48 | 6.88E-05 | CACNB4 | 3.54 | 2.63E-02 | HLA-DRB1 | -4.96 | 2.14E-04 |
| CDC25C | -4.48 | 6.75E-05 | SVIL | 3.54 | 1.15E-06 | DLX5 | -5.00 | 7.52E-03 |
| CDC6 | -4.48 | 3.40E-05 | PRICKLE1 | 3.53 | 1.75E-07 | IGF2 | -5.03 | 4.46E-04 |
| CDCA5 | -4.49 | 3.20E-06 | FBLL1 | 3.52 | 1.18E-04 | FOXQ1 | -5.04 | 2.62E-03 |
| CDK1 | -4.50 | 1.68E-04 | PDPN | 3.52 | 9.28E-05 | GDF5OS | -5.09 | 8.27E-03 |
| PLK4 | -4.50 | 9.42E-06 | SEPT4 | 3.50 | 6.38E-03 | GREM1 | -5.23 | 1.28E-05 |
| ASPM | -4.50 | 3.13E-05 | ECM2 | 3.49 | 7.73E-05 | CCR1 | -5.25 | 1.15E-03 |
| MTFR2 | -4.50 | 1.16E-04 | KCNC3 | 3.48 | 2.38E-03 | PAQR5 | -5.30 | 9.61E-07 |
| CHAC2 | -4.50 | 1.10E-03 | TNFAIP8L3 | 3.48 | 7.24E-04 | WISP2 | -5.32 | 4.97E-03 |
| RAD51AP1 | -4.51 | 1.15E-04 | RNF144A | 3.48 | 2.48E-04 | GDF5 | -5.33 | 3.54E-04 |
| CATSPER1 | -4.51 | 7.49E-03 | SERPINI1 | 3.48 | 1.86E-03 | PPL | -5.42 | 5.40E-04 |
| CASZ1 | -4.52 | 6.27E-04 | LLGL2 | 3.47 | 2.50E-02 | KRT80 | -5.43 | 8.23E-04 |
| BLM | -4.53 | 1.70E-04 | MAN1C1 | 3.47 | 3.08E-06 | CDCP1 | -5.46 | 1.09E-06 |
| APOBEC3B | -4.58 | 2.08E-04 | OTOGL | 3.46 | 3.17E-04 | DIO3OS | -5.51 | 4.40E-03 |
| DMKN | -4.61 | 1.55E-03 | MAST4 | 3.44 | 1.34E-05 | TNC | -5.56 | 1.96E-07 |
| RAD51 | -4.61 | 2.34E-05 | FOXO1 | 3.43 | 8.74E-04 | SCG2 | -5.59 | 1.39E-05 |
| CDKN3 | -4.62 | 3.20E-06 | RAPGEF3 | 3.43 | 2.20E-03 | SEMA4D | -5.62 | 1.37E-02 |
| FOXM1 | -4.63 | 7.05E-06 | NR3C2 | 3.43 | 1.85E-03 | MMP1 | -5.63 | 1.52E-03 |
| CCNA2 | -4.63 | 1.37E-06 | TCN2 | 3.42 | 1.03E-03 | SBSN | -5.85 | 6.34E-03 |
| NRGN | -4.63 | 7.16E-03 | EPHA4 | 3.42 | 7.82E-04 | CACNG7 | -5.86 | 7.11E-03 |
| GINS2 | -4.65 | 8.45E-05 | SLC40A1 | 3.39 | 3.36E-03 | PCSK2 | -5.86 | 4.67E-02 |
| CCNB1 | -4.66 | 1.29E-06 | PIK3R3 | 3.39 | 8.17E-05 | MASP1 | -5.91 | 8.35E-04 |
| ACBD7 | -4.66 | 2.84E-03 | CTD-2215L10.1 | 3.39 | 6.76E-03 | DIO3 | -6.01 | 1.72E-03 |
| HJURP | -4.67 | 9.28E-05 | SHOX2 | 3.38 | 1.25E-05 | CCK | -6.08 | 5.94E-06 |
| TM4SF20 | -4.67 | 3.46E-03 | KIAA1958 | 3.37 | 7.84E-06 | KRTAP1-5 | -6.13 | 3.52E-02 |
| NDC80 | -4.67 | 2.60E-06 | SFRP1 | 3.36 | 8.45E-09 | DLX6 | -6.23 | 1.34E-02 |
| ORC1 | -4.68 | 8.36E-06 | B3GALT2 | 3.35 | 1.57E-02 | CTC-436P18.3 | -6.26 | 4.71E-05 |
| FAM83D | -4.68 | 3.03E-05 | GPR37 | 3.35 | 4.63E-02 | SLC14A1 | -6.28 | 1.91E-09 |
| PBK | -4.68 | 2.26E-05 | PDE3A | 3.35 | 1.52E-04 | SP6 | -6.54 | 2.48E-04 |
| ANLN | -4.69 | 9.90E-06 | IRAK3 | 3.35 | 5.87E-06 | TRPA1 | -6.89 | 1.91E-04 |
| POLQ | -4.69 | 1.19E-05 | TP63 | 3.33 | 2.31E-02 | SCN4B | -6.97 | 4.12E-04 |
| PLEK2 | -4.71 | 2.56E-03 | CCDC170 | 3.32 | 4.30E-03 | SCN2B | -7.00 | 8.72E-04 |
| DIAPH3 | -4.72 | 1.77E-06 | HIST1H2AC | 3.32 | 6.48E-04 | KYNU | -7.32 | 1.87E-08 |
| MND1 | -4.73 | 3.73E-04 | CAMK1G | 3.32 | 2.68E-03 | LPAR3 | -7.70 | 8.67E-05 |
| GMFG | -4.75 | 7.89E-04 | BTG2 | 3.30 | 1.24E-04 | CALB2 | -8.25 | 4.97E-06 |
| NCAPG | -4.75 | 4.12E-05 | TRIM9 | 3.30 | 2.33E-02 | PTPRN | -10.28 | 8.82E-08 |
| OIP5 | -4.76 | 1.93E-04 | CES4A | 3.29 | 3.24E-04 |  |  |  |
| CST6 | -4.78 | 2.28E-04 | CORIN | 3.29 | 9.08E-04 |  |  |  |
| SGOL1 | -4.79 | 1.13E-04 | SLIT3 | 3.27 | 2.78E-04 |  |  |  |
| DEPDC1B | -4.79 | 1.77E-05 | CLCN5 | 3.27 | 1.96E-06 |  |  |  |
| CASC5 | -4.80 | 2.13E-04 | MAP7 | 3.27 | 5.17E-06 |  |  |  |
| COL4A1 | -4.80 | 1.53E-03 | KIAA1377 | 3.23 | 3.36E-06 |  |  |  |
| PRC1 | -4.80 | 5.38E-06 | IFIT2 | 3.23 | 5.92E-05 |  |  |  |
| IQGAP3 | -4.81 | 1.63E-05 | FAM189A1 | 3.21 | 4.21E-02 |  |  |  |
| NEK2 | -4.82 | 5.88E-05 | LIFR | 3.20 | 1.26E-05 |  |  |  |
| KIF20A | -4.82 | 1.77E-05 | RP11-541N10.3 | 3.20 | 4.23E-03 |  |  |  |
| TENM2 | -4.84 | 4.50E-04 | ATF3 | 3.18 | 2.02E-02 |  |  |  |
| INHBB | -4.84 | 3.46E-03 | CLDN11 | 3.16 | 1.25E-06 |  |  |  |
| GIPR | -4.87 | 1.08E-03 | SEMA3E | 3.15 | 3.89E-02 |  |  |  |
| CENPU | -4.87 | 1.79E-03 | NAALAD2 | 3.15 | 4.52E-02 |  |  |  |
| LMNB1 | -4.88 | 3.08E-05 | TGFB3 | 3.13 | 2.17E-02 |  |  |  |
| HMMR | -4.89 | 3.03E-05 | DAB1 | 3.13 | 6.75E-04 |  |  |  |
| ZNF367 | -4.89 | 3.34E-05 | FGF14 | 3.13 | 4.37E-03 |  |  |  |
| CCNB2 | -4.89 | 3.20E-06 | MYO16 | 3.12 | 1.81E-03 |  |  |  |
| MKI67 | -4.89 | 2.42E-04 | FAM134B | 3.12 | 3.03E-03 |  |  |  |
| COL4A2 | -4.89 | 1.84E-05 | ITGA10 | 3.12 | 5.31E-03 |  |  |  |
| AC087645.1 | -4.90 | 1.17E-03 | PPP4R4 | 3.11 | 8.14E-03 |  |  |  |
| MYPN | -4.90 | 2.63E-07 | TP53INP1 | 3.11 | 6.44E-06 |  |  |  |
| ASF1B | -4.92 | 5.56E-05 | APCDD1 | 3.11 | 1.16E-03 |  |  |  |
| PENK | -4.94 | 9.13E-06 | ISYNA1 | 3.10 | 6.84E-04 |  |  |  |
| CCNE2 | -4.94 | 7.77E-05 | SLC2A9 | 3.10 | 1.03E-03 |  |  |  |
| BUB1B | -4.98 | 3.08E-05 | TXNIP | 3.09 | 2.13E-04 |  |  |  |
| C11orf82 | -5.01 | 3.02E-05 | MFAP4 | 3.08 | 4.52E-03 |  |  |  |
| NUF2 | -5.01 | 1.20E-04 | METTL7A | 3.08 | 1.49E-02 |  |  |  |
| WDR62 | -5.01 | 1.63E-06 | TPD52L1 | 3.06 | 8.75E-06 |  |  |  |
| CENPA | -5.02 | 2.10E-06 | FZD5 | 3.05 | 1.60E-03 |  |  |  |
| PLK1 | -5.02 | 1.17E-06 | SAMHD1 | 3.05 | 2.25E-04 |  |  |  |
| DTL | -5.04 | 1.80E-04 | PCDHB14 | 3.05 | 5.84E-03 |  |  |  |
| NCAPH | -5.06 | 1.94E-06 | MAPK10 | 3.05 | 1.80E-03 |  |  |  |
| GTSE1 | -5.07 | 7.85E-06 | OLFML2B | 3.05 | 3.95E-05 |  |  |  |
| PSG1 | -5.08 | 6.12E-04 | NCKAP5 | 3.04 | 1.59E-04 |  |  |  |
| DEPDC1 | -5.11 | 3.40E-05 | FAM107A | 3.04 | 3.76E-02 |  |  |  |
| BUB1 | -5.12 | 7.59E-06 | PIK3R1 | 3.04 | 2.85E-06 |  |  |  |
| CDCA8 | -5.12 | 8.92E-06 | PLXDC2 | 3.04 | 5.57E-05 |  |  |  |
| SHCBP1 | -5.12 | 1.64E-05 | AC068499.10 | 3.03 | 2.77E-02 |  |  |  |
| ERCC6L | -5.13 | 1.06E-04 | B3GALNT1 | 3.03 | 1.23E-05 |  |  |  |
| KIFC1 | -5.14 | 4.89E-06 | DACT3 | 3.03 | 4.73E-04 |  |  |  |
| TSPAN2 | -5.16 | 1.83E-03 | NEDD9 | 3.02 | 3.20E-05 |  |  |  |
| KIAA0101 | -5.19 | 4.79E-06 | GNGT2 | 3.01 | 3.84E-02 |  |  |  |
| CEP55 | -5.20 | 6.48E-06 | SEPP1 | 3.01 | 3.57E-02 |  |  |  |
| CKAP2L | -5.22 | 4.78E-05 | COLQ | 2.98 | 4.82E-02 |  |  |  |
| TTK | -5.22 | 3.20E-06 | PBX1 | 2.98 | 3.38E-03 |  |  |  |
| TROAP | -5.23 | 1.63E-06 | AF127936.7 | 2.96 | 2.07E-03 |  |  |  |
| PKP1 | -5.26 | 1.27E-04 | ATP9A | 2.96 | 1.59E-04 |  |  |  |
| ZWINT | -5.27 | 3.18E-06 | LGMN | 2.95 | 6.60E-04 |  |  |  |
| CLSPN | -5.31 | 2.35E-05 | KCND1 | 2.94 | 2.54E-03 |  |  |  |
| FAM111B | -5.32 | 4.02E-04 | ITGBL1 | 2.93 | 4.50E-05 |  |  |  |
| DLGAP5 | -5.32 | 1.66E-05 | PLAG1 | 2.93 | 2.77E-05 |  |  |  |
| UBE2C | -5.33 | 1.86E-05 | CSPG5 | 2.93 | 3.16E-02 |  |  |  |
| SKA3 | -5.34 | 1.74E-05 | C5AR1 | 2.93 | 5.07E-03 |  |  |  |
| GRPR | -5.36 | 1.02E-05 | KCTD12 | 2.92 | 7.75E-04 |  |  |  |
| ESCO2 | -5.36 | 7.46E-04 | PCDHGA3 | 2.92 | 2.52E-02 |  |  |  |
| CCDC85A | -5.37 | 3.27E-04 | PCDH18 | 2.91 | 1.03E-03 |  |  |  |
| HAPLN1 | -5.37 | 3.70E-07 | ARHGEF3 | 2.91 | 6.43E-04 |  |  |  |
| NPTX2 | -5.39 | 1.36E-04 | MED12L | 2.90 | 3.85E-02 |  |  |  |
| IL7R | -5.39 | 1.43E-09 | TTYH2 | 2.90 | 1.27E-03 |  |  |  |
| BIRC5 | -5.42 | 2.50E-06 | SSPN | 2.89 | 7.32E-05 |  |  |  |
| OXTR | -5.44 | 7.68E-08 | RASGRP3 | 2.88 | 3.83E-03 |  |  |  |
| FAM19A5 | -5.47 | 1.05E-03 | HCN2 | 2.88 | 2.46E-03 |  |  |  |
| PSG5 | -5.48 | 8.70E-09 | DISC1 | 2.85 | 9.94E-05 |  |  |  |
| CENPM | -5.62 | 1.93E-05 | CDKN2B | 2.83 | 2.67E-02 |  |  |  |
| LAMP5 | -5.62 | 1.31E-02 | PDCD4-AS1 | 2.83 | 1.62E-02 |  |  |  |
| FAM64A | -5.64 | 2.74E-05 | AC009404.2 | 2.83 | 4.00E-03 |  |  |  |
| SPC25 | -5.64 | 3.08E-05 | KLF15 | 2.81 | 1.78E-02 |  |  |  |
| LRRC15 | -5.67 | 6.98E-07 | TCF7L1 | 2.81 | 1.57E-04 |  |  |  |
| CPA4 | -5.68 | 3.34E-02 | RNF207 | 2.81 | 1.05E-03 |  |  |  |
| MYOCD | -5.68 | 2.51E-03 | NCAM1 | 2.81 | 2.26E-02 |  |  |  |
| AURKB | -5.70 | 5.38E-06 | CCPG1 | 2.79 | 2.52E-02 |  |  |  |
| E2F2 | -5.71 | 1.43E-04 | GPC6 | 2.79 | 1.03E-02 |  |  |  |
| KRT14 | -5.74 | 2.68E-03 | APP | 2.79 | 9.35E-05 |  |  |  |
| TK1 | -5.74 | 1.34E-07 | TSPAN14 | 2.79 | 4.52E-03 |  |  |  |
| SKA1 | -5.75 | 2.14E-05 | WDR63 | 2.78 | 1.34E-02 |  |  |  |
| KISS1 | -5.77 | 2.90E-06 | KCNJ2 | 2.78 | 2.59E-02 |  |  |  |
| KIF18B | -5.77 | 5.56E-05 | TSPAN9 | 2.77 | 5.70E-05 |  |  |  |
| HR | -5.78 | 1.08E-03 | PID1 | 2.77 | 1.41E-02 |  |  |  |
| MMP3 | -5.80 | 1.17E-04 | CYP27A1 | 2.76 | 1.90E-03 |  |  |  |
| CDC20 | -5.82 | 8.49E-06 | KLHL24 | 2.76 | 4.61E-03 |  |  |  |
| CARD11 | -5.82 | 4.51E-04 | TRNP1 | 2.74 | 4.79E-03 |  |  |  |
| RRM2 | -5.82 | 1.02E-05 | PTGES | 2.73 | 1.40E-02 |  |  |  |
| KRTAP2-3 | -5.88 | 2.92E-05 | FZD4 | 2.73 | 2.90E-03 |  |  |  |
| KRT33B | -5.91 | 1.37E-06 | AKAP7 | 2.71 | 2.50E-02 |  |  |  |
| E2F8 | -5.93 | 2.88E-04 | FNBP1L | 2.71 | 1.22E-02 |  |  |  |
| CDC45 | -6.03 | 2.92E-05 | TIMP3 | 2.71 | 1.94E-02 |  |  |  |
| MCM10 | -6.11 | 5.61E-05 | LSAMP | 2.69 | 2.18E-02 |  |  |  |
| ELN | -6.15 | 6.48E-06 | SORBS2 | 2.69 | 3.16E-02 |  |  |  |
| PKMYT1 | -6.19 | 5.39E-06 | MAML3 | 2.69 | 1.70E-02 |  |  |  |
| SAPCD2 | -6.31 | 2.92E-05 | MPV17L | 2.69 | 2.16E-02 |  |  |  |
| KRT34 | -6.35 | 2.60E-06 | PGAP1 | 2.68 | 7.68E-03 |  |  |  |
| GFRA2 | -6.37 | 2.90E-06 | HHAT | 2.68 | 5.52E-03 |  |  |  |
| BRINP1 | -6.41 | 1.74E-05 | MDK | 2.68 | 1.22E-03 |  |  |  |
| PALD1 | -6.54 | 6.60E-06 | FAP | 2.68 | 1.12E-03 |  |  |  |
| MYBL2 | -6.86 | 5.38E-06 | SPOCK1 | 2.68 | 6.62E-03 |  |  |  |
| NUP210 | -6.99 | 6.04E-07 | HIST1H2BK | 2.67 | 4.38E-04 |  |  |  |
| SYNPO2L | -7.15 | 1.60E-06 | HIC1 | 2.67 | 7.32E-03 |  |  |  |
| LINC00707 | -7.55 | 1.27E-06 | AFF3 | 2.66 | 4.84E-03 |  |  |  |
| ACAN | -7.59 | 7.11E-04 | FAT4 | 2.65 | 3.29E-02 |  |  |  |
|  |  |  | TNIK | 2.65 | 7.23E-03 |  |  |  |
|  |  |  | ARHGAP24 | 2.65 | 1.37E-04 |  |  |  |
|  |  |  | PLXNA2 | 2.64 | 2.29E-02 |  |  |  |
|  |  |  | SEMA4G | 2.64 | 4.50E-02 |  |  |  |
|  |  |  | CUBN | 2.63 | 1.85E-04 |  |  |  |
|  |  |  | ACVR2A | 2.62 | 2.83E-02 |  |  |  |
|  |  |  | STARD8 | 2.62 | 1.20E-02 |  |  |  |
|  |  |  | PELI1 | 2.62 | 4.91E-03 |  |  |  |
|  |  |  | C5orf56 | 2.61 | 2.81E-03 |  |  |  |
|  |  |  | PDGFA | 2.61 | 1.03E-02 |  |  |  |
|  |  |  | FZD1 | 2.61 | 3.62E-02 |  |  |  |
|  |  |  | RP4-758J24.5 | 2.61 | 2.61E-02 |  |  |  |
|  |  |  | DLX1 | 2.60 | 1.83E-02 |  |  |  |
|  |  |  | CLSTN2 | 2.60 | 8.70E-03 |  |  |  |
|  |  |  | RORA | 2.60 | 2.29E-03 |  |  |  |
|  |  |  | APLP2 | 2.60 | 1.53E-05 |  |  |  |
|  |  |  | PLSCR4 | 2.59 | 2.29E-03 |  |  |  |
|  |  |  | SESN1 | 2.56 | 7.34E-03 |  |  |  |
|  |  |  | RHOBTB3 | 2.55 | 3.14E-03 |  |  |  |
|  |  |  | CA5B | 2.55 | 4.11E-03 |  |  |  |
|  |  |  | RP11-400K9.4 | 2.55 | 3.84E-02 |  |  |  |
|  |  |  | PBX3 | 2.54 | 5.68E-05 |  |  |  |
|  |  |  | LAMA3 | 2.52 | 3.99E-02 |  |  |  |
|  |  |  | ABHD2 | 2.48 | 1.70E-02 |  |  |  |
|  |  |  | ZDHHC14 | 2.48 | 9.54E-05 |  |  |  |
|  |  |  | ETV5 | 2.46 | 2.41E-02 |  |  |  |
|  |  |  | SLC44A1 | 2.45 | 6.51E-03 |  |  |  |
|  |  |  | BVES | 2.44 | 2.42E-02 |  |  |  |
|  |  |  | PCED1B | 2.44 | 4.63E-02 |  |  |  |
|  |  |  | SNED1 | 2.43 | 3.52E-02 |  |  |  |
|  |  |  | LXN | 2.43 | 1.65E-02 |  |  |  |
|  |  |  | SERPINF1 | 2.42 | 3.07E-03 |  |  |  |
|  |  |  | SIMC1 | 2.42 | 8.89E-03 |  |  |  |
|  |  |  | FBXW7 | 2.41 | 4.54E-02 |  |  |  |
|  |  |  | TSPYL2 | 2.40 | 8.19E-03 |  |  |  |
|  |  |  | TRIB2 | 2.37 | 1.30E-03 |  |  |  |
|  |  |  | CTSK | 2.36 | 8.29E-05 |  |  |  |
|  |  |  | HAS3 | 2.35 | 3.61E-03 |  |  |  |
|  |  |  | SULF1 | 2.32 | 3.84E-02 |  |  |  |
|  |  |  | GPR153 | 2.31 | 4.90E-02 |  |  |  |
|  |  |  | RABGAP1L | 2.28 | 1.81E-02 |  |  |  |
|  |  |  | ZMAT3 | 2.27 | 6.41E-03 |  |  |  |
|  |  |  | SAMD5 | 2.25 | 1.81E-02 |  |  |  |
|  |  |  | OSBPL3 | 2.21 | 2.62E-02 |  |  |  |
|  |  |  | H2AFZ | -2.24 | 1.95E-02 |  |  |  |
|  |  |  | S100A10 | -2.24 | 2.32E-02 |  |  |  |
|  |  |  | TONSL | -2.28 | 4.05E-02 |  |  |  |
|  |  |  | RBL1 | -2.30 | 4.96E-02 |  |  |  |
|  |  |  | RRM1 | -2.31 | 1.60E-02 |  |  |  |
|  |  |  | KIAA1524 | -2.31 | 4.42E-02 |  |  |  |
|  |  |  | ATAD2 | -2.33 | 1.55E-03 |  |  |  |
|  |  |  | PPIF | -2.34 | 4.56E-02 |  |  |  |
|  |  |  | HERC4 | -2.35 | 1.72E-03 |  |  |  |
|  |  |  | CHEK1 | -2.40 | 5.95E-03 |  |  |  |
|  |  |  | TMPO | -2.41 | 2.92E-02 |  |  |  |
|  |  |  | HELLS | -2.42 | 2.29E-02 |  |  |  |
|  |  |  | UAP1 | -2.43 | 7.40E-03 |  |  |  |
|  |  |  | C14orf80 | -2.43 | 4.23E-02 |  |  |  |
|  |  |  | EZH2 | -2.45 | 2.50E-02 |  |  |  |
|  |  |  | TTC7A | -2.47 | 2.96E-03 |  |  |  |
|  |  |  | KIF24 | -2.48 | 9.15E-03 |  |  |  |
|  |  |  | RFC4 | -2.49 | 4.09E-02 |  |  |  |
|  |  |  | TEAD4 | -2.50 | 2.06E-03 |  |  |  |
|  |  |  | CHAF1A | -2.51 | 3.11E-03 |  |  |  |
|  |  |  | MXD3 | -2.53 | 1.12E-02 |  |  |  |
|  |  |  | CD9 | -2.53 | 2.03E-02 |  |  |  |
|  |  |  | MCM2 | -2.54 | 3.61E-03 |  |  |  |
|  |  |  | TYMS | -2.55 | 1.44E-02 |  |  |  |
|  |  |  | RECQL4 | -2.55 | 4.64E-03 |  |  |  |
|  |  |  | PLP2 | -2.56 | 7.03E-03 |  |  |  |
|  |  |  | ATAD5 | -2.56 | 3.18E-02 |  |  |  |
|  |  |  | ARNT2 | -2.57 | 9.65E-03 |  |  |  |
|  |  |  | UBE2T | -2.57 | 1.56E-02 |  |  |  |
|  |  |  | GGH | -2.57 | 2.18E-03 |  |  |  |
|  |  |  | SLC9A3R1 | -2.58 | 9.88E-04 |  |  |  |
|  |  |  | FSD1 | -2.58 | 2.37E-02 |  |  |  |
|  |  |  | SH3BGRL3 | -2.59 | 7.85E-03 |  |  |  |
|  |  |  | BRCA1 | -2.61 | 2.80E-03 |  |  |  |
|  |  |  | PIK3CD | -2.62 | 8.78E-04 |  |  |  |
|  |  |  | FEN1 | -2.62 | 3.34E-04 |  |  |  |
|  |  |  | HSD3B7 | -2.63 | 4.27E-05 |  |  |  |
|  |  |  | PRIM1 | -2.63 | 1.14E-02 |  |  |  |
|  |  |  | HMGA1 | -2.64 | 3.81E-02 |  |  |  |
|  |  |  | KCNK6 | -2.65 | 2.04E-02 |  |  |  |
|  |  |  | STIL | -2.65 | 1.22E-03 |  |  |  |
|  |  |  | S100A6 | -2.66 | 7.27E-04 |  |  |  |
|  |  |  | NCAPG2 | -2.66 | 8.83E-06 |  |  |  |
|  |  |  | FANCI | -2.67 | 1.48E-03 |  |  |  |
|  |  |  | FXYD5 | -2.67 | 5.71E-04 |  |  |  |
|  |  |  | GAS2L3 | -2.67 | 9.13E-04 |  |  |  |
|  |  |  | AIM1 | -2.68 | 1.60E-04 |  |  |  |
|  |  |  | CENPH | -2.69 | 1.19E-02 |  |  |  |
|  |  |  | SELPLG | -2.69 | 1.13E-02 |  |  |  |
|  |  |  | NDC80 | -2.70 | 1.11E-03 |  |  |  |
|  |  |  | ANKRD13A | -2.71 | 1.21E-04 |  |  |  |
|  |  |  | PRR16 | -2.71 | 3.25E-02 |  |  |  |
|  |  |  | GINS1 | -2.72 | 3.24E-02 |  |  |  |
|  |  |  | CASS4 | -2.73 | 4.51E-02 |  |  |  |
|  |  |  | E2F1 | -2.73 | 2.92E-03 |  |  |  |
|  |  |  | RNASEH2A | -2.74 | 1.59E-04 |  |  |  |
|  |  |  | ECT2 | -2.74 | 3.24E-04 |  |  |  |
|  |  |  | WHSC1 | -2.74 | 2.31E-04 |  |  |  |
|  |  |  | CAV1 | -2.74 | 1.52E-03 |  |  |  |
|  |  |  | CBR3 | -2.74 | 4.26E-05 |  |  |  |
|  |  |  | C17orf53 | -2.74 | 6.76E-03 |  |  |  |
|  |  |  | BRIP1 | -2.75 | 3.90E-03 |  |  |  |
|  |  |  | EZR | -2.75 | 8.94E-05 |  |  |  |
|  |  |  | ACOT11 | -2.76 | 2.46E-02 |  |  |  |
|  |  |  | TUBA4A | -2.76 | 1.51E-02 |  |  |  |
|  |  |  | CENPF | -2.76 | 1.62E-02 |  |  |  |
|  |  |  | KIF18A | -2.78 | 2.43E-04 |  |  |  |
|  |  |  | FANCA | -2.78 | 8.21E-03 |  |  |  |
|  |  |  | JPH2 | -2.79 | 4.63E-04 |  |  |  |
|  |  |  | TMPO-AS1 | -2.80 | 4.15E-02 |  |  |  |
|  |  |  | CIT | -2.81 | 2.40E-02 |  |  |  |
|  |  |  | BRCA2 | -2.82 | 1.24E-02 |  |  |  |
|  |  |  | HMGB2 | -2.83 | 1.24E-04 |  |  |  |
|  |  |  | SH2D4A | -2.83 | 6.79E-05 |  |  |  |
|  |  |  | SIRPA | -2.84 | 2.49E-05 |  |  |  |
|  |  |  | MTSS1L | -2.84 | 4.03E-06 |  |  |  |
|  |  |  | E2F7 | -2.85 | 4.28E-03 |  |  |  |
|  |  |  | AC006547.14 | -2.86 | 1.11E-02 |  |  |  |
|  |  |  | FANCD2 | -2.86 | 6.87E-04 |  |  |  |
|  |  |  | EDARADD | -2.86 | 1.35E-02 |  |  |  |
|  |  |  | MGARP | -2.86 | 3.16E-02 |  |  |  |
|  |  |  | ARHGAP22 | -2.87 | 3.62E-02 |  |  |  |
|  |  |  | CLCF1 | -2.89 | 3.20E-04 |  |  |  |
|  |  |  | KCTD14 | -2.89 | 2.11E-03 |  |  |  |
|  |  |  | RGMB | -2.89 | 1.69E-02 |  |  |  |
|  |  |  | CLSPN | -2.90 | 3.26E-03 |  |  |  |
|  |  |  | FADS3 | -2.90 | 4.37E-03 |  |  |  |
|  |  |  | NUP210 | -2.90 | 2.69E-04 |  |  |  |
|  |  |  | PTGFR | -2.90 | 3.81E-02 |  |  |  |
|  |  |  | TCF19 | -2.91 | 4.29E-05 |  |  |  |
|  |  |  | POLR3G | -2.92 | 1.12E-02 |  |  |  |
|  |  |  | KNSTRN | -2.93 | 9.40E-06 |  |  |  |
|  |  |  | PM20D2 | -2.93 | 1.88E-03 |  |  |  |
|  |  |  | ANGPTL2 | -2.94 | 1.66E-02 |  |  |  |
|  |  |  | GALNT14 | -2.95 | 1.78E-04 |  |  |  |
|  |  |  | FANCB | -2.95 | 8.24E-03 |  |  |  |
|  |  |  | ADCY7 | -2.96 | 2.40E-03 |  |  |  |
|  |  |  | WWC1 | -2.97 | 6.25E-05 |  |  |  |
|  |  |  | CHAF1B | -2.97 | 1.76E-05 |  |  |  |
|  |  |  | ASPM | -2.97 | 2.56E-03 |  |  |  |
|  |  |  | PLEKHG4B | -2.98 | 4.60E-03 |  |  |  |
|  |  |  | MIR503HG | -2.98 | 2.57E-03 |  |  |  |
|  |  |  | LMNB1 | -2.98 | 3.77E-03 |  |  |  |
|  |  |  | TAGLN2 | -2.99 | 1.00E-04 |  |  |  |
|  |  |  | RP5-1198O20.4 | -2.99 | 1.65E-02 |  |  |  |
|  |  |  | RGCC | -2.99 | 2.85E-03 |  |  |  |
|  |  |  | MSX2 | -2.99 | 1.69E-04 |  |  |  |
|  |  |  | CDKL1 | -3.00 | 1.28E-02 |  |  |  |
|  |  |  | SOCS2 | -3.00 | 4.45E-02 |  |  |  |
|  |  |  | FENDRR | -3.02 | 1.90E-03 |  |  |  |
|  |  |  | PRR11 | -3.02 | 2.76E-04 |  |  |  |
|  |  |  | SHMT1 | -3.03 | 1.73E-04 |  |  |  |
|  |  |  | MTFR2 | -3.03 | 5.92E-03 |  |  |  |
|  |  |  | NCEH1 | -3.04 | 2.94E-04 |  |  |  |
|  |  |  | DIAPH3 | -3.04 | 7.09E-05 |  |  |  |
|  |  |  | C12orf75 | -3.05 | 1.67E-05 |  |  |  |
|  |  |  | SLC17A9 | -3.05 | 3.11E-04 |  |  |  |
|  |  |  | MBOAT1 | -3.06 | 5.55E-04 |  |  |  |
|  |  |  | CRISPLD2 | -3.06 | 4.57E-02 |  |  |  |
|  |  |  | MCM5 | -3.07 | 4.18E-05 |  |  |  |
|  |  |  | POLQ | -3.08 | 4.41E-04 |  |  |  |
|  |  |  | WDR76 | -3.08 | 8.62E-06 |  |  |  |
|  |  |  | LY6K | -3.08 | 2.81E-03 |  |  |  |
|  |  |  | RP11-572C15.6 | -3.09 | 1.63E-03 |  |  |  |
|  |  |  | BEX1 | -3.10 | 3.86E-03 |  |  |  |
|  |  |  | MAD2L1 | -3.10 | 1.05E-04 |  |  |  |
|  |  |  | CCNE2 | -3.10 | 3.39E-03 |  |  |  |
|  |  |  | CENPK | -3.11 | 8.98E-04 |  |  |  |
|  |  |  | TICRR | -3.11 | 1.09E-03 |  |  |  |
|  |  |  | DMC1 | -3.12 | 1.19E-02 |  |  |  |
|  |  |  | EME1 | -3.12 | 4.11E-03 |  |  |  |
|  |  |  | RPL39L | -3.13 | 4.52E-04 |  |  |  |
|  |  |  | CPNE7 | -3.14 | 3.61E-03 |  |  |  |
|  |  |  | CKS2 | -3.14 | 2.69E-04 |  |  |  |
|  |  |  | NEURL1B | -3.15 | 1.63E-03 |  |  |  |
|  |  |  | RAD54L | -3.15 | 1.90E-04 |  |  |  |
|  |  |  | KIF15 | -3.15 | 1.62E-03 |  |  |  |
|  |  |  | STEAP1B | -3.17 | 8.27E-04 |  |  |  |
|  |  |  | SGOL1 | -3.17 | 3.34E-03 |  |  |  |
|  |  |  | FAM111B | -3.17 | 9.39E-03 |  |  |  |
|  |  |  | RP11-221N13.3 | -3.17 | 5.35E-03 |  |  |  |
|  |  |  | PKIB | -3.19 | 4.72E-02 |  |  |  |
|  |  |  | RAD51AP1 | -3.19 | 1.88E-03 |  |  |  |
|  |  |  | S100A3 | -3.19 | 1.40E-02 |  |  |  |
|  |  |  | TPPP3 | -3.20 | 1.87E-03 |  |  |  |
|  |  |  | CENPU | -3.20 | 1.98E-02 |  |  |  |
|  |  |  | DBNDD2 | -3.21 | 1.87E-03 |  |  |  |
|  |  |  | FBXO43 | -3.22 | 2.68E-02 |  |  |  |
|  |  |  | RP4-555D20.2 | -3.22 | 8.78E-03 |  |  |  |
|  |  |  | C11orf45 | -3.22 | 2.05E-02 |  |  |  |
|  |  |  | PKN3 | -3.23 | 3.14E-06 |  |  |  |
|  |  |  | AXL | -3.23 | 3.11E-07 |  |  |  |
|  |  |  | ZNF469 | -3.24 | 1.32E-02 |  |  |  |
|  |  |  | POLE2 | -3.24 | 2.94E-04 |  |  |  |
|  |  |  | NUSAP1 | -3.25 | 1.28E-03 |  |  |  |
|  |  |  | CORO1A | -3.26 | 4.86E-03 |  |  |  |
|  |  |  | TNFRSF11B | -3.27 | 4.87E-03 |  |  |  |
|  |  |  | CDK1 | -3.27 | 1.40E-03 |  |  |  |
|  |  |  | RACGAP1 | -3.28 | 2.15E-05 |  |  |  |
|  |  |  | CDC6 | -3.28 | 2.94E-04 |  |  |  |
|  |  |  | C11orf82 | -3.28 | 6.59E-04 |  |  |  |
|  |  |  | ADAMTS6 | -3.30 | 2.52E-06 |  |  |  |
|  |  |  | ASF1B | -3.30 | 6.22E-04 |  |  |  |
|  |  |  | PIM1 | -3.31 | 1.58E-02 |  |  |  |
|  |  |  | KIF4A | -3.32 | 1.11E-04 |  |  |  |
|  |  |  | DEPDC1B | -3.32 | 3.39E-04 |  |  |  |
|  |  |  | GSG2 | -3.32 | 4.39E-04 |  |  |  |
|  |  |  | E2F2 | -3.32 | 4.48E-03 |  |  |  |
|  |  |  | IL11 | -3.32 | 7.70E-04 |  |  |  |
|  |  |  | ESPL1 | -3.33 | 1.53E-03 |  |  |  |
|  |  |  | FLVCR2 | -3.33 | 2.51E-03 |  |  |  |
|  |  |  | ZWINT | -3.34 | 3.49E-05 |  |  |  |
|  |  |  | IL16 | -3.35 | 2.44E-02 |  |  |  |
|  |  |  | CDCA7 | -3.36 | 4.43E-05 |  |  |  |
|  |  |  | CCDC80 | -3.36 | 2.21E-04 |  |  |  |
|  |  |  | LYPD6B | -3.37 | 3.07E-04 |  |  |  |
|  |  |  | TOP2A | -3.37 | 1.03E-03 |  |  |  |
|  |  |  | CDT1 | -3.38 | 1.24E-05 |  |  |  |
|  |  |  | TM4SF1 | -3.38 | 1.68E-02 |  |  |  |
|  |  |  | CENPE | -3.39 | 1.63E-05 |  |  |  |
|  |  |  | DIRAS1 | -3.39 | 1.10E-04 |  |  |  |
|  |  |  | ANKRD20A5P | -3.40 | 6.92E-03 |  |  |  |
|  |  |  | OIP5 | -3.41 | 2.56E-03 |  |  |  |
|  |  |  | GINS2 | -3.41 | 5.59E-04 |  |  |  |
|  |  |  | KIRREL3 | -3.41 | 8.29E-05 |  |  |  |
|  |  |  | ENPP2 | -3.41 | 1.42E-02 |  |  |  |
|  |  |  | TACC3 | -3.41 | 3.49E-05 |  |  |  |
|  |  |  | TPX2 | -3.41 | 1.90E-04 |  |  |  |
|  |  |  | ESCO2 | -3.43 | 7.74E-03 |  |  |  |
|  |  |  | MND1 | -3.43 | 3.77E-03 |  |  |  |
|  |  |  | GREM2 | -3.43 | 1.23E-06 |  |  |  |
|  |  |  | KIF23 | -3.44 | 2.39E-06 |  |  |  |
|  |  |  | PNP | -3.44 | 1.78E-02 |  |  |  |
|  |  |  | APCDD1L | -3.45 | 5.42E-06 |  |  |  |
|  |  |  | TCEAL2 | -3.45 | 2.86E-02 |  |  |  |
|  |  |  | LFNG | -3.46 | 4.56E-02 |  |  |  |
|  |  |  | KIF11 | -3.47 | 4.29E-06 |  |  |  |
|  |  |  | POC1A | -3.48 | 4.38E-08 |  |  |  |
|  |  |  | CDCA3 | -3.48 | 2.59E-05 |  |  |  |
|  |  |  | KLF17 | -3.48 | 8.70E-03 |  |  |  |
|  |  |  | CCNF | -3.48 | 1.21E-05 |  |  |  |
|  |  |  | UCP2 | -3.50 | 1.25E-03 |  |  |  |
|  |  |  | RRAD | -3.50 | 8.90E-04 |  |  |  |
|  |  |  | SAPCD2 | -3.50 | 2.32E-04 |  |  |  |
|  |  |  | WNT5B | -3.51 | 9.39E-05 |  |  |  |
|  |  |  | MELK | -3.51 | 2.47E-06 |  |  |  |
|  |  |  | BAALC | -3.52 | 2.16E-04 |  |  |  |
|  |  |  | PLK4 | -3.53 | 2.66E-05 |  |  |  |
|  |  |  | CENPI | -3.54 | 1.37E-04 |  |  |  |
|  |  |  | CDCP1 | -3.55 | 6.75E-04 |  |  |  |
|  |  |  | CCBE1 | -3.55 | 2.58E-05 |  |  |  |
|  |  |  | RP11-2N1.2 | -3.56 | 3.50E-03 |  |  |  |
|  |  |  | SPAG5 | -3.56 | 6.44E-06 |  |  |  |
|  |  |  | PLEK2 | -3.57 | 1.34E-02 |  |  |  |
|  |  |  | UHRF1 | -3.57 | 6.93E-05 |  |  |  |
|  |  |  | RAD51 | -3.58 | 8.86E-05 |  |  |  |
|  |  |  | FST | -3.59 | 1.54E-02 |  |  |  |
|  |  |  | ORC1 | -3.59 | 3.06E-05 |  |  |  |
|  |  |  | VAC14-AS1 | -3.60 | 4.77E-02 |  |  |  |
|  |  |  | NCAPG | -3.60 | 1.44E-04 |  |  |  |
|  |  |  | TRABD2A | -3.60 | 2.16E-04 |  |  |  |
|  |  |  | APCDD1L-AS1 | -3.60 | 1.89E-02 |  |  |  |
|  |  |  | KIF14 | -3.63 | 1.54E-04 |  |  |  |
|  |  |  | NXN | -3.63 | 6.73E-06 |  |  |  |
|  |  |  | WDR62 | -3.63 | 4.53E-06 |  |  |  |
|  |  |  | CDC45 | -3.63 | 1.90E-04 |  |  |  |
|  |  |  | TRIM7 | -3.65 | 9.91E-06 |  |  |  |
|  |  |  | HRH1 | -3.65 | 1.60E-02 |  |  |  |
|  |  |  | PBK | -3.65 | 5.63E-05 |  |  |  |
|  |  |  | EXO1 | -3.66 | 2.15E-05 |  |  |  |
|  |  |  | IL6 | -3.66 | 9.77E-03 |  |  |  |
|  |  |  | PTTG1 | -3.67 | 7.99E-05 |  |  |  |
|  |  |  | MKI67 | -3.67 | 2.11E-03 |  |  |  |
|  |  |  | CDCA5 | -3.68 | 5.24E-06 |  |  |  |
|  |  |  | SEMA7A | -3.68 | 1.93E-03 |  |  |  |
|  |  |  | S100A4 | -3.69 | 3.02E-04 |  |  |  |
|  |  |  | ARHGAP11A | -3.69 | 1.75E-07 |  |  |  |
|  |  |  | XKR5 | -3.69 | 5.91E-04 |  |  |  |
|  |  |  | PRC1 | -3.69 | 2.59E-05 |  |  |  |
|  |  |  | NRXN2 | -3.70 | 8.95E-04 |  |  |  |
|  |  |  | KIF2C | -3.71 | 1.00E-06 |  |  |  |
|  |  |  | SLC29A1 | -3.71 | 1.16E-04 |  |  |  |
|  |  |  | KIF20A | -3.74 | 5.55E-05 |  |  |  |
|  |  |  | SHROOM2 | -3.75 | 1.09E-04 |  |  |  |
|  |  |  | GCNT4 | -3.75 | 3.70E-04 |  |  |  |
|  |  |  | MCM10 | -3.76 | 2.87E-04 |  |  |  |
|  |  |  | MATN2 | -3.77 | 6.54E-06 |  |  |  |
|  |  |  | ELN | -3.77 | 1.97E-05 |  |  |  |
|  |  |  | TROAP | -3.78 | 3.39E-06 |  |  |  |
|  |  |  | RP11-424C20.2 | -3.79 | 2.60E-04 |  |  |  |
|  |  |  | BUB1B | -3.81 | 6.49E-05 |  |  |  |
|  |  |  | MARCH4 | -3.82 | 4.50E-04 |  |  |  |
|  |  |  | KIF18B | -3.83 | 1.47E-04 |  |  |  |
|  |  |  | CENPW | -3.84 | 6.40E-06 |  |  |  |
|  |  |  | NUF2 | -3.85 | 2.97E-04 |  |  |  |
|  |  |  | CASC5 | -3.85 | 3.42E-04 |  |  |  |
|  |  |  | NCAPH | -3.86 | 3.08E-06 |  |  |  |
|  |  |  | HMMR | -3.86 | 5.07E-05 |  |  |  |
|  |  |  | SKA3 | -3.87 | 3.97E-05 |  |  |  |
|  |  |  | IL12A | -3.87 | 4.47E-03 |  |  |  |
|  |  |  | NEIL3 | -3.89 | 2.22E-04 |  |  |  |
|  |  |  | RP11-758N13.1 | -3.89 | 1.28E-04 |  |  |  |
|  |  |  | STEAP1 | -3.89 | 9.95E-06 |  |  |  |
|  |  |  | CKAP2L | -3.91 | 9.09E-05 |  |  |  |
|  |  |  | NEK2 | -3.93 | 9.30E-05 |  |  |  |
|  |  |  | FAM180A | -3.93 | 6.49E-04 |  |  |  |
|  |  |  | HJURP | -3.93 | 1.15E-04 |  |  |  |
|  |  |  | SNORD17 | -3.96 | 1.63E-04 |  |  |  |
|  |  |  | SUSD3 | -3.97 | 1.09E-05 |  |  |  |
|  |  |  | S100A2 | -3.97 | 3.83E-04 |  |  |  |
|  |  |  | C6orf132 | -3.98 | 1.69E-03 |  |  |  |
|  |  |  | LINC01119 | -3.99 | 4.27E-05 |  |  |  |
|  |  |  | IQGAP3 | -4.00 | 2.13E-05 |  |  |  |
|  |  |  | CENPM | -4.01 | 5.89E-05 |  |  |  |
|  |  |  | SPC25 | -4.01 | 1.00E-04 |  |  |  |
|  |  |  | DEPDC1 | -4.02 | 5.04E-05 |  |  |  |
|  |  |  | CASC10 | -4.02 | 1.20E-02 |  |  |  |
|  |  |  | APOBEC3B | -4.02 | 2.37E-04 |  |  |  |
|  |  |  | UBE2C | -4.02 | 2.67E-05 |  |  |  |
|  |  |  | CDC25C | -4.03 | 7.21E-05 |  |  |  |
|  |  |  | CDKN3 | -4.03 | 2.52E-06 |  |  |  |
|  |  |  | GTSE1 | -4.04 | 8.62E-06 |  |  |  |
|  |  |  | CGREF1 | -4.05 | 2.43E-04 |  |  |  |
|  |  |  | CDCA2 | -4.05 | 6.74E-05 |  |  |  |
|  |  |  | KCNS1 | -4.06 | 4.39E-04 |  |  |  |
|  |  |  | TRIP13 | -4.06 | 1.46E-05 |  |  |  |
|  |  |  | DNAH5 | -4.08 | 2.58E-03 |  |  |  |
|  |  |  | CCNB1 | -4.10 | 9.11E-07 |  |  |  |
|  |  |  | KIAA0101 | -4.11 | 5.18E-06 |  |  |  |
|  |  |  | PLK1 | -4.12 | 1.23E-06 |  |  |  |
|  |  |  | HHIPL2 | -4.12 | 1.25E-03 |  |  |  |
|  |  |  | SH2D5 | -4.13 | 3.07E-03 |  |  |  |
|  |  |  | NFASC | -4.13 | 3.04E-06 |  |  |  |
|  |  |  | KIFC1 | -4.15 | 4.95E-06 |  |  |  |
|  |  |  | RAMP1 | -4.15 | 4.25E-04 |  |  |  |
|  |  |  | SHCBP1 | -4.15 | 1.60E-05 |  |  |  |
|  |  |  | SKA1 | -4.16 | 3.95E-05 |  |  |  |
|  |  |  | MYBL2 | -4.17 | 5.26E-06 |  |  |  |
|  |  |  | AURKA | -4.17 | 1.33E-06 |  |  |  |
|  |  |  | ERCC6L | -4.17 | 1.66E-04 |  |  |  |
|  |  |  | CASZ1 | -4.17 | 5.73E-04 |  |  |  |
|  |  |  | TTK | -4.18 | 3.43E-06 |  |  |  |
|  |  |  | ELOVL2 | -4.18 | 4.39E-03 |  |  |  |
|  |  |  | E2F8 | -4.20 | 7.12E-04 |  |  |  |
|  |  |  | FOXM1 | -4.21 | 5.80E-06 |  |  |  |
|  |  |  | LINC01085 | -4.22 | 3.34E-04 |  |  |  |
|  |  |  | CCNA2 | -4.27 | 6.37E-07 |  |  |  |
|  |  |  | HAPLN1 | -4.29 | 2.24E-07 |  |  |  |
|  |  |  | BUB1 | -4.30 | 5.46E-06 |  |  |  |
|  |  |  | CDCA8 | -4.31 | 6.17E-06 |  |  |  |
|  |  |  | ANLN | -4.32 | 8.50E-06 |  |  |  |
|  |  |  | ATP10A | -4.32 | 2.22E-06 |  |  |  |
|  |  |  | DLX5 | -4.32 | 2.17E-02 |  |  |  |
|  |  |  | DLGAP5 | -4.36 | 1.21E-05 |  |  |  |
|  |  |  | MLPH | -4.38 | 1.50E-03 |  |  |  |
|  |  |  | FAM83D | -4.41 | 1.53E-05 |  |  |  |
|  |  |  | AC087645.1 | -4.42 | 2.76E-03 |  |  |  |
|  |  |  | KYNU | -4.43 | 1.28E-05 |  |  |  |
|  |  |  | SPC24 | -4.47 | 5.87E-06 |  |  |  |
|  |  |  | PKMYT1 | -4.48 | 3.65E-06 |  |  |  |
|  |  |  | TEK | -4.49 | 1.30E-03 |  |  |  |
|  |  |  | CDC20 | -4.50 | 6.51E-06 |  |  |  |
|  |  |  | ABI3BP | -4.51 | 1.10E-04 |  |  |  |
|  |  |  | NUAK2 | -4.51 | 1.61E-06 |  |  |  |
|  |  |  | CEP55 | -4.52 | 3.65E-06 |  |  |  |
|  |  |  | CCNB2 | -4.53 | 1.36E-06 |  |  |  |
|  |  |  | TK1 | -4.56 | 5.76E-08 |  |  |  |
|  |  |  | METTL7B | -4.57 | 4.28E-05 |  |  |  |
|  |  |  | CENPA | -4.57 | 1.06E-06 |  |  |  |
|  |  |  | SERPINB7 | -4.57 | 4.43E-05 |  |  |  |
|  |  |  | MOK | -4.57 | 1.75E-07 |  |  |  |
|  |  |  | RRM2 | -4.58 | 1.37E-05 |  |  |  |
|  |  |  | BIRC5 | -4.60 | 1.33E-06 |  |  |  |
|  |  |  | SERINC2 | -4.62 | 3.69E-06 |  |  |  |
|  |  |  | CALB2 | -4.63 | 7.25E-03 |  |  |  |
|  |  |  | AURKB | -4.66 | 3.36E-06 |  |  |  |
|  |  |  | DMKN | -4.69 | 5.98E-04 |  |  |  |
|  |  |  | SPOCD1 | -4.69 | 3.75E-09 |  |  |  |
|  |  |  | FAM64A | -4.70 | 1.73E-05 |  |  |  |
|  |  |  | MICAL2 | -4.75 | 2.77E-07 |  |  |  |
|  |  |  | OLFM1 | -4.76 | 2.11E-05 |  |  |  |
|  |  |  | NOS3 | -4.84 | 5.12E-04 |  |  |  |
|  |  |  | FGF5 | -4.89 | 2.93E-06 |  |  |  |
|  |  |  | FAM132B | -4.92 | 8.85E-05 |  |  |  |
|  |  |  | FAIM3 | -4.99 | 1.29E-04 |  |  |  |
|  |  |  | IL7R | -5.00 | 5.76E-10 |  |  |  |
|  |  |  | DLX3 | -5.01 | 9.08E-04 |  |  |  |
|  |  |  | DLX4 | -5.02 | 1.86E-04 |  |  |  |
|  |  |  | PSG4 | -5.03 | 1.17E-06 |  |  |  |
|  |  |  | RAB27B | -5.03 | 9.82E-06 |  |  |  |
|  |  |  | RP11-824M15.3 | -5.04 | 7.63E-03 |  |  |  |
|  |  |  | AC114494.1 | -5.11 | 8.78E-04 |  |  |  |
|  |  |  | PLCXD3 | -5.17 | 4.27E-04 |  |  |  |
|  |  |  | KRT15 | -5.18 | 7.52E-03 |  |  |  |
|  |  |  | DLX6 | -5.18 | 4.69E-02 |  |  |  |
|  |  |  | C5AR2 | -5.18 | 9.54E-04 |  |  |  |
|  |  |  | TMEM155 | -5.25 | 1.11E-03 |  |  |  |
|  |  |  | TNC | -5.26 | 2.23E-07 |  |  |  |
|  |  |  | PAQR5 | -5.30 | 4.70E-07 |  |  |  |
|  |  |  | MUC12 | -5.30 | 9.97E-04 |  |  |  |
|  |  |  | MGAM | -5.32 | 7.85E-03 |  |  |  |
|  |  |  | NLRP10 | -5.35 | 8.41E-04 |  |  |  |
|  |  |  | RP11-47I22.3 | -5.41 | 1.49E-05 |  |  |  |
|  |  |  | PKP1 | -5.44 | 4.48E-05 |  |  |  |
|  |  |  | TMEM255A | -5.45 | 3.20E-05 |  |  |  |
|  |  |  | RP11-395B7.4 | -5.48 | 6.19E-05 |  |  |  |
|  |  |  | SIGLEC15 | -5.53 | 5.59E-06 |  |  |  |
|  |  |  | TM4SF20 | -5.55 | 1.89E-04 |  |  |  |
|  |  |  | MYPN | -5.60 | 3.67E-08 |  |  |  |
|  |  |  | OXTR | -5.60 | 1.25E-08 |  |  |  |
|  |  |  | GDF5OS | -5.63 | 8.95E-04 |  |  |  |
|  |  |  | KRTAP1-1 | -5.68 | 4.27E-04 |  |  |  |
|  |  |  | SOX11 | -5.71 | 1.24E-04 |  |  |  |
|  |  |  | GDF5 | -5.79 | 4.92E-05 |  |  |  |
|  |  |  | CATSPER1 | -5.88 | 1.29E-04 |  |  |  |
|  |  |  | HR | -5.91 | 4.67E-04 |  |  |  |
|  |  |  | ATP8B1 | -6.02 | 6.24E-10 |  |  |  |
|  |  |  | KRT33B | -6.09 | 3.76E-07 |  |  |  |
|  |  |  | CST6 | -6.12 | 4.43E-06 |  |  |  |
|  |  |  | RP11-527H14.3 | -6.12 | 6.14E-07 |  |  |  |
|  |  |  | DIO3 | -6.19 | 5.26E-04 |  |  |  |
|  |  |  | CTC-436P18.3 | -6.24 | 2.11E-05 |  |  |  |
|  |  |  | SCN2B | -6.33 | 1.34E-03 |  |  |  |
|  |  |  | LINC00968 | -6.40 | 1.48E-05 |  |  |  |
|  |  |  | CTNND2 | -6.40 | 1.28E-05 |  |  |  |
|  |  |  | WNT7B | -6.40 | 2.59E-03 |  |  |  |
|  |  |  | RP11-492E3.2 | -6.45 | 1.00E-06 |  |  |  |
|  |  |  | MASP1 | -6.46 | 1.37E-04 |  |  |  |
|  |  |  | SCN4B | -6.49 | 4.27E-04 |  |  |  |
|  |  |  | NOG | -6.52 | 7.88E-05 |  |  |  |
|  |  |  | LRRC2 | -6.57 | 8.43E-04 |  |  |  |
|  |  |  | GIPR | -6.60 | 9.87E-06 |  |  |  |
|  |  |  | GREM1 | -6.86 | 4.00E-07 |  |  |  |
|  |  |  | KRT14 | -6.89 | 1.63E-04 |  |  |  |
|  |  |  | LINC00856 | -6.93 | 2.22E-06 |  |  |  |
|  |  |  | NPTX2 | -6.93 | 5.51E-06 |  |  |  |
|  |  |  | SP6 | -6.96 | 4.53E-05 |  |  |  |
|  |  |  | KRT80 | -7.21 | 5.42E-06 |  |  |  |
|  |  |  | LRRC15 | -7.29 | 2.23E-07 |  |  |  |
|  |  |  | GFRA2 | -7.34 | 2.39E-07 |  |  |  |
|  |  |  | CLEC3B | -7.40 | 6.10E-06 |  |  |  |
|  |  |  | KRT7 | -7.45 | 4.36E-06 |  |  |  |
|  |  |  | KRT34 | -7.50 | 9.11E-07 |  |  |  |
|  |  |  | KRT19 | -7.55 | 5.24E-06 |  |  |  |
|  |  |  | SYNPO2L | -7.73 | 5.01E-07 |  |  |  |
|  |  |  | KISS1 | -7.84 | 2.79E-08 |  |  |  |
|  |  |  | PSG5 | -8.18 | 2.53E-09 |  |  |  |
|  |  |  | KRTAP2-3 | -8.27 | 9.87E-06 |  |  |  |
|  |  |  | LINC00707 | -8.31 | 1.75E-07 |  |  |  |
|  |  |  | KRTAP1-5 | -8.78 | 3.94E-04 |  |  |  |
|  |  |  | PENK | -9.42 | 9.40E-06 |  |  |  |
|  |  |  | SBSN | -9.54 | 5.93E-06 |  |  |  |
|  |  |  | PTPRN | -9.96 | 6.46E-08 |  |  |  |
